# Copper‐Catalyzed Borylative Cross‐Coupling of Allenes and Imines: Selective Three‐Component Assembly of Branched Homoallyl Amines

**DOI:** 10.1002/anie.201508959

**Published:** 2015-12-03

**Authors:** James Rae, Kay Yeung, Joseph J. W. McDouall, David J. Procter

**Affiliations:** ^1^ School of ChemistryUniversity of ManchesterOxford RdManchesterM13 9PLUK

**Keywords:** allenes, boron, copper, cross-coupling, imines

## Abstract

A copper‐catalyzed three‐component coupling of allenes, bis(pinacolato)diboron, and imines allows regio‐, chemo‐, and diastereoselective assembly of branched α,β‐substituted‐γ‐boryl homoallylic amines, that is, products bearing versatile amino, alkenyl, and borane functionality. Alternatively, convenient oxidative workup allows access to α‐substituted‐β‐amino ketones. A computational study has been used to probe the stereochemical course of the cross‐coupling.

Branched amines are common motifs in biologically active molecules. Substituted homoallylic amines are privileged synthetic precursors to such motifs in addition to being a common substructure in bioactive synthetic targets in their own right (Scheme 1 a).^1^ While the allylation of imines provides the most direct access to important homoallylic amines,^2^, ^3^ the process is generally more challenging than the allylation of aldehydes because of the lower electrophilicity/reactivity of imines and their increased steric bulk, difficulties in predicting the stereochemical outcome of additions, and imine/enamine *E/Z* isomerization. In particular, the additions of substituted allyl metals require sophisticated levels of regio‐ and stereocontrol, which, if unchecked, lead to complex mixtures.^4^ Thus, new approaches to the selective generation and controlled addition of functionalized allyl metals, formed under catalytic conditions using inexpensive catalysts, to imines, promises to expand access to complex homoallylic amines while unlocking new avenues for their subsequent synthetic manipulation.

**Scheme 1:**
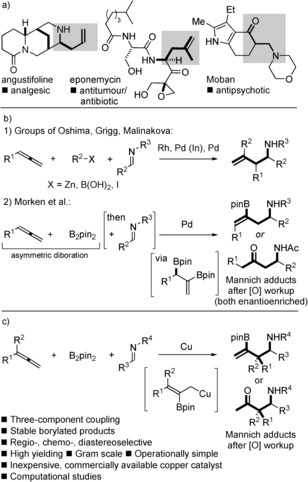
a) Homoallylic amines and their derivatives, in biologically significant targets. b) Previous work: Metal‐catalyzed, three‐component, couplings involving allenes and imines. c) This work: Copper‐catalyzed, highly selective, three‐component, borylative allylation of imines. Pin=pinacolato.

The use of allenes and imines in catalytic three‐component coupling reactions which involve the in situ generation of allyl metals has emerged as an exciting strategy to afford valuable, substituted homoallylic amines (Scheme 1 b).^5^, ^6^, ^7^ For example, the groups of Oshima,5e Grigg,5a–5d and Malinakova5f–5i have studied the catalytic generation of allyl metals from allenes, by organometallic addition, and their addition to imines. In an important advance, Morken and co‐workers described the asymmetric palladium‐catalyzed diboration of allenes with subsequent addition of an imine to trap the functionalized allyl borane intermediates in a separate operation.5j In the study from Morken et al., linear adducts were obtained and products were typically isolated as ketones after in situ oxidation of the C−B bond. In the above studies, expensive and supply‐risk platinum‐group metals were used.^5^ Herein we describe a regio‐, chemo‐, and diastereoselective copper‐catalyzed three‐component coupling of allenes,^8^ bis(pinacolato)diboron, and imines,^9^, ^10^ proceeding via allyl coppers to give readily isolable, branched homoallylic amines bearing versatile amino, alkenyl, and borane functionalities (Scheme 1 c).

Faced with the challenge of controlling the regioselectivity of borylcopper addition to allenes, it was clear that the use of a well‐defined, bench‐stable, and commercially available pre‐catalyst would be highly desirable. Optimization of the process commenced with using 5 mol % IPrCuCl, the allene **1 a**, 6 mol % of KO*t*Bu, and 1.1 equivalents of B_2_pin_2_ in the borylative cross‐coupling with the N‐phenyl imine **2 a**. The corresponding homoallylic amine **3 a** was obtained with 27 % conversion (Table 1, entry 1). Pleasingly, increasing the amount of base to 1 equivalent (entries 2–4) resulted in near complete conversion to give **3 a**. Although the base could be changed (entry 5; the use of Cs_2_CO_3_ gave 41 % conversion), the use of KO*t*Bu appeared optimal. Pleasingly, the allene **1 b**, also underwent efficient borylative cross‐coupling with **2 a** under these reaction conditions (entry 6) and temperature was found to influence the diastereoselectivity of three‐component coupling (entries 7 and 8). By initiating the borylative cross‐coupling of **1 b** and **2 a** at −78 °C (entry 8), quantitative conversion into **3 b** was observed and the homoallylic amine was obtained in 85:15 d.r.

**Table 1:** Optimization of the copper‐catalyzed borylative cross‐coupling of allenes and imines.^[a]^ 
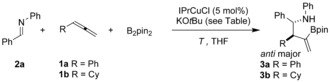

Entry	Product	*T* [°C]	*t* [h]	KO*t*Bu (equiv)	d.r.^[b]^	Conv. [%]^[c]^
1	**3 a**	RT	18	0.06	57:43	27
2	**3 a**	RT	18	0.3	64:36	62
3	**3 a**	RT	18	0.7	63:37	75
4	**3 a**	RT	18	1.0	62:38	94
5^[d]^	**3 a**	RT	18	0.06	63:37	41
6	**3 b**	RT	1.5	1.0	77:23	100
7	**3 b**	0	3	1.0	82:18	90
8	**3 b**	−78 to RT	18	1.0	85:15	100

[a] Reaction conditions: Allene (1.5 equiv), B_2_pin_2_ (1.1 equiv), imine (1 equiv). [b] Determined by ^1^H NMR analysis of the crude product mixture. [c] Determined by ^1^H NMR analysis of the crude product mixture using an internal standard. [d] 1 equiv of Cs_2_CO_3_ added. THF=tetrahydrofuran.

With optimized reaction conditions in hand, we evaluated the scope of the reaction. In general, the reaction proved to be high yielding, and a wide range of allene (**1**) and imine (**2**) coupling partners were converted into the corresponding homoallylic amines **3** with complete regiocontrol and with up to greater than 98 % diastereocontrol (Scheme 2). A range of allenes was tolerated, including substrates bearing linear alkyl (to give **3 c**, **3 d**, and **3 u**), aryl (to give **3 a**), and 1,1‐dialkyl (to give **3 p**–**t** and **3 v**) substituents. 1,1‐Disubstitution in the starting allene allowed assembly of homoallylic amines, bearing quaternary centers, in a selective three‐component coupling. The common *para*‐methoxyphenyl (PMP) protecting group for the nitrogen atom was incorporated in a range of aryl‐substituted imines and proved compatible with the cross‐coupling process (to give **3 e**–**q** and **3 s**–**u**). The borylative cross‐coupling showed good functional‐group tolerance with OMe (**3 f** and **3 p**), CF_3_ (**3 g**), Br (**3 h** and **3 q**), thienyl (**3 j** and **3 t**), and furanyl (**3 i** and **3 s**) groups proving compatible with the reaction conditions. When the steric bulk of the imine substituent R^1^ was increased, higher diastereoselectivities were noted, with *i*Pr‐ (**3 n**), *t*Bu‐ (**3 o**), 1‐naphthyl‐ (**3 k**), and *o*‐tolyl‐substituted (**3 m**) products generated with excellent diastereoselectivity (between 94:6 and >98:2 d.r.). The influence of the imine substituent R^1^ is best illustrated by comparing the assembly of the products **3 d** and **3 u**: the use of an *o*‐tolyl‐substituted imine increases the diastereoselectivity of the process from 79:21 to 92:8 d.r. Interestingly, the use of a *tert‐*butylimine and an isopropylimine (to give **3 o** and **3 n**, respectively) resulted in low yields of the isolated products, while the attempted use of a mesityl imine resulted in no reaction. Thus, the reaction is significantly slowed by the use of bulky groups on the imine. This slower reaction is likely a result of the large substituents diminishing the rate of the transmetallation event which closes the catalytic cycle and involves a Cu−N species and B_2_pin_2_ (see below).

**Scheme 2:**
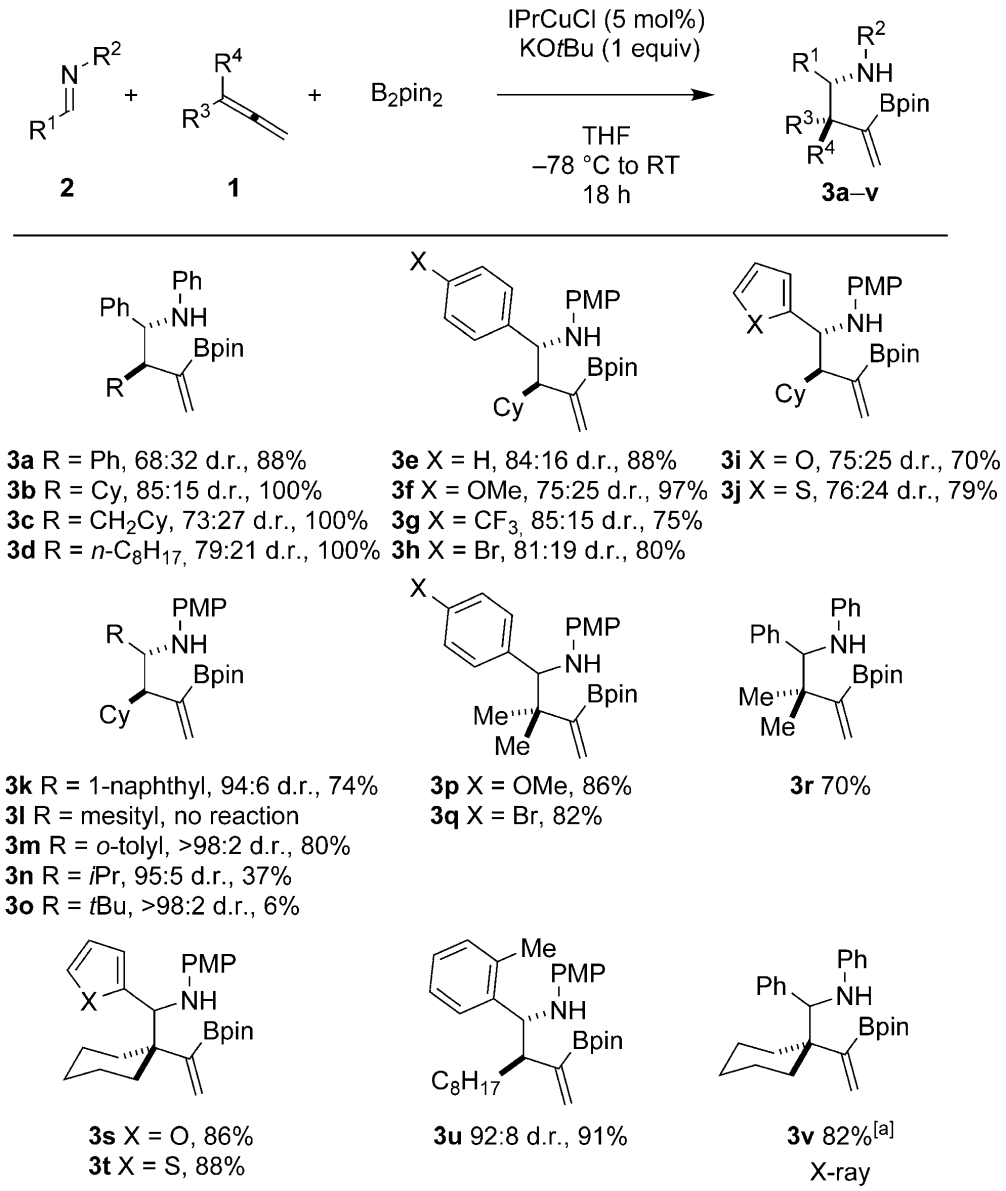
Substrate scope in the copper‐catalyzed borylative allylation of imines. Reaction conditions: Allene (1.5 equiv), B_2_pin_2_ (1.1 equiv), imine (1 equiv). Yields are those of the isolated products. [a] See Figure 2 a for X‐ray structure. PMP=*para*‐methoxyphenyl.

Pleasingly, the use of N‐aryl imines afforded three‐component coupling products (**3**) which were stable to silica gel column chromatography, and showed no signs of proto‐deborylation, a common issue for vinylboron‐containing compounds.^11^ In the case of adducts arising from the use of N‐benzyl imines, products decomposed on silica, and were best isolated as the analogous methyl ketones after oxidative workup (Figure 1). Thus, α,β‐substituted Mannich adducts were also available from the copper‐catalyzed three‐component coupling in good to excellent yields and with high diastereocontrol.^12^

**Figure 1:**
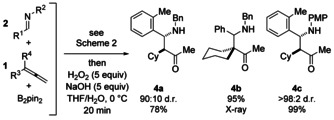
Copper‐catalyzed three‐component assembly of Mannich bases.

The suitability of the copper‐catalyzed three‐component coupling for preparative scale synthesis was next investigated. Pleasingly, upon cross‐coupling with B_2_pin_2_ and **1 b**, 0.5 grams (2.2 mmol) of **2 b** gave 1.0 gram of the functionalized homoallylic amine **3 m** in 97 % yield and it was essentially isolated as a single diastereoisomer (Scheme 3).

**Scheme 3:**
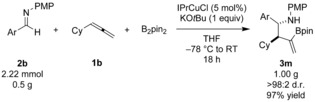
Preparative scale copper‐catalyzed borylative allylation of an imine. Ar=*o*‐tolyl.

^11^B NMR analysis of functionalized homoallylic amine products **3** (^11^B: *δ*=−2.9 to 11 ppm; ^11^B *δ*=35 ppm for a typical Bpin group) indicates an sp^3^‐hybridized boron center resulting from dative coordination of the N_*n*_→B_*p*_ in solution.^13^ However, X‐ray crystallographic analysis of **3 v** suggested that the B–N interaction is disrupted in the solid state (Figure 2 a). The B–N interaction could also be broken by protonation of the nitrogen atom, that is, treatment of **3 m** with 1 equivalent of trifluoromethane sulfonic acid afforded the salt **5 a** (Figure 2 b).

**Figure 2:**
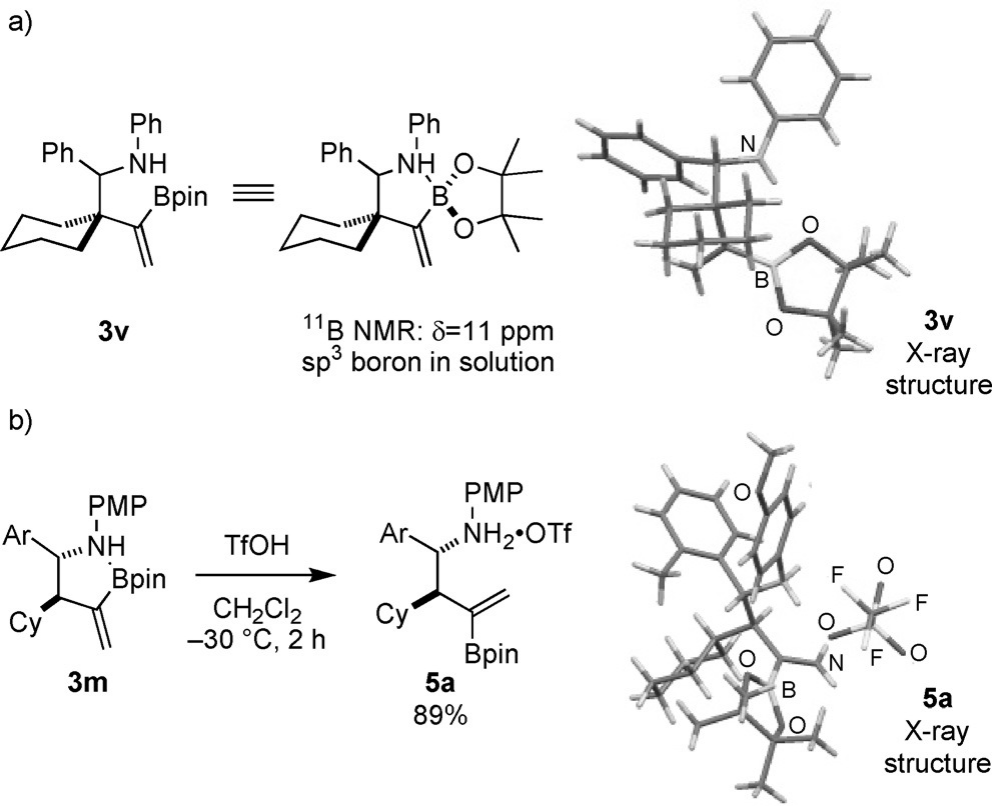
a) ^11^B NMR data and X‐ray crystallographic^21^ analysis of **3 v**. b) Formation of a salt of **3 m** by disrupting the the N_*n*_→B_*p*_ interaction. Ar=*o*‐tolyl, Tf=trifluoromethanesulfonyl.

Interestingly, N‐sulfinyl and N‐sulfonyl imines were unreactive under the reaction conditions of the copper‐catalyzed three‐component coupling. In a mechanistic study designed to explore this observation, no conversion was observed in an experiment involving both **2 a** and the N‐tosyl imine **2 c** (Scheme 4 a). Both imines were detected unchanged in the ^1^H NMR spectra of the crude reaction mixture. An unproductive coordination of **2 c** to copper appears to prevent reaction of the boryl copper intermediate with the allene. To probe this further, a stoichiometric amount of the IPrCl was used to generate the allylcopper intermediate **6**. Addition of **2 c** to the preformed **6** generated the expected adduct **3 w** in 56 % yield (Scheme 4 b). This control experiment shows that allylcopper intermediates are efficiently trapped by N‐sulfonyl imines and suggests that catalyst inhibition by the imine, possibly by formation of the coordination adduct **7**, in which the N‐tosyl imine chelates to the copper catalyst through both the nitrogen and oxygen atoms, lies behind the lack of reactivity observed with these imines.^14^

**Scheme 4:**
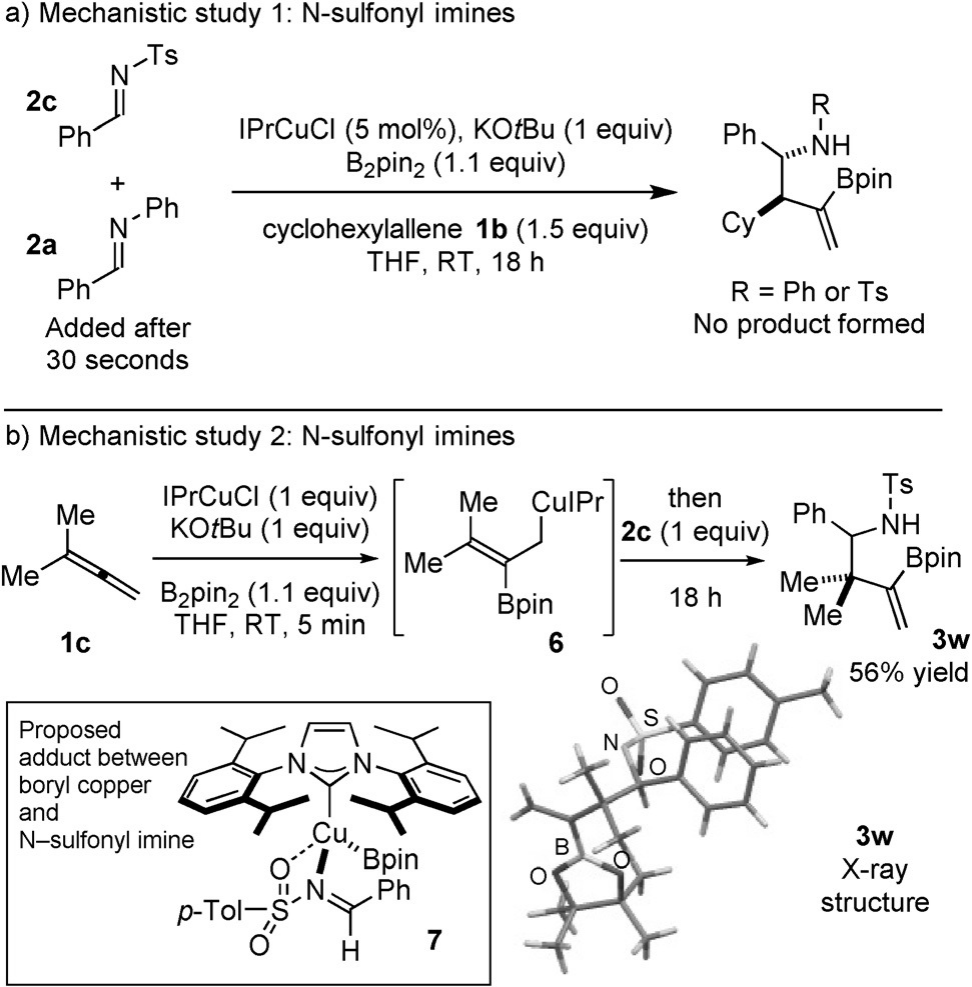
a) The presence of an N‐sulfonyl imine inhibits coupling with an N‐phenyl imine. b) A preformed, functionalized allyl copper undergoes coupling with an N‐sulfonyl imine.^21^ Ts=4‐toluenesulfonyl.

A proposed mechanism for the copper‐catalyzed three‐component coupling is shown in Scheme 5. After initial formation of the ligated copper alkoxide **I**, transmetallation with B_2_pin_2_ yields **II**. Regioselective insertion of the allene into the Cu−B bond then gives the allylcopper **III**, which can undergo diastereoselective addition to the imine to afford the homoallylic amine **IV**. Base‐assisted transmetallation then regenerates **II** and yields **V**, which upon workup gives the homoallylic amines **3** after protonation.

**Scheme 5:**
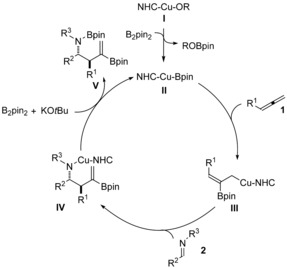
A proposed catalytic cycle for the copper‐catalyzed borylative allylation of imines.

The observed *anti* diastereoselectivity of the cross‐coupling may arise from the addition of *Z*‐allyl copper **III** through either the six‐membered chair transition structure (TS) **8 a**, in which copper interacts with the imine nitrogen atom, or through the half‐chair‐like transition structure **8 b**, in which the imine nitrogen atom interacts with boron (Scheme 6).

**Scheme 6:**
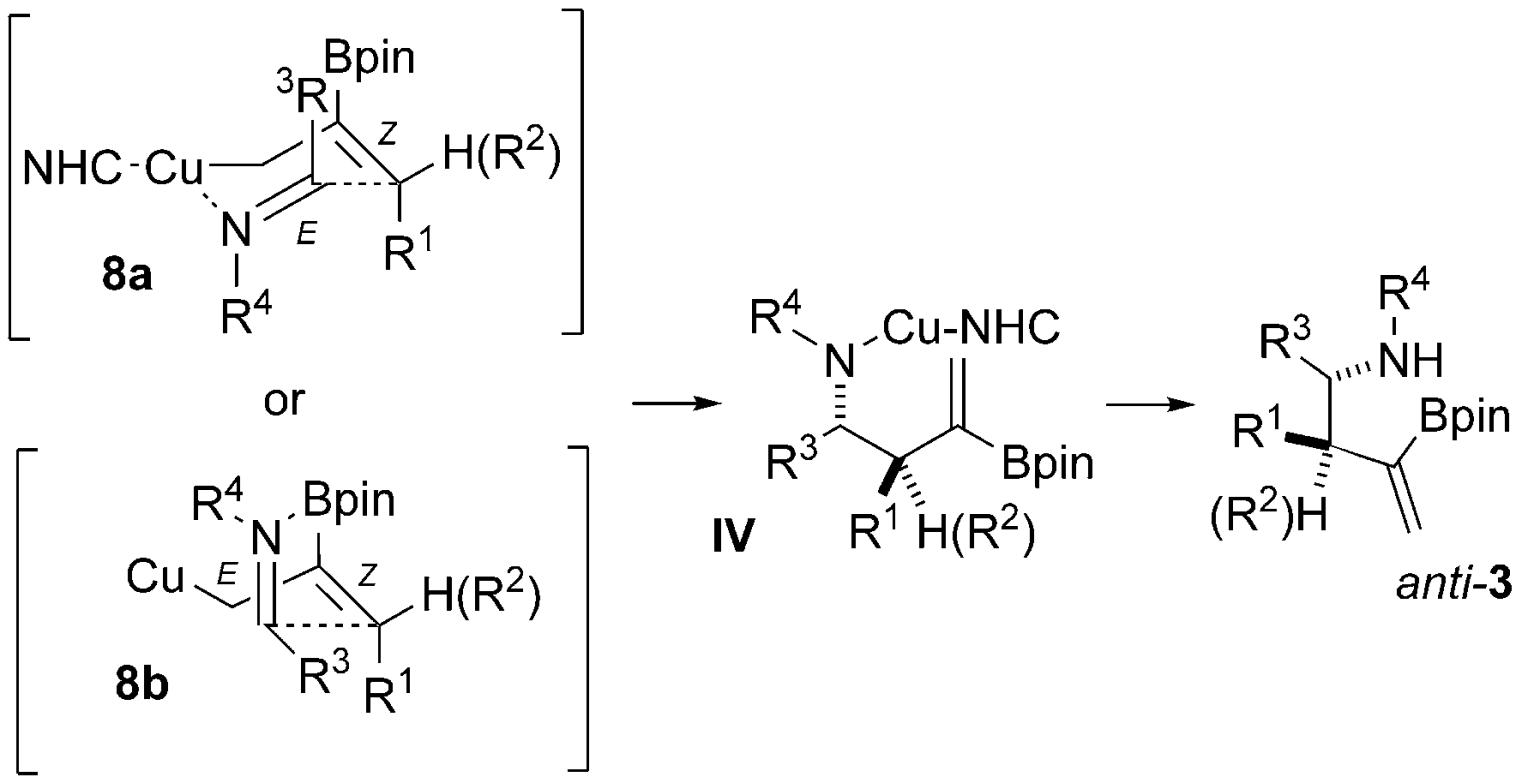
Possible origin of diastereocontrol in the borylative cross‐coupling of allenes and imines.

To explore the origin of the diastereoselectivity further, we have carried out computational studies on the coupling of the model imine **A** and allylcopper **B** (Scheme 7). Geometries were fully optimized in the gas phase and also in THF solvent.^15^, ^16^ As **A** and **B** approach each other, the potential energy decreases until the minimum energy structure (**C**) is formed through interaction of copper with the nitrogen atom. In the gas phase this structure lies 29.6 kJ mol^−1^ in energy lower than the separated reactants. From the intermediate **C**, a six‐membered transition‐state structure is formed (**D**; see **8 a** in Scheme 6). The energy barrier to the formation of **D** is only 10.0 kJ mol^−1^ (THF). After the formation of **D**, the *anti* product **E**, with the Cu atom coordinated centrally over the C=C bond, is formed with an exothermicity of about 113 kJ mol^−1^.

**Scheme 7:**
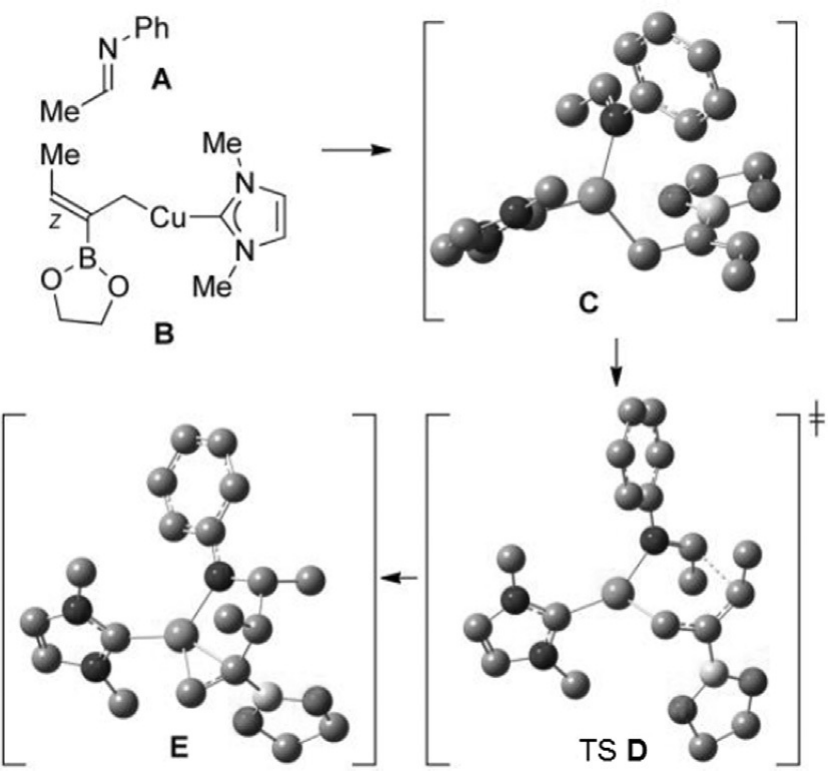
Computational study on the coupling of model substrates **A** and **B**. Hydrogen atoms have been omitted for clarity.

The computational studies clearly suggest that the *anti* selectivity of the copper‐catalyzed three‐component coupling arises from the interaction of copper with the imine nitrogen atom and the formation of **8 a** (see transition structure **D** in Scheme 7), despite several substituents occupying pseudoaxial orientations.^17^ Initial interaction of the imine nitrogen atom with boron is calculated to be less favorable and there is a large barrier to carbon–carbon bond formation via the alternative half‐chair‐like transition structure **8 b** (see the Supporting Information).

Finally, a preliminary study illustrates that the three‐component approach to functionalized homoallylic amines can be extended to the silylative cross‐coupling of allenes and imines.^18^ Employing Suginome's reagent, Me_2_PhSiBpin,^19^ in a related copper‐catalyzed assembly involving **1 a** and **2 a** gave the homoallylic amine **3 x** with complete regiocontrol, high diastereocontrol, and in good yield (Scheme 8).

**Scheme 8:**
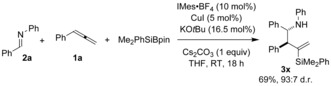
Copper‐catalyzed silylative allylation of an imine. Me_2_PhSiBpin (1.1 equiv).

In summary, the first copper‐catalyzed three‐component coupling of allenes, imines, and bis(pinacolato)diboron furnishes stable borylated homoallylic amines, or Mannich‐type products after oxidative work‐up, with high control. The process utilizes a commercially available copper catalyst, tolerates a range of allene and imine building blocks, and affords complex homoallylic amine products in high yield with excellent regiocontrol and high diastereocontrol.^20^ Computational studies suggest the diastereoselectivity of the coupling arises from imine complexation to the copper of the allylcopper intermediate and addition through a six‐membered chair transition structure.

## Supporting information

As a service to our authors and readers, this journal provides supporting information supplied by the authors. Such materials are peer reviewed and may be re‐organized for online delivery, but are not copy‐edited or typeset. Technical support issues arising from supporting information (other than missing files) should be addressed to the authors.

Supplementary
