# Supplementary material for: Copper‐Catalyzed Borylative Cross‐Coupling of Allenes and Imines: Selective Three‐Component Assembly of Branched Homoallyl Amines
Source: Angew Chem Int Ed Engl. 2015 Dec 3;55(3):1102–7. doi: 10.1002/anie.201508959 (PMC4736445; doi:10.1002/anie.201508959)
Supplement: Supplementary file 1 — Supplementary [file ANIE-55-1102-s001.pdf]

Angewandte Chemie
Eine Zeitschrift der Gesellschaft Deutscher Chemiker

Supporting Information

Copper-Catalyzed Borylative Cross-Coupling of Allenes and Imines: Selective Three-Component Assembly of Branched Homoallyl Amines

*James Rae, Kay Yeung, Joseph J. W. McDouall, and David J. Procter**

anie_201508959_sm_miscellaneous_information.pdf

Contents

General Information S2

General Procedure 1 S3

General Procedure 2 S14

NMR spectra S19

X-Ray structures: S46

Computational Studies: S48

Preliminary studies on the development of a catalytic enantioselective variant S65

References: S72

General Information

All experiments were performed under an atmosphere of nitrogen, using anhydrous solvents, unless stated otherwise. THF was distilled from sodium / benzophenone. Dichloromethane was distilled from CaH_2. Triethylamine was distilled from CaH_2.

^{1}H NMR and ^{13}C NMR were recorded using 300, 400 and 500 MHz spectrometers, with chemical shift values being reported in ppm relative to residual chloroform (δ_H = 7.27 or δ_C = 77.2) as internal standards. All coupling constants (*J*) are reported in Hertz (Hz). Mass spectra were obtained using positive and negative electrospray (ES±) or gas chromatography (GC) methodology. Infra-red spectra were recorded as evaporated films or neat using a FT/IR spectrometer. Column chromatography was carried out using 35 – 70 m, 60A silica gel. Routine TLC analysis was carried out on aluminium sheets coated with silica gel 60 F254, 0.2 mm thickness and plates were viewed using a 254 mm ultraviolet lamp and dipped in aqueous potassium permanganate or *p*-anisaldehyde.

Reagents were either purchased directly from commercial suppliers or prepared according to literature procedures.

Allenes: (propa-1,2-dien-1-yl)benzene[1] (**1a**), buta-2,3-dien-1-ylcyclohexane[2] (**1c**), undeca-1,2-diene[3] (**1d**).

Imines: (*E*)-*N*-(4-methoxyphenyl)-1-phenylmethanimine[4] (**2b**), (*E*)-*N*,1-*bis*(4-methoxyphenyl)methanimine[4] (**2c**), (*E*)-*N*-(4-methoxyphenyl)-1-(4-(trifluoromethyl)phenyl)methanimine[4] (**2d**), (*E*)-1-(4-bromophenyl)-*N*-(4-methoxyphenyl)methanimine[5] (**2e**), (*E*)-1-(furan-2-yl)-*N*-(4-methoxyphenyl)methanimine[6] (**2f**), (*E*)-*N*-(4-methoxyphenyl)-1-(thiophen-3-yl)methanimine[7] (**2g**), (*E*)-*N*-(4-methoxyphenyl)-1-(naphthalen-1-yl)methanimine[8] (**2h**), (*E*)-1-mesityl-*N*-(4-methoxyphenyl)methanimine[9] (**2i**), (*E*)-*N*-(4-methoxyphenyl)-1-(*o*-tolyl)methanimine[10] (**2j**), (*E*)-*N*-(4-methoxyphenyl)-2-methylpropan-1-imine[11] (**2k**), (*E*)-*N*-(4-methoxyphenyl)-2,2-dimethylpropan-1-imine[12] (**2l**), (*E*)-*N*-benzyl-1-(*o*-tolyl)methanimine[4] (**2m**), (*E*)-*N*-benzyl-1-phenylmethanimine[13] (**2n**) and (*E*)-*N*-benzylidene-4-methylbenzenesulfonamide[14] (**2o**).

General Procedure 1 for the copper-catalysed borylation of allenes trapping with an imine

***rac-N*-((1*R*,2*S*)-1,2-Diphenyl-3-(4,4,5,5-tetramethyl-1,3,2-dioxaborolan-2-yl)but-3-en-1-yl)aniline (3a)**

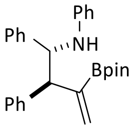

To a solution of IPrCuCl (6.3 mg, 0.013 mmol, 5 mol%) in THF (0.8 mL), was added *t*-BuOK (0.26 mL of a 1 M THF solution, 0.258 mmol, 1 equiv), and the reaction was stirred for 5 minutes at room temperature. B_2Pin_2 (72.1 mg, 0.284 mmol, 1.1 equiv) in THF (0.75 mL) was then added and the resulting mixture stirred for 30 min. A solution of (propa-1,2-dien-1-yl)benzene (45mg, 0.387 mmol, 1.5 equiv) and (*E*)-*N*,1-diphenylmethanimine (46.8 mg, 0.258 mmol, 1 equiv) in THF (1 mL) was then added dropwise at -78 °C, and the reaction allowed to warm to room temperature with stirring overnight. The mixture was then filtered through a silica plug, concentrated *in vacuo* and the crude product mixture was purified by chromatography (2% EtOAc in hexanes) to afford the title compound as an orange gum (96.6 mg, 0.227 mmol, 88%).

MS (ES^+) *m/z*: 426 ($M+H^+$). HRMS calcd for $C_{28}H_{33}NBO_2Na$: 426.2604. Found: 426.2614; ν_{max} (thin film/cm^{-1}): 2977, 1601, 1503, 1453, 1429, 1359, 1312, 1263, 1139; *Major diastereoisomer:* ^1H NMR (400 MHz, $CDCl_3$) δ ppm 1.09 (s, 6 H, 2 x CH_3), 1.14 (s, 6 H, 2 x CH_3), 3.73 (d, *J* = 9.2 Hz, 1 H, C*H*C=CH_2), 4.98 (d, *J* = 9.2 Hz, 1 H, CHN), 5.65 (d, *J* = 2.2 Hz, 1 H, C=CH_2), 5.98 (d, *J* = 2.8 Hz, 1 H, C=CH_2), 6.51 (d, *J* = 7.7 Hz, 1 H, ArCH), 6.60 (t, *J* = 7.3 Hz, 1 H, ArCH), 6.98 - 7.59 (m, 13 H, ArCH); *Major diastereoisomer:* ^{13}C NMR (101 MHz, $CDCl_3$) δ ppm 24.5 (CH_3), 24.6 (CH_3), 59.4 (*C*HC=CH_2), 60.6 (CHN), 83.7 (OC), 113.5 (ArCH), 116.9 (ArCH), 126.1 (ArCH), 126.7 (ArCH), 127.3 (ArCH), 127.8 (ArCH), 128.0 (ArCH), 128.3 (ArCH), 128.9 (ArCH), 132.2 (C=*C*H_2), 142.0 (ArC), 143.2 (ArC), 147.4 (ArC), (B*C*=CH_2 not observed); ^{11}B NMR (128 MHz, $CDCl_3$) δ ppm 8.3.

***rac-N*-((1*R*,2*R*)-2-Cyclohexyl-1-phenyl-3-(4,4,5,5-tetramethyl-1,3,2-dioxaborolan-2-yl)but-3-en-1-yl)aniline (3b)**

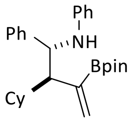

Prepared according to General Procedure 1, on a 0.258 mmol scale, column chromatography (2% EtOAc in Hexanes) afforded the title compound as an orange gum (97.9 mg, 0.227 mmol, 88%).

MS (ES^+) *m/z*: 432 ($M+H^+$). HRMS calcd for $C_{28}H_{38}NBO_2Na$: 453.2930. Found: 453.2924; ν_{max} (thin film/cm^{-1}): 3405, 3025, 2976, 2924, 2850, 1600, 1521, 1499, 1450, 1423, 1371, 1300, 1217, 1165, 1140, 1112, 1077, 1029; ^1H NMR (400 MHz, $CDCl_3$) δ ppm 0.82 - 1.16 (m, 2 H, CH_2), 1.19 - 1.35 (m,

3 H, CH_2), 1.39 (s, 6 H, 2 x CH_3), 1.41 (s, 6 H, 2 x CH_3), 1.67 - 1.85 (m, 4 H, CH_2), 1.87 - 2.03 (m, 2 H, CH_2 + CH), 2.34 (dd, *J* = 9.5, 4.9 Hz, 1 H, C*H*C=CH_2), 4.84 (d, *J* = 5.0 Hz, 1 H, CHN), 5.08 (d, *J* = 3.5 Hz, 1 H, C=CH_2), 5.80 (d, *J* = 3.5 Hz, 1 H, C=CH_2), 5.84 (br. s, 1 H, NH), 6.52 (dd, *J* = 8.6, 1.0 Hz, 2 H, ArCH), 6.61 - 6.67 (m, 1 H, ArCH), 7.10 - 7.17 (m, 2 H, ArCH), 7.19 - 7.29 (m, 3 H, ArCH), 7.29 - 7.37 (m, 2 H, ArCH); ^{13}C NMR (101 MHz, $CDCl_3$) δ ppm 24.4 (CH_3), 24.9 (CH_3), 26.3 (CH_2), 26.4 (CH_2), 26.5 (CH_2), 31.3 (CH_2), 32.4 (CH_2), 36.8 (CH), 57.4 (CHN), 61.4 (*C*HC=CH_2), 83.7 (OC), 112.5 (ArCH), 115.8 (ArCH), 126.1 (ArCH), 127.1 (ArCH), 127.9 (ArCH), 128.9 (ArCH), 135.0 (C=*C*H_2), 143.6 (ArC), 148.1 (ArC), (B*C*=CH_2 not observed); ^{11}B NMR (128 MHz, $CDCl_3$) δ ppm 7.8.

***rac-N*-((1*R*,2*R*)-2-(Cyclohexylmethyl)-1-phenyl-3-(4,4,5,5-tetramethyl-1,3,2-dioxaborolan-2-yl)but-3-en-1-yl)aniline (3c)**

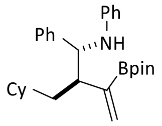

Prepared according to General Procedure 1, on a 0.258 mmol scale, column chromatography (3% EtOAc in Hexanes) afforded the title compound as a yellow solid (115 mg, 0.258 mmol, 99%).
Mp: 102-104 °C; MS (ES$^+$) *m/z*: 445 (M). HRMS calcd for $C_{29}H_{41}NBO_2Na$: 446.3230. Found: 446.3231; ν_{max} (thin film/cm^{-1}): 3404, 2976, 2921, 2850, 1601, 1501, 1449, 1420, 1370, 1310, 1262, 1212, 1167, 1141, 1077, 1029; ^{1}H NMR (400 MHz, $CDCl_3$) δ ppm 0.49 - 0.68 (m, 1 H, CH_2), 0.88 (d, *J* = 3.0 Hz, 1 H, CH_2), 1.01 - 1.21 (m, 4 H, CH_2), 1.24 (s, 6 H, 2 x CH_3), 1.25 (s, 6 H, 2 x CH_3), 1.50 - 1.79 (m, 7 H, CH_2 + CH), 2.56 - 2.67 (m, 1 H, C*H*C=CH_2), 4.35 (d, *J* = 7.1 Hz, 1 H, CHN), 5.07 (br. s, 1 H, NH), 5.42 (br. s, 1 H, C=CH_2), 5.88 (d, *J* = 3.5 Hz, 1 H, C=CH_2), 6.46 (d, *J* = 8.3 Hz, 2 H, ArCH), 6.57 (q, *J* = 7.1 Hz, 1 H, ArCH), 6.98 - 7.08 (m, 2 H, ArCH), 7.14 - 7.21 (m, 1 H, ArCH), 7.24 - 7.34 (m, 4 H, ArCH); ^{13}C NMR (101 MHz, $CDCl_3$) δ ppm 24.5 (CH_3), 24.8 (CH_3), 26.0 (CH_2), 26.4 (CH_2), 26.6 (CH_2), 31.7 (CH_2), 34.5 (CH_2), 35.0 (CH_2), 39.0 (CH), 51.6 (*C*HC=CH_2), 63.6 (CHN), 83.6 (OC), 113.1 (ArCH), 126.5 (ArCH), 127.3 (ArCH), 127.4 (ArCH), 128.0 (ArCH), 128.8 (ArCH), 133.2 (C=*C*H_2), 143.5 (ArC), 148.1 (ArC), (B*C*=CH_2 not observed); ^{11}B NMR (128 MHz, $CDCl_3$) δ ppm 4.7.

***rac-N*-((1*R*,2*R*)-1-Phenyl-2-(1-(4,4,5,5-tetramethyl-1,3,2-dioxaborolan-2-yl)vinyl)decyl)aniline (3d)**

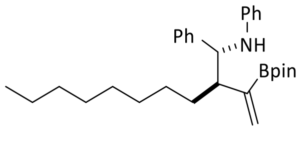

Prepared according to General Procedure 1, on a 0.258 mmol scale, column chromatography (3% EtOAc in Hexanes) afforded the title compound as a yellow gum (119 mg, 0.258 mmol, 99%).

MS (ES$^+$) m/z: 462 (M+H$^+$). HRMS calcd for $C_{30}H_{45}NBO_2Na$: 462.3543. Found: 462.3536; ν_{max} (thin film/cm^{-1}): 3405, 2924, 2854, 1601, 1502, 1452, 1419, 1370, 1309, 1262, 1213, 1141, 1111, 1077, 1029; ^{1}H NMR (500 MHz, $CDCl_3$) δ ppm 0.87 (t, J = 7.1 Hz, 3 H, CH_3), 0.96 - 1.77 (m, 14 H, CH_2), 1.23 (s, 12 H, 4 x CH_3), 2.38 - 2.50 (m, 1 H, C*H*C=CH_2), 4.41 (d, J = 7.6 Hz, 1 H, CHN), 4.97 (br. s, 1 H, NH), 5.41 - 5.49 (m, 1 H, C=CH_2), 5.90 (d, J = 3.5 Hz, 1 H, C=CH_2), 6.41 - 6.50 (m, 2 H, ArCH), 6.53 - 6.60 (m, 1 H, ArCH), 6.99 - 7.07 (m, 2 H, ArCH), 7.15 - 7.22 (m, 1 H, ArCH), 7.24 - 7.35 (m, 4 H, ArCH); ^{13}C NMR (126 MHz, $CDCl_3$) δ ppm 14.1 (CH_3), 22.6 (CH_2), 24.6 (CH_3), 24.7 (CH_3), 27.8 (CH_2), 29.2 (CH_2), 29.4 (2 x CH_2), 31.2 (CH_2), 31.8 (CH_2), 54.8 (*C*HC=CH_2), 61.7 (CN), 83.5 (OC), 112.9 (ArCH), 116.3 (ArCH), 126.5 (ArCH), 127.4 (ArCH), 128.1 (ArCH), 128.8 (ArCH), 133.0 (C=*C*H_2), 143.8 (ArC), 148.0 (ArC), (B*C*=CH_2 not observed); ^{11}B NMR (128 MHz, $CDCl_3$) δ ppm 5.1.

***rac-N*-((1*R*,2*R*)-2-Cyclohexyl-1-phenyl-3-(4,4,5,5-tetramethyl-1,3,2-dioxaborolan-2-yl)but-3-en-1-yl)-4-methoxyaniline (3e)**

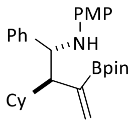

Prepared according to General Procedure 1, on a 0.258 mmol scale, column chromatography (3% EtOAc in Hexanes) afforded the title compound as an orange gum (105 mg, 0.227 mmol, 88%).
MS (ES$^+$) m/z: 462 (M+H$^+$). HRMS calcd for $C_{29}H_{41}NBO_3$: 462.3179. Found: 462.3162; ν_{max} (thin film/cm^{-1}): 3407, 2976, 2925, 2850, 1510, 1450, 1421, 1389, 1371, 1300, 1233, 1218, 1140, 1115, 1069, 1040; ^{1}H NMR (500 MHz, $CDCl_3$) δ ppm 0.75 - 0.86 (m, 1 H, CH_2), 1.04 (qd, J = 11.7, 2.5 Hz, 1 H, CH_2), 1.09 - 1.27 (m, 3 H, CH_2), 1.29 (s, 6 H, 2 x CH_3), 1.32 (s, 6 H, 2 x CH_3), 1.60 - 1.76 (m, 4 H, CH_2), 1.77 - 1.91 (m, 2 H, CH_2 + CH), 2.26 (dd, J = 9.0, 5.2 Hz, 1 H, C*H*C=CH_2), 3.69 (s, 3 H, OCH_3), 4.69 (d, J = 5.0 Hz, 1 H, CHN), 5.04 (d, J = 3.5 Hz, 1 H, C=CH_2), 5.33 (br. s., 1 H, NH), 5.74 (d, J = 3.8 Hz, 1 H, C=CH_2), 6.39 (d, J = 8.8 Hz, 2 H, ArCH), 6.67 (d, J = 8.8 Hz, 2 H, ArCH), 7.14 (tt, J = 6.9, 1.3 Hz, 1 H, ArCH), 7.18 - 7.22 (m, 2 H, ArCH), 7.22 - 7.28 (m, 2 H, ArCH); ^{13}C NMR (126 MHz, $CDCl_3$) δ ppm 24.4 (CH_3), 24.9 (CH_3), 26.3 (CH_2), 26.4 (CH_2), 26.6 (CH_2), 31.4 (CH_2), 32.2 (CH_2), 36.9 (CH), 55.8 (CHN), 58.1 (OCH_3), 61.3 (*C*HC=CH_2), 83.6 (OC), 113.3 (ArCH), 114.7 (ArCH), 126.1 (ArCH), 127.2 (ArCH), 127.9 (ArCH), 134.6 (C=*C*H_2), 142.6 (ArC), 144.0 (ArC), 150.9 (ArC), (B*C*=CH_2 not observed); ^{11}B NMR (128 MHz, $CDCl_3$) δ ppm 1.5.

***rac-N*-((1*R*,2*R*)-2-Cyclohexyl-1-(4-methoxyphenyl)-3-(4,4,5,5-tetramethyl-1,3,2-dioxaborolan-2-yl)but-3-en-1-yl)-4-methoxyaniline (3f)**

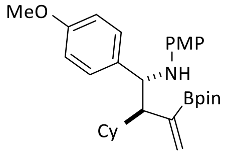

Prepared according to General Procedure 1, on a 0.258 mmol scale, column chromatography (4% EtOAc in Hexanes) afforded the title compound as an orange gum (123 mg, 0.250 mmol, 97%).

MS (ES$^+$) *m/z*: 492 (M+H$^+$). HRMS calcd for $C_{30}H_{43}NBO_4$: 492.3285. Found: 492.3305; ν_{max} (thin film/cm^{-1}): 3409, 2927, 2850, 1610, 1511, 1421, 1371, 1301, 1243, 1170, 1142; ^{1}H NMR (400 MHz, $CDCl_3$) δ ppm 0.68 - 0.92 (m, 1 H, CH_2), 0.93 - 1.08 (m, 1 H, CH_2), 1.09 - 1.24 (m, 4 H, CH_2), 1.27 (s, 6 H, 2 x CH_3), 1.29 (s, 6 H, 2 x CH_3), 1.53 - 1.92 (m, 5 H, CH_2 + CH), 2.19 (dd, *J* = 8.7, 5.4 Hz, 1 H, C*H*C=CH_2), 3.68 (s, 3 H, OCH_3), 3.77 (s, 3 H, OCH_3), 4.62 (d, *J* = 5.3 Hz, 1 H, CHN), 5.06 (d, *J* = 3.5 Hz, 1 H, C=CH_2), 5.75 (d, *J* = 3.8 Hz, 1 H, C=CH_2), 6.37 (d, *J* = 8.8 Hz, 2 H, ArCH), 6.63 - 6.67 (m, 2 H, ArCH), 6.78 (d, *J* = 8.5 Hz, 2 H, ArCH), 7.09 (d, *J* = 8.5 Hz, 2 H, ArCH); ^{13}C NMR (101 MHz, $CDCl_3$) δ ppm 24.4 (CH_3), 24.9 (CH_3), 26.3 (CH_2), 26.4 (CH_2), 26.6 (CH_2), 31.4 (CH_2), 32.1 (CH_2), 36.8 (CH), 55.1 (OCH_3), 55.8 (OCH_3), 57.5 (CHN), 61.4 (*C*HC=CH_2), 83.6 (OC), 113.3 (ArCH), 113.4 (ArCH), 114.7 (ArCH), 128.1 (ArCH), 134.6 (C=*C*H_2), 135.9 (ArC), 142.7 (ArC), 150.8 (ArC), 157.8 (ArC), (B*C*=CH_2 not observed); ^{11}B NMR (128 MHz, $CDCl_3$) δ ppm 4.4.

rac-N-((1*R*,2*R*)-2-Cyclohexyl-3-(4,4,5,5-tetramethyl-1,3,2-dioxaborolan-2-yl)-1-(4-(trifluoromethyl)phenyl)but-3-en-1-yl)-4-methoxyaniline (3g)

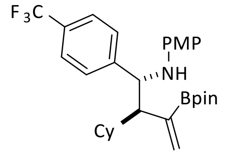

Prepared according to General Procedure 1, on a 0.258 mmol scale, column chromatography (3% EtOAc in Hexanes) afforded the title compound as a brown gum (103 mg, 0.194 mmol, 75%).

MS (ES$^+$) *m/z*: 530 (M+H$^+$). HRMS calcd for $C_{30}H_{40}NBO_2F_3$: 530.3062. Found: 530.3059; ν_{max} (thin film/cm^{-1}): 3405, 2977, 2927, 2851, 1617, 1511, 1420, 1371, 1334, 1234, 1066, 1040, 1016; ^{1}H NMR (400 MHz, $CDCl_3$) δ ppm 0.73 - 1.09 (m, 2 H, CH_2), 1.12 - 1.26 (m, 3 H, CH_2), 1.30 (s, 6 H, 2 x CH_3), 1.33 (s, 6 H, 2 x CH_3), 1.61 - 1.77 (m, 4 H, CH_2 + CH), 1.87 (t, *J* = 11.9 Hz, 2 H, CH_2), 2.25 (dd, *J* = 9.3, 4.6 Hz, 1 H, C*H*C=CH_2), 3.69 (s, 3 H, OCH_3), 4.73 (d, *J* = 4.5 Hz, 1 H, CHN), 5.00 (d, *J* = 3.2 Hz, 1 H, C=CH_2), 5.73 (d, *J* = 3.4 Hz, 1 H, C=CH_2), 6.36 (d, *J* = 8.8 Hz, 2 H, ArCH), 6.69 (d, *J* = 8.8 Hz, 2 H, ArCH), 7.25 - 7.34 (m, 2 H, ArCH), 7.51 (d, *J* = 8.1 Hz, 2 H, ArCH); ^{13}C NMR (101 MHz, $CDCl_3$) δ ppm 24.4 (CH_3), 24.9 (CH_3), 26.3 (CH_2), 26.4 (CH_2), 26.5 (CH_2), 31.2 (CH_2), 32.4 (CH_2), 36.8 (CH), 55.8 (CHN), 57.9 (OCH_3), 61.1 (*C*HC=CH_2), 83.8 (OC), 114.8 (ArCH), 124.4 (q, *J* = 272.0 Hz, CF_3), 124.9

(ArCH), 124.9 (q, *J* = 3.7 Hz, ArCH), 127.5 (ArCH), 128.4 (q, *J* = 31.7 Hz, *C*CF_3), 135.1 (ArCH), 142.0 (ArC), 148.5 (ArC), 151.2 (ArC), (B*C*=CH_2 not observed).

***rac*-*N*-((1*R*,2*R*)-1-(4-Bromophenyl)-2-cyclohexyl-3-(4,4,5,5-tetramethyl-1,3,2-dioxaborolan-2-yl)but-3-en-1-yl)-4-methoxyaniline (3h)**

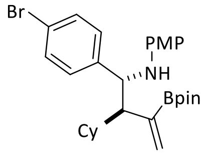

Prepared according to General Procedure 1, on a 0.258 mmol scale, column chromatography (3% EtOAc in Hexanes) afforded the title compound as an orange gum (111 mg, 0.206 mmol, 80%).
MS (ES^+) *m/z*: 542 ($M+H^+$). HRMS calcd for $C_{29}H_{40}NBO_3Br$: 540.2285. Found: 540.2273; ν_{max} (thin film/cm^{-1}): 3405, 2976, 2926, 2850, 1515, 1484, 1421, 1371, 1302, 1234, 1167, 1141, 1071, 1040, 1009; ^1H NMR (500 MHz, $CDCl_3$) δ ppm 1.07 - 1.23 (m, 5 H, CH_2), 1.28 (s, 6 H, 2 x CH_3), 1.31 (s, 6 H, 2 x CH_3), 1.59 - 1.73 (m, 4 H, CH_2), 1.76 - 1.89 (m, 2 H, CH + CH $_2$), 2.19 (dd, *J* = 9.1, 5.0 Hz, 1 H, C*H*C=CH_2), 3.69 (s, 3 H, OCH_3), 4.62 (d, *J* = 5.0 Hz, 1 H, CHN), 5.03 (d, *J* = 3.2 Hz, 1 H, C=CH_2), 5.33 (br. s, 1 H, NH), 5.74 (d, *J* = 3.5 Hz, 1 H, C=CH_2), 6.34 (d, *J* = 8.8 Hz, 2 H, ArCH), 6.66 (d, *J* = 8.8 Hz, 2 H, ArCH), 7.06 (d, *J* = 8.2 Hz, 2 H, ArCH), 7.35 (d, *J* = 8.5 Hz, 2 H, ArCH); ^{13}C NMR (126 MHz, $CDCl_3$) δ ppm 24.4 (CH_3), 24.9 (CH_3), 26.3 (CH_2), 26.4 (CH_2), 26.5 (CH_2), 31.3 (CH_2), 32.3 (CH_2), 36.8 (CH), 55.8 (OCH_3), 57.7 (CHN), 61.2 (*C*HC=CH_2), 83.7 (OC), 113.4 (ArCH), 114.8 (ArCH), 119.7 (ArC), 129.0 (ArCH), 131.0 (ArCH), 135.0 (C=*C*H_2), 142.2 (ArC), 143.2 (ArC), 151.1 (ArC), (B*C*=CH_2 not observed); ^{11}B NMR (128 MHz, $CDCl_3$) δ ppm 9.7.

***rac*-*N*-((1*R*,2*R*)-2-Cyclohexyl-1-(furan-2-yl)-3-(4,4,5,5-tetramethyl-1,3,2-dioxaborolan-2-yl)but-3-en-1-yl)-4-methoxyaniline (3i)**

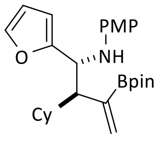

Prepared according to General Procedure 1, on a 0.258 mmol scale, column chromatography (3% EtOAc in Hexanes) afforded the title compound as a brown gum (81.3 mg, 0.181 mmol, 70%).
MS (ES^+) *m/z*: 452 ($M+H^+$). HRMS calcd for $C_{27}H_{39}NBO_4$: 452.2972. Found: 452.2976; ν_{max} (thin film/cm^{-1}): 3404, 2976, 2926, 2850, 1516, 1448, 1422, 1371, 1301, 1233, 1142, 1339; ^1H NMR (400 MHz, $CDCl_3$) δ ppm 0.73 - 1.22 (m, 5 H, CH_2), 1.25 (s, 6 H, 2 x CH_3), 1.26 (s, 6 H, 2 x CH_3), 1.54 - 1.85 (m, 6 H, CH_2 + CH), 2.23 (dd, *J* = 7.8, 6.3 Hz, 1 H, C*H*C=CH_2), 3.71 (s, 3 H, OCH_3), 4.65 (d, *J* = 6.1 Hz, 1 H, CHN), 5.37 (d, *J* = 3.5 Hz, 1 H, C=CH_2), 5.88 (d, *J* = 3.5 Hz, 1 H, C=CH_2), 6.23 (dd, *J* = 1.8, 0.8 Hz, 1

H, ArC*H*), 6.43 - 6.53 (m, 2 H, ArCH), 6.66 - 6.73 (m, 2 H, ArCH), 7.14 - 7.19 (m, 1 H, ArCH), 7.28 - 7.34 (m, 1 H, ArCH); ^{13}C NMR (101 MHz, $CDCl_3$) δ ppm 24.4 (CH_3), 24.9 (CH_3), 26.4 (CH_2), 26.47 (CH_2), 26.5 (CH_2), 31.5 (CH_2), 31.7 (CH_2), 36.9 (CH), 50.6 (CHN), 55.8 (OCH_3), 59.6 (*C*HC=CH_2), 83.5 (OC), 109.6 (ArCH), 113.6 (ArCH), 114.65 (ArCH), 114.7 (ArCH), 128.3 (ArCH), 134.2 (C=*C*H_2), 140.1 (ArC), 142.6 (ArC), 151.2 (ArC), (B*C*=CH_2 not observed); ^{11}B NMR (128 MHz, $CDCl_3$) δ ppm 30.6.

***rac-N*-((1*R*,2*R*)-2-Cyclohexyl-3-(4,4,5,5-tetramethyl-1,3,2-dioxaborolan-2-yl)-1-(thiophen-2-yl)but-3-en-1-yl)-4-methoxyaniline (3j)**

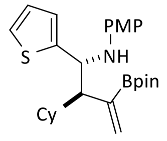

Prepared according to General Procedure 1, on a 0.258 mmol scale, column chromatography (3% EtOAc in Hexanes) afforded the title compound as an orange gum (95.1 mg, 0.204 mmol, 79%).

MS (ES$^+$) *m/z*: 468 (M+H$^+$). HRMS calcd for $C_{27}H_{30}NBO_3S$: 468.2744. Found: 468.2728; ν_{max} (thin film/cm^{-1}): 3401, 2976, 2926, 2850, 1511, 1422, 1371, 1302, 1234, 1169, 1141, 1040; ^{1}H NMR (400 MHz, $CDCl_3$) δ ppm 0.73 - 1.17 (m, 5 H, CH_2), 1.26 (s, 6 H, 2 x CH_3), 1.27 (s, 6 H, 2 x CH_3), 1.58 - 1.82 (m, 6 H, CH_2 + CH), 2.30 (t, *J* = 7.1 Hz, 1 H, C*H*C=CH_2), 3.70 (s, 3 H, OCH_3), 4.96 (d, *J* = 6.2 Hz, 1 H, CHN), 5.14 (br. s., 1 H, NH), 5.33 (d, *J* = 3.4 Hz, 1 H, C=CH_2), 5.83 (d, *J* = 3.7 Hz, 1 H, C=CH_2), 6.45 - 6.54 (m, 2 H, ArCH), 6.66 - 6.73 (m, 2 H, ArCH), 6.81 - 6.86 (m, 1 H, ArCH), 6.89 (dd, *J* = 4.9, 3.5 Hz, 1 H, ArCH), 7.03 - 7.11 (m, 1 H, ArCH); ^{13}C NMR (101 MHz, $CDCl_3$) δ ppm 24.4 (CH_3), 24.9 (CH_3), 26.4 (CH_2), 26.5 (CH_2), 26.5 (CH_2), 31.5 (CH_2), 31.6 (CH_2), 37.1 (CH), 54.8 (CHN), 55.8 (OCH_3), 61.8 (*C*HC=CH_2), 83.6 (OC), 113.6 (ArCH), 114.7 (ArCH), 123.0 (ArCH), 123.3 (ArCH), 126.5 (ArCH), 134.6 (C=*C*H_2), 142.3 (ArC), 150.4 (ArC), 151.3 (ArC), (B*C*=CH_2 not observed); ^{11}B NMR (128 MHz, $CDCl_3$) δ ppm 8.7.

***rac-N*-((1*R*,2*R*)-2-Cyclohexyl-1-(naphthalen-1-yl)-3-(4,4,5,5-tetramethyl-1,3,2-dioxaborolan-2-yl)but-3-en-1-yl)-4-methoxyaniline (3k)**

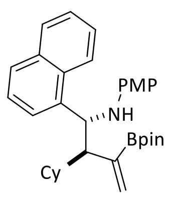

Prepared according to General Procedure 1, on a 0.258 mmol scale, column chromatography (3% EtOAc in Hexanes) afforded the title compound as a yellow gum (98.8 mg, 0.191 mmol, 74%).

MS (ES$^+$) *m/z*: 512 (M+H$^+$). HRMS calcd for $C_{33}H_{43}NBO_3$: 512.3336. Found: 512.3332; ν_{max} (thin film/cm^{-1}): 3408, 2976, 2926, 2850, 1598, 1511, 1422, 1371, 1302, 1234, 1173, 1142, 1040; ^{1}H

NMR (400 MHz, $CDCl_3$) δ ppm 0.71 - 0.98 (m, 2 H, CH_2), 1.03 - 1.29 (m, 3 H, CH_2), 1.32 (s, 6 H, 2 x CH_3), 1.37 (s, 6 H, 2 x CH_3), 1.53 - 1.87 (m, 4 H, CH_2), 1.99 - 2.13 (m, 1 H, CH_2), 2.18 - 2.28 (m, 1 H, CH), 2.49 - 2.59 (m, 1 H, C*H*C=CH_2), 3.65 (s, 3 H, OCH_3), 4.54 (br. s., 1 H, C=CH_2), 5.52 (d, *J* = 3.0 Hz, 1 H, C=CH_2), 5.55 (br. s., 1 H, CHN), 5.77 (br. s., 1 H, NH), 6.34 (d, *J* = 7.8 Hz, 2 H, ArCH), 6.58 - 6.66 (m, 2 H, ArCH), 7.21 - 7.38 (m, 2 H, ArCH), 7.45 - 7.61 (m, 2 H, ArCH), 7.62 - 7.70 (m, 1 H, ArCH), 7.88 (d, *J* = 7.1 Hz, 1 H, ArCH), 8.20 (d, *J* = 8.3 Hz, 1 H, ArCH); ^{13}C NMR (101 MHz, $CDCl_3$) δ ppm 24.4 (CH_3), 25.0 (CH_3), 26.5 (CH_2), 26.6 (CH_2), 31.1 (CH_2), 33.2 (CH_2), 37.1 (CH), 53.9 (CHN), 55.8 (OCH_3), 58.7 (*C*HC=CH_2), 83.8 (OC), 113.1 (ArCH), 114.8 (ArCH), 122.4 (ArCH), 124.9 (ArCH), 125.2 (ArCH), 125.4 (ArCH), 125.6 (ArCH), 126.7 (ArCH), 129.2 (ArC), 130.9 (ArC), 134.0 (ArC), 134.7 (C=*C*H_2), 138.0 (ArC), 142.3 (ArC), (B*C*=CH_2 not observed); ^{11}B NMR (128 MHz, $CDCl_3$) δ ppm 5.1.

***rac-N*-((1*R*,2*R*)-2-Cyclohexyl-3-(4,4,5,5-tetramethyl-1,3,2-dioxaborolan-2-yl)-1-(*o*-tolyl)but-3-en-1-yl)-4-methoxyaniline (3m)**

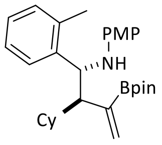

Prepared according to General Procedure 1, on a 0.258 mmol scale, column chromatography (3% EtOAc in Hexanes) afforded the title compound as a yellow gum (97.6 mg, 0.206 mmol, 80%).

MS (ES$^+$) *m/z*: 476 (M+H$^+$). HRMS calcd for $C_{30}H_{42}NBO_3$: 476.3341. Found: 476.3357; ν_{max} (thin film/cm^{-1}): 3408, 2976, 2927, 2850, 1525, 1462, 1447, 1421, 1371, 1300, 1277, 1233, 1180, 1165, 1142, 1041; ^{1}H NMR (400 MHz, $CDCl_3$) δ ppm 0.71 - 1.23 (m, 5 H, CH_2), 1.31 (s, 6 H, 2 x CH_3), 1.36 (s, 6 H, 2 x CH_3), 1.55 - 1.79 (m, 4 H, CH_2), 1.88 - 2.08 (m, 2 H, CH_2 + CH), 2.20 (d, *J* = 7.6 Hz, 1 H, C*H*C=CH_2), 2.46 (br. s., 3 H, Ar-CH_3), 3.68 (s, 3 H, OCH_3), 4.88 (br. s., 2 H, CHN + C=CH_2), 5.56 (br. s., 1 H, NH), 5.70 (br. s., 1 H, C=CH_2), 6.30 (d, *J* = 8.1 Hz, 2 H, ArCH), 6.63 - 6.69 (m, 2 H, ArCH), 6.94 - 7.15 (m, 4 H, ArCH); ^{13}C NMR (101 MHz, $CDCl_3$) δ ppm 19.1 (Ar-*C*H_3), 24.4 (CH_3), 25.0 (CH_3), 26.4 (CH_2), 26.5 (CH_2), 26.5 (CH_2), 31.0 (CH_2), 33.0 (CH_2), 36.9 (CH), 54.4 (CHN), 55.8 (OCH_3), 58.2 (*C*HC=CH_2), 83.7 (OC), 112.9 (ArCH), 114.8 (ArCH), 125.4 (ArCH), 125.8 (ArCH), 127.8 (ArCH), 130.1 (ArCH), 134.2 (ArC), 134.8 (C=*C*H_2), 140.9 (ArC), 142.5 (ArC), 150.7 (Ar*C*OCH_3), (B*C*=CH_2 not observed); ^{11}B NMR (128 MHz, $CDCl_3$) δ ppm 8.0.

***rac-N*-((3*S*,4*R*)-4-Cyclohexyl-2-methyl-5-(4,4,5,5-tetramethyl-1,3,2-dioxaborolan-2-yl)hex-5-en-3-yl)-4-methoxyaniline (3n)**

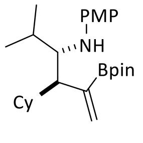

Prepared according to General Procedure 1, on a 0.258 mmol scale, column chromatography (3% EtOAc in Hexanes) afforded the title compound as a brown gum (40.4 mg, 0.096 mmol, 37%).

MS (ES$^+$) *m/z*: 428 (M+H$^+$). HRMS calcd for $C_{26}H_{43}NBO_3$: 428.3336. Found: 428.3329; ν_{max} (thin film/cm^{-1}): 3402, 2923, 2850, 1617, 1510, 1448, 1371, 1301, 1232, 1166, 1142, 1109, 1041; ^{1}H NMR (400 MHz, $CDCl_3$) δ ppm 0.92 (t, *J* = 6.1 Hz, 6 H, 2 x CH_3), 1.02 - 1.15 (m, 3 H, CH_2), 1.26 (s, 6 H, 2 x CH_3), 1.27 - 1.29 (m, 6 H, 2 x CH_3), 1.49 - 1.76 (m, 9 H, $(CH_3)_2CH$ + CH_2 + CH), 2.26 (dd, *J* = 5.0 Hz, 1 H, C*H*C=CH_2), 3.27 (dd, *J* = 5.4, 3.7 Hz, 1 H, CHN), 3.74 (s, 3 H, OCH_3), 4.43 (br. s, 1 H, NH), 5.62 (d, *J* = 2.8 Hz, 1 H, C=CH_2), 5.96 (d, *J* = 2.8 Hz, 1 H, C=CH_2), 6.53 (d, *J* = 8.3 Hz, 2 H, ArCH), 6.72 (d, *J* = 8.3 Hz, 2 H, ArCH); ^{13}C NMR (101 MHz, $CDCl_3$) δ ppm 19.8 (CH($C$$H_3$)$_2$), 20.5 (CH($C$$H_3$)$_2$), 24.4 ($CH_3$), 24.6 ($C$H($CH_3$)$_2$), 24.9 ($CH_3$), 26.2 ($CH_2$), 26.5 ($CH_2$), 26.6 ($CH_2$), 31.9 ($CH_2$), 32.1 ($CH_2$), 37.2 (CH), 55.9 (CHN + OCH_3), 59.2 (*C*HC=CH_2), 83.3 (OC), 113.1 (ArCH), 114.7 (ArCH), 132.8 (C=*C*H_2), 144.9 (ArC), 150.3 (ArC), (B*C*=CH_2 not observed); ^{11}B NMR (128 MHz, $CDCl_3$) δ ppm 9.1.

rac-_N_-((3_R_,4_R_)-4-Cyclohexyl-2,2-dimethyl-5-(4,4,5,5-tetramethyl-1,3,2-dioxaborolan-2-yl)hex-5-en-3-yl)-4-methoxyaniline (3o)

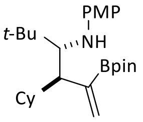

Prepared according to General Procedure 1, on a 0.258 mmol scale, column chromatography (2% EtOAc in Hexanes) afforded the title compound as a brown gum (6.3 mg, 0.016 mmol, 6%).

MS (ES$^+$) *m/z*: 443 (M+H$^+$). HRMS calcd for $C_{27}H_{45}NBO_3$: 442.3493. Found: 442.3481; ν_{max} (thin film/cm^{-1}): 3394, 2924, 2852, 1509, 1465, 1370, 1302, 1233, 1142, 1043; ^{1}H NMR (400 MHz, $CDCl_3$) δ ppm 0.92 (s, 9 H, 3 x CH_3), 1.03 - 1.12 (m, 3 H, CH_2), 1.24 (s, 6 H, 2 x CH_3), 1.25 (s, 6 H, 2 x CH_3), 1.48 - 1.65 (m, 7 H, CH_2), 1.83 - 1.92 (m, 1 H, CH), 2.36 (d, *J* = 9.4 Hz, 1 H, C*H*C=CH_2), 3.30 - 3.39 (m, 1 H, CHN), 3.75 (s, 3 H, OCH_3), 4.64 (br. s, 1 H, NH), 5.56 (d, *J* = 3.3 Hz, 1 H, C=CH_2), 5.93 (d, *J* = 3.5 Hz, 1 H, C=CH_2), 6.53 - 6.64 (m, 2 H, ArCH), 6.73 (d, *J* = 8.6 Hz, 2 H, ArCH); ^{13}C NMR (101 MHz, $CDCl_3$) δ ppm 24.6 (CH_3), 25.0 (CH_3), 26.2 (CH_2), 26.4 (CH_2), 26.6 (CH_2), 27.9 (C($C$$H_3$)$_3$), 29.7 ($C$($CH_3$)$_3$), 32.4 ($CH_2$), 32.5 ($CH_2$), 38.0 (CH), 55.3 (*C*HC=CH_2), 56.0 (OCH_3), 62.8 (CHN), 83.3 (OC), 113.8 (ArCH), 114.6 (ArCH), 131.9 (C=*C*H_2), 145.3 (ArC), 150.6 (ArC), (B*C*=CH_2 not observed).

4-Methoxy-_N_-(1-(4-methoxyphenyl)-2,2-dimethyl-3-(4,4,5,5-tetramethyl-1,3,2-dioxaborolan-2-yl)but-3-en-1-yl)aniline (3p)

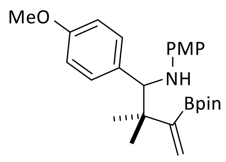

Prepared according to General Procedure 1, on a 0.258 mmol scale, column chromatography (6% EtOAc in Hexanes) afforded the title compound as a brown solid (97 mg, 0.222 mmol, 86%).

Mp: 71-73 °C; MS (ES$^+$) *m/z*: 438 (M+H$^+$). HRMS calcd for $C_{26}H_{36}NBO_4Na$: 460.2635. Found: 460.2652; ν_{max} (thin film/cm^{-1}): 3409, 2975, 2933, 2833, 1609, 1583, 1509, 1464, 1442, 1411, 1353, 1301, 1236, 1216, 1168, 1144, 1120, 1109, 1036; ^{1}H NMR (400 MHz, $CDCl_3$) δ ppm 1.00 (s, 3 H, CH_3), 1.10 (s, 3 H, CH_3), 1.15 (s, 6 H, 2 x CH_3), 1.17 (s, 6 H, 2 x CH_3), 3.66 (s, 3 H, OCH_3), 3.79 (s, 3 H, OCH_3), 4.11 (br. s., 1 H, NH), 4.45 (s, 1 H, CHN), 5.67 (d, *J* = 2.2 Hz, 1 H, C=CH_2), 5.92 (d, *J* = 2.5 Hz, 1 H, C=CH_2), 6.33 - 6.39 (m, 2 H, ArCH), 6.57 - 6.63 (m, 2 H, ArCH), 6.80 - 6.85 (m, 2 H, ArCH), 7.25 - 7.30 (m, 2 H, ArCH); ^{13}C NMR (101 MHz, $CDCl_3$) δ ppm 21.1 (CH_3), 24.6 (CH_3), 24.7 (CH_3), 26.9 (CH_3), 43.3 (*C*C=CH_2), 55.1 (OCH_3), 55.8 (OCH_3), 65.2 (CHN), 83.4 (OC), 112.9 (ArCH), 114.4 (ArCH), 114.4 (ArCH), 127.7 (C=*C*H_2), 129.9 (ArCH), 133.1 (ArC), 142.6 (ArC), 151.5 (ArC), 158.3 (ArC), (B*C*=CH_2 not observed); ^{11}B NMR (128 MHz, $CDCl_3$) δ 4.5.

***N*-(1-(4-Bromophenyl)-2,2-dimethyl-3-(4,4,5,5-tetramethyl-1,3,2-dioxaborolan-2-yl)but-3-en-1-yl)-4-methoxyaniline (3q)**

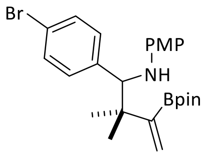

Prepared according to General Procedure 1, on a 0.258 mmol scale, column chromatography (4% EtOAc in Hexanes) afforded the title compound as a brown solid (103 mg, 0.211 mmol, 82%).

Mp: 53-55 °C; MS (ES$^+$) *m/z*: 486 (M+H$^+$). HRMS calcd for $C_{25}H_{34}NBO_3Br$: 486.1815. Found: 486.1811; ν_{max} (thin film/cm^{-1}): 3404, 2975, 2932, 2831, 1601, 1510, 1485, 1442, 1372, 1353, 1236, 1216, 1144, 1119, 1109; ^{1}H NMR (400 MHz, $CDCl_3$) δ ppm 0.99 (s, 3 H, CH_3), 1.11 (s, 3 H, CH_3), 1.15 (s, 6 H, CH_3), 1.17 (s, 6 H, CH_3), 3.66 (s, 3 H, OCH_3), 4.12 (br. s., 1 H, NH), 4.48 (s, 1 H, CHN), 5.68 (d, *J* = 2.3 Hz, 1 H, C=CH_2), 5.95 (d, *J* = 2.3 Hz, 1 H, C=CH_2), 6.29 - 6.35 (m, 2 H, ArCH), 6.58 - 6.63 (m, 2 H, ArCH), 7.23 - 7.29 (m, 2 H, ArCH), 7.38 - 7.44 (m, 2 H, ArCH); ^{13}C NMR (101 MHz, $CDCl_3$) δ ppm 21.1 (CH_3), 24.6 (CH_3), 24.7 (CH_3), 26.8 (CH_3), 43.0 (*C*C=CH_2), 55.7 (OCH_3), 65.1 (CHN), 83.5 (OC), 114.3 (ArCH), 114.5 (ArCH), 120.4 (ArC), 128.4 (C=*C*H_2), 130.6 (ArCH), 130.7 (ArCH), 140.4 (ArC), 142.1 (ArC), 151.6 (ArC), (*C*=CH_2 not observed); ^{11}B NMR (128 MHz, $CDCl_3$) δ 2.88.

***N*-(2,2-Dimethyl-1-phenyl-3-(4,4,5,5-tetramethyl-1,3,2-dioxaborolan-2-yl)but-3-en-1-yl)aniline (3r)**

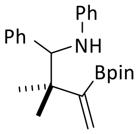

Prepared according to General Procedure 1, on a 0.258 mmol scale, preparative thin layer chromatography (CH_2Cl_2) afforded the title compound as a yellow solid (68.2 mg, 0.181 mmol, 70%).

Mp: 103-104 °C; MS (ES^+) *m/z*: 378 ($M+H^+$). HRMS calcd for $C_{24}H_{33}NBO_2$: 378.2604. Found: 378.2616; ν_{max} (thin film/cm^{-1}): 3407, 2976, 2930, 1601, 1503, 1453, 1411, 1389, 1372, 1353, 1301, 1275, 1215, 1144, 1119, 1078, 1029; ^1H NMR (500 MHz, $CDCl_3$) δ ppm 1.02 (s, 3 H, CH_3), 1.15 (s, 3 H, CH_3), 1.17 (s, 6 H, 2 x CH_3), 1.18 (s, 6 H, 2 x CH_3), 4.44 (br. s., 1 H, NH), 4.59 (d, *J* = 2.2 Hz, 1 H, CHN), 5.66 (d, *J* = 2.5 Hz, 1 H, $C=CH_2$), 5.94 (d, *J* = 2.5 Hz, 1 H, $C=CH_2$), 6.40 (dd, *J* = 8.7, 1.1 Hz, 2 H, ArCH), 6.54 (tt, *J* = 7.3, 0.9 Hz, 1 H, ArCH), 6.98 (dd, *J* = 8.5, 7.3 Hz, 2 H, ArCH), 7.19 - 7.24 (m, 1 H, ArCH), 7.28 (m, 2 H, ArCH), 7.34 - 7.39 (m, 2 H, ArCH); ^{13}C NMR (126 MHz, $CDCl_3$) δ ppm 21.4 (CH_3), 24.6 (CH_3), 24.7 (CH_3), 26.9 (CH_3), 43.1 (*C*C=CH_2), 65.0 (CHN), 83.5 (OC), 113.2 (ArCH), 116.6 (ArCH), 126.6 (ArCH), 127.5 (ArCH), 128.2 (C=*C*H_2), 128.7 (ArCH), 129.0 (ArCH), 141.1 (ArC), 148.1 (ArC), (B*C*=CH_2 not observed); ^{11}B NMR (128 MHz, $CDCl_3$) δ ppm 6.8.

***N*-(Furan-2-yl(1-(1-(4,4,5,5-tetramethyl-1,3,2-dioxaborolan-2-yl)vinyl)cyclohexyl)methyl)-4-methoxyaniline (3s)**

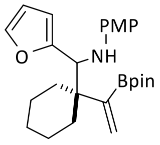

Prepared according to General Procedure 1, on a 0.258 mmol scale, column chromatography (4% EtOAc in Hexanes) afforded the title compound as a brown gum (94 mg, 0.222 mmol, 86%).

ν_{max} (thin film/cm^{-1}): 3410, 2975, 2931, 2855, 1631, 1511, 1453, 1371, 1244, 1142, 1119, 1036; ^1H NMR (400 MHz, $CDCl_3$) δ ppm 1.10 - 1.22 (m, 1 H, CH_2), 1.30 (s, 12 H, CH_3), 1.32 - 1.62 (m, 7 H, CH_2), 2.06 - 2.13 (m, 1 H, CH_2), 2.20 - 2.31 (m, 1 H, CH_2), 3.69 (s, 3 H, OCH_3), 4.06 (s, 1 H, CHN), 4.87 (br. s., 1 H, NH), 5.50 (d, *J* = 2.5 Hz, 1 H, $C=CH_2$), 6.08 (d, *J* = 2.3 Hz, 1 H, $C=CH_2$), 6.28 - 6.31 (m, 1 H , ArCH), 6.40 - 6.46 (m, 2 H, ArCH), 6.65 - 6.70 (m, 2 H, ArCH), 7.22 - 7.24 (m, 1 H, ArCH), 7.29 (t, *J* = 1.6 Hz, 1 H, ArCH); ^{13}C NMR (101 MHz, $CDCl_3$) δ ppm 22.0 (CH_2), 22.5 (CH_2), 24.65 (CH_3), 24.69 (CH_3), 26.6 (CH_2), 32.0 (CH_2), 34.2 (CH_2), 46.4 (*C*C=CH_2), 55.8 (OCH_3), 61.2 (CHN), 83.7 (OC), 111.6 (ArCH), 113.7 (ArCH), 114.7 (ArCH), 122.0 (ArCH), 125.6 (C=*C*H_2), 132.2 (ArCH), 141.0 (ArC), 141.7 (ArC), 151.2 (ArC), (B*C*=CH_2 not observed); ^{11}B NMR (128 MHz, $CDCl_3$) δ -2.94.

4-Methoxy-*N*-((1-(1-(4,4,5,5-tetramethyl-1,3,2-dioxaborolan-2-yl)vinyl)cyclohexyl)(thiophen-2-yl)methyl)aniline (3t)

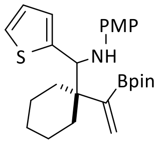

Prepared according to General Procedure 1, on a 0.258 mmol scale, column chromatography (4% EtOAc in Hexanes) afforded the title compound as a brown gum (100 mg, 0.227 mmol, 88%).

ν_{max} (thin film/cm^{-1}): 3405, 2975, 2930, 2955, 1616, 1510, 1451, 1371, 1296, 1275, 1246, 1194, 1141, 1118, 1036; ^{1}H NMR (400 MHz, $CDCl_3$) δ ppm 1.08 - 1.22 (m, 1 H, CH_2), 1.31 (s, 6 H, 2 x CH_3), 1.31 (s, 6 H, 2 x CH_3), 1.35 - 1.68 (m, 7 H, CH_2), 2.13 - 2.22 (m, 1 H, CH_2), 2.24 - 2.33 (m, 1 H, CH_2), 3.68 (s, 3 H, OCH_3), 4.35 (d, *J* = 4.3 Hz, 1 H, CHN), 5.22 (d, *J* = 4.0 Hz, 1 H, NH), 5.52 (d, *J* = 2.3 Hz, 1 H, C=CH_2), 6.11 (d, *J* = 2.3 Hz, 1 H, C=CH_2), 6.40 - 6.47 (m, 2 H, ArCH), 6.63 - 6.70 (m, 2 H, ArCH), 6.91 - 6.96 (m, 2 H, ArCH), 7.13 (dd, *J* = 4.8, 1.5 Hz, 1 H, ArCH); ^{13}C NMR (101 MHz, $CDCl_3$) δ ppm 22.1 (CH_2), 22.5 (CH_2), 24.69 (CH_3), 24.65 (CH_3), 26.5 (CH_2), 31.7 (CH_2), 34.4 (CH_2), 46.7 (*C*C=CH_2), 55.7 (OCH_3), 65.4 (CHN), 83.8 (OC), 113.7 (ArCH), 114.6 (ArCH), 123.7 (ArCH), 125.3 (ArCH), 126.0 (C=*C*H_2), 132.8 (ArCH), 142.7 (ArC), 147.3 (ArC), 151.4 (ArC), (B*C*=CH_2 not observed); ^{11}B NMR (128 MHz, $CDCl_3$) δ 6.4.

***rac*-4-Methoxy-*N*-((1*R*,2*R*)-2-(1-(4,4,5,5-tetramethyl-1,3,2-dioxaborolan-2-yl)vinyl)-1-(*o*-tolyl)decyl)aniline (3u)**

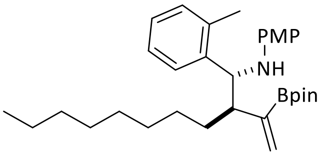

Prepared according to General Procedure 1, on a 0.258 mmol scale, column chromatography (4% EtOAc in Hexanes) afforded the title compound as an orange gum (119 mg, 0.235 mmol, 91%).

MS (ES$^+$) *m/z*: 505 (M+H$^+$). HRMS calcd for $C_{32}H_{49}NBO_3Na$: 506.3806. Found: 506.3802; ν_{max} (thin film/cm^{-1}): 3406, 2924, 2854, 1606, 1510, 1463, 1417, 1365, 1307, 1237, 1167, 1141, 1110, 1041; ^{1}H NMR (400 MHz, $CDCl_3$) δ ppm 0.83 - 0.90 (m, 3 H, CH_3), 1.01 - 1.37 (m, 13 H, CH_2), 1.23 (s, 6 H, 2 x CH_3), 1.24 (s, 6 H, 2 x CH_3), 1.71 (m, 1 H, CH_2), 2.35 - 2.45 (m, 1 H, C*H*C=CH_2), 2.50 (s, 3 H, Ar-CH_3), 3.67 (s, 3 H, OCH_3), 4.58 (br. s., 1 H, NH), 4.64 (d, *J* = 6.3 Hz, 1 H, CHN), 5.45 (br. s., 1 H, C=CH_2), 5.91 (d, *J* = 3.0 Hz, 1 H, C=CH_2), 6.33 (d, *J* = 6.8 Hz, 2 H, ArCH), 6.63 (d, *J* = 8.3 Hz, 2 H, ArCH), 7.04 - 7.15 (m, 3 H, ArCH), 7.22 - 7.33 (m, 1 H, ArCH); ^{13}C NMR (101 MHz, $CDCl_3$) δ ppm 14.1 (CH_3), 19.4 (Ar-CH_3), 22.6 (CH_2), 24.7 (CH_3), 24.8 (CH_3), 27.9 (CH_2), 29.2 (CH_2), 29.4 (CH_2), 29.4 (CH_2), 30.4

(CH_2), 31.8 (CH_2), 54.1 (CHN), 55.7 (OCH_3), 58.1 (*C*HC=CH_2), 83.4 (OC), 113.8 (ArCH), 114.6 (ArCH), 126.0 (ArCH), 126.1 (ArCH), 126.9 (ArCH), 130.1 (ArCH), 132.4 (C=*C*H_2), 132.8 (ArC), 135.4 (ArC), 141.7 (ArC), 142.6 (Ar*C*OCH_3), (B*C*=CH_2 not observed); ^{11}B NMR (128 MHz, $CDCl_3$) δ ppm 10.2.

***N*-(Phenyl(1-(1-(4,4,5,5-tetramethyl-1,3,2-dioxaborolan-2-yl)vinyl)cyclohexyl)methyl)aniline (3v)**

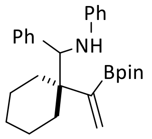

Prepared according to General Procedure 1, on a 0.258 mmol scale, preparative thin layer chromatography (CH_2Cl_2) afforded the title compound as a yellow gum (88.7 mg, 0.212 mmol, 82%).

Mp: 95-97 °C; MS (ES$^+$) *m/z*: 418 (M+H$^+$). HRMS calcd for $C_{27}H_{37}NBO_2$: 418.2917. Found: 418.2914; ν_{max} (thin film/cm^{-1}): 3412, 2929, 2855, 1600, 1499, 1451, 1371, 1299, 1213, 1141, 1119; ^{1}H NMR (400 MHz, $CDCl_3$) δ ppm 1.22 - 1.32 (m, 5 H, CH_2), 1.35 (s, 12 H, 4 x CH_3), 1.45 - 1.54 (m, 3 H, CH_2), 1.94 - 2.02 (m, 1 H, CH_2), 2.34 - 2.43 (m, 1 H, CH_2), 4.14 (s, 1 H, CHN), 5.29 (d, *J* = 2.0 Hz, 1 H, C=CH_2), 5.69 (br. s, 1 H, NH), 6.07 (d, *J* = 2.3 Hz, 1 H, C=CH_2), 6.40 (d, *J* = 7.6 Hz, 2 H, ArCH), 6.52 (t, *J* = 7.3 Hz, 1 H, ArCH), 6.97 - 7.04 (m, 2 H, ArCH), 7.16 - 7.27 (m, 5 H, ArCH); ^{13}C NMR (101 MHz, $CDCl_3$) δ ppm 21.8 (Cy), 22.6 (Cy), 24.7 (CH_3), 26.5 (Cy), 32.2 (Cy), 35.1 (Cy), 46.6 (*C*C=CH_2), 68.7 (CHN), 83.9 (OC), 112.4 (ArCH), 115.8 (ArCH), 126.6 (ArCH), 127.1 (ArCH), 128.8 (ArCH), 129.4 (ArCH), 132.9 (C=*C*H_2), 140.8 (ArC), 148.4 (ArC), (B*C*=CH_2 not observed); ^{11}B NMR (128 MHz, $CDCl_3$) δ ppm 11.0.

General Procedure 2 for the copper-catalysed borylation of allenes and trapping with an imine followed by an oxidative workup

***rac*-(3*S*,4*R*)-4-(Benzylamino)-3-cyclohexyl-4-(*o*-tolyl)butan-2-one (4a)**

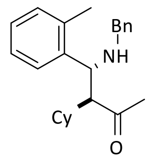

To a solution of IPrCuCl (6.3 mg, 0.013 mmol, 5 mol%) in THF (0.8 mL), was added *t*-BuOK (0.26 mL of a 1 M THF solution, 0.258 mmol, 1 equiv), and the reaction was stirred for 5 minutes at room temperature. B_2Pin_2 (72.1 mg, 0.284 mmol, 1.1 equiv) in THF (0.75 mL) was then added and the resulting mixture stirred for 30 min. A solution of (1,2-propadienyl)cyclohexane (47.3 mg, 0.387 mmol, 1.5 equiv) and (*E*)-*N*-benzyl-1-(*o*-tolyl)methanimine (54 mg, 0.258 mmol, 1 equiv) in THF (1 mL) were then added dropwise at -78 °C, and the reaction allowed to warm to room temperature

with stirring overnight. The reaction mixture was then filtered through a silica plug and concentrated *in vacuo.* To this crude mixture in THF (1.29 mL) at 0 °C was added H_2O_2 (0.14 mL of a 30% w:v aqueous solution, 1.29 mmol, 5 equiv) and NaOH (0.65 mL of a 2 M aqueous solution, 1.29 mmol, 5 equiv) and stirred for 20 min. The aqueous layer was then washed with Et_2O (3 x 2 mL) and dried over $MgSO_4$. Concentration *in vacuo* and purification by chromatography (3% EtOAc in hexanes) afforded the title compound as an oil (70 mg, 0.201 mmol, 78%).

MS (ES^+) *m/z*: 350 ($M+H^+$). HRMS calcd for $C_{24}H_{32}NO$: 350.2484. Found: 350.2471; ν_{max} (thin film/cm^{-1}): 3026, 2922, 2850, 1703, 1603, 1494, 1450, 1356, 1277, 1225, 1166, 1125, 1028; ^1H NMR (400 MHz, $CDCl_3$) δ ppm 0.78 - 0.99 (m, 2 H, CH_2), 1.04 - 1.32 (m, 3 H, CH_2), 1.50 - 1.71 (m, 4 H, CH_2), 1.75 (s, 3 H, CH_3), 1.79 - 1.93 (m, 1 H, CH_2), 2.11 (d, *J* = 14.5 Hz, 1 H, CH), 2.26 (s, 3 H, Ar-CH_3), 2.64 (dd, *J* = 7.9, 6.5 Hz, 1 H, CHC=O), 3.36 (d, *J* = 13.2 Hz, 1 H, Ph-CH_2), 3.71 (d, *J* = 13.2 Hz, 1 H, Ph-CH_2), 4.18 (d, *J* = 6.0 Hz, 1 H, CHN), 7.15 - 7.19 (m, 2 H, ArCH), 7.20 - 7.26 (m, 4 H, ArCH), 7.26 - 7.32 (m, 3 H, ArCH); ^{13}C NMR (101 MHz, $CDCl_3$) δ ppm 19.1 (Ar-*C*H_3), 26.2 (CH_2), 26.3 (CH_3), 26.4 (CH_2), 30.7 (CH_2), 31.3 (CH_2), 34.7 (CH_2), 37.5 (CH), 50.6 ($PhCH_2$), 55.2 (CHN), 62.7(*C*HC=O), 126.3 (ArCH), 126.7 (ArCH), 126.8 (ArCH), 128.1 (ArCH), 128.3 (ArCH), 130.8 (ArCH), 135.7 (ArC), 139.6 (ArC), 140.6 (ArC), 213.9 (C=O);

1-(1-((Benzylamino)(phenyl)methyl)cyclohexyl)ethan-1-one (4b)

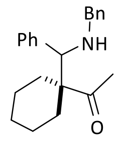

Prepared according to General Procedure 2, on a 0.258 mmol scale, column chromatography (3% EtOAc in Hexanes) afforded the title compound as a white solid (79.1 mg, 0.246 mmol, 95%).

Mp: 71-72 °C; MS (ES^+) *m/z*: 322 ($M+H^+$). HRMS calcd for $C_{22}H_{28}NO$: 322.2171. Found: 322.2179; ν_{max} (thin film/cm^{-1}): 3061, 3026, 2931, 2854, 1696, 1601, 1493, 1453, 1352, 1201, 1123, 1072, 1028, 1002; ^1H NMR (500 MHz, $CDCl_3$) δ ppm 0.87 - 0.98 (m, 1 H, CH_2), 0.98 - 1.08 (m, 1 H, CH_2), 1.09 - 1.18 (m, 2 H, CH_2), 1.22 - 1.42 (m, 2 H, CH_2), 1.47 - 1.59 (m, 3 H, CH_2), 2.03 (s, 3 H, CH_3), 2.11 (dd, *J* = 13.1, 2.7 Hz, 1 H, CH_2), 3.30 (d, *J* = 13.6 Hz, 1 H, Ph-CH_2), 3.61 - 3.68 (m, 2 H, Ph-CH_2 + CHN), 7.17 (d, *J* = 6.9 Hz, 2 H, ArCH), 7.24 (d, *J* = 6.9 Hz, 3 H, ArCH), 7.28 - 7.31 (m, 2 H, ArCH), 7.31 - 7.35 (m, 1 H, ArCH), 7.36 - 7.40 (m, 2 H, ArCH), (NH not observed); ^{13}C NMR (126 MHz, CD_3OH) δ ppm 22.8 (Cy), 23.6 (Cy), 25.8 (Cy), 26.3 (CH_3), 27.5 (Cy), 32.8 (Cy), 51.2 ($PhCH_2$), 56.8 (*C*C=O), 67.8 (CHN), 126.9 (ArCH), 127.4 (ArCH), 127.9 (ArCH), 128.2 (ArCH), 128.4 (ArCH), 129.3 (ArCH), 138.8 (ArC), 140.1 (ArC), 212.8 (C=O).

rac-(3_R_,4_S_)-3-Cyclohexyl-4-((4-methoxyphenyl)amino)-4-(_o_-tolyl)butan-2-one (4c)

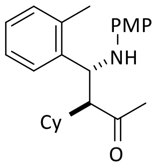

Prepared according to General Procedure 2, on a 0.070 mmol scale, column chromatography (3% EtOAc in Hexanes) afforded the title compound as a yellow gum (25.6 mg, 0.070 mmol, 99%).

MS (ES$^+$) *m/z*: 366 (M+H$^+$). HRMS calcd for $C_{24}H_{32}NO_2$: 366.2433. Found: 366.2415; ν_{max} (thin film/cm^{-1}): 3407, 2924, 2851, 1702, 1511, 1484, 1359, 1241, 1225, 1168, 1110, 1039; ^{1}H NMR (400 MHz, $CDCl_3$) δ ppm 0.79 - 1.42 (m, 6 H, CH_2), 1.54 (s, 3 H, CH_3), 1.57 - 1.80 (m, 3 H, CH_2), 1.93 - 2.13 (m, 2 H, CH_2 + CH), 2.50 (s, 3 H, Ar-CH_3), 2.78 (dd, *J* = 10.3, 3.3 Hz, 1 H, CHC=O), 3.68 (s, 3 H, OCH_3), 4.86 (d, *J* = 3.5 Hz, 1 H, CHN), 5.25 (br. s., 1 H, NH), 6.36 (d, *J* = 8.8 Hz, 2 H, ArCH), 6.62 - 6.71 (m, 2 H, ArCH), 7.02 - 7.15 (m, 3 H, ArCH), 7.16 - 7.21 (m, 1 H, ArCH); ^{13}C NMR (101 MHz, $CDCl_3$) δ ppm 18.9 (Ar-CH_3), 26.0 (2 x Cy), 26.3 (CH_2), 29.8 (CH_2), 32.2 (*C*H_3C=O), 35.5 (CH_2), 37.6 (CH_2), 51.8 (CHN), 55.7 (OCH_3), 60.6 (*C*HC=O), 113.3 (ArCH), 114.8 (ArCH), 125.9 (ArCH), 126.5 (ArCH), 126.9 (ArCH), 130.8 (ArCH), 134.1 (ArC), 139.3 (ArC), 141.0 (ArC), 151.2 (ArC), 215.4 (C=O).

rac-_N_-((1_R_,2_R_)-2-Cyclohexyl-3-(4,4,5,5-tetramethyl-1,3,2-dioxaborolan-2-yl)-1-(_o_-tolyl)but-3-en-1-yl)-4-methoxybenzenaminium trifluoromethanesulfonate (5a)

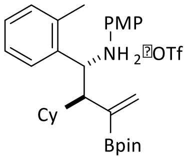

To a solution of **3m** (50 mg, 0.105 mmol, 1 equiv) in CH_2Cl_2 (0.62 mL) at -30 °C, was added triflic acid (15.8 mg, 0.105 mmol, 1 equiv) in CH_2Cl_2 (0.093 mL), dropwise. The reaction mixture was allowed to warm to room temperature over 2 h before being concentrated *in vacuo* and recrystallized from pentane to give the title compound as brown crystals (58.5 mg, 0.094 mmol, 89%).

Mp: 123-125 °C (Pentane); MS (ES$^+$) *m/z*: 476 (M – OTf); ν_{max} (thin film/cm^{-1}): 2976, 2927, 2853, 1606, 1513, 1449, 1424, 1392, 1373, 1362, 1247, 1167, 1138, 1029; ^{1}H NMR (400 MHz, $CDCl_3$) δ ppm 0.76 - 1.19 (m, 7 H, CH_2), 1.22 – 1.37 (m, 1 H, CH_2), 1.43 (s, 6 H, CH_3), 1.44 (s, 6 H, CH_3), 1.49 - 1.74 (m, 3 H, CH_2 + CH), 1.88 (s, 3 H, Ar-CH_3), 3.14 (d, *J* = 11.9 Hz, 1 H, C*H*C=CH_2), 3.74 (s, 3 H, OCH_3), 5.33 (t, *J* = 8.8 Hz, 1 H, C*H*N), 6.24 (br. s., 1 H, C=CH_2), 6.31 (s, 1 H, C=CH_2), 6.71 (d, *J* = 9.1 Hz, 2 H, ArCH), 6.99 (d, *J* = 7.6 Hz, 1 H, ArCH), 7.04 (d, *J* = 8.8 Hz, 2 H, ArCH), 7.23 (s, 1 H, ArCH), 7.41 (s, 1 H, ArCH), 7.87 (d, *J* = 7.6 Hz, 1 H, ArCH), 10.33 (br. s, 1 H, NH); ^{13}C NMR (126 MHz, $CDCl_3$)

δ ppm 19.1 (Ar-CH_3), 24.4 (CH_3), 25.4 (CH_3), 25.9 (CH_2), 26.0 (CH_2), 26.2 (CH_2), 29.0 (CH_2), 31.8 (CH_2), 39.0 (CH), 55.5 (OCH_3), 57.6 (*C*HC=CH_2), 65.6 (CHN), 84.7 (OC), 114.5 (ArCH), 124.7 (ArCH), 125.9 (ArC), 127.2 (ArCH), 127.9 (ArCH), 129.5 (ArCH), 130.6 (ArCH), 131.1 (ArC), 137.8 (ArC), 140.1 (C=*C*H_2), 160.1 (Ar*C*OCH_3), (B*C*=CH_2 not observed); ^{11}B NMR (128 MHz, $CDCl_3$) δ 4.8.

N-(2,2-Dimethyl-1-phenyl-3-(4,4,5,5-tetramethyl-1,3,2-dioxaborolan-2-yl)but-3-en-1-yl)-4-methylbenzenesulfonamide (3w)

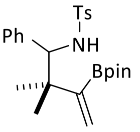

To a solution of IPrCuCl (50 mg, 0.103 mmol, 1 equiv) in THF (1.5 mL), was added KO*t*-Bu (0.11 mL of a 1 M solution in THF, 0.108 mmol, 1 equiv) and stirred for 5 minutes. B_2pin_2 (28.6 mg, 0.128 mmol, 1.1 equiv) in THF (0.5 mL) was added and stirred for 10 minutes. 3-Methyl-1,2-butadiene (8.4 mg, 0.012 mL, 0.123 mmol, 1.2 equiv) was added neat and stirred for 5 minutes. (*E*)-*N*-benzylidene-4-methylbenzenesulfonamide (40 mg, 0.154 mmol, 1.5 equiv) in THF (0.5 mL) was then added and the reaction stirred at room temperature for 18 hours. The reaction mixture was then filtered through a silica plug, concentrated *in vacuo* and purified by chromatography (10% EtOAc in hexanes) to afford the title compound (28 mg, 61.92 µmol, 56%).

Mp: 128-130 °C (Pentane); MS (ES$^+$) *m/z*: 456 (M+H$^+$). HRMS calcd for $C_{25}H_{35}NO_4BS$: 456.2385. Found: 456.2365; ν_{max} (thin film/cm^{-1}): 3288, 2976, 2927, 1600, 1495, 1457, 1412, 1355, 1325, 1302, 1214, 1160, 1144, 1114, 1095, 1056, 1030; ^{1}H NMR (400 MHz, $CDCl_3$) δ ppm 0.98 (s, 3 H, CH_3), 1.10 (s, 3 H, CH_3), 1.36 (s, 12 H, CH_3), 2.30 (s, 3 H, Ar-CH_3), 4.40 (d, *J* = 6.7 Hz, 1 H, CHN), 5.31 (s, 1 H, C=CH_2), 5.82 (d, *J* = 1.8 Hz, 1 H, C=CH_2), 6.33 (d, *J* = 6.2 Hz, 1 H, NH), 6.90 (d, *J* = 7.0 Hz, 2 H, ArH), 6.96 - 7.10 (m, 5 H, ArH), 7.37 (d, *J* = 8.1 Hz, 2 H, ArH); ^{13}C NMR (101 MHz, $CDCl_3$) δ 21.4 (Ar-CH_3), 24.3 (CH_3), 24.6 (CH_3), 24.8 (CH_3), 26.2 (CH_3), 42.8 (*C*C=CH_2), 65.7 (CHN), 84.2 (OC), 126.6 (ArCH), 126.90 (ArCH), 126.92 (ArCH), 128.8 (ArCH), 130.7 (C=*C*H_2), 137.8 (ArC), 138.3 (ArC), 142.2 (ArC), (B*C*=CH_2 not observed).

***rac*-N-((1*R*,2*S*)-3-(Dimethyl(phenyl)silyl)-1,2-diphenylbut-3-en-1-yl)aniline (3x)**

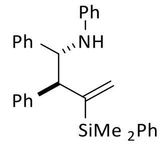

To a solution of IMes•BF_4 (10.9 mg, 0.026 mmol, 10 mol%) and CuI (2.5 mg, 0.013 mmol, 5 mol%) in THF (0.8 mL), was added KO*t*-Bu (0.043 mL of a 1 M THF solution, 0.043 mmol, 16.5 mol%), and

the reaction was stirred for 1 hour at room temperature. The resulting solution was transferred to a vessel containing anhydrous Cs_2CO_3 (84.1 mg, 0.263 mmol, 1 equiv) in THF (1 ml). $PhMe_2SiBpin$ (0.079 mL, 0.284 mmol, 1.1 equiv) was then added and the resulting mixture stirred for 15 min. A solution of (propa-1,2-dien-1-yl)benzene (45 mg, 0.387 mmol, 1.5 equiv) and (*E*)-*N*,1-diphenylmethanimine (46.8 mg, 0.263 mmol, 1 equiv) in THF (0.5 mL) was then added dropwise, and the reaction stirred at room temperature for 18 hours. The reaction mixture was filtered through a silica plug, concentrated *in vacuo* and purified by chromatography (2% EtOAc in hexanes) to afford the title compound (77mg, 0.178 mmol, 69%).

MS (ES^+) *m/z*: 434 ($M+H^+$). HRMS calcd for $C_{30}H_{31}NSiNa$: 456.2123. Found: 456.2111; ν_{max} (thin film/cm^{-1}): 3409, 3026, 2956, 1600, 1502, 1453, 1427, 1316, 1249, 1179, 1155, 1110, 1076, 1028; ^1H NMR (400 MHz, $CDCl_3$) δ ppm -0.02 (s, 3 H, $SiCH_3$), 0.13 (s, 3 H, $SiCH_3$), 3.46 (d, *J* = 10.3 Hz, 1 H, C*H*C=CH_2), 3.90 (br. s, 1 H, NH), 4.39 (d, *J* = 10.3 Hz, 1 H, CHN), 5.71 (d, *J* = 1.5 Hz, 1 H, C=CH_2), 5.97 (s, 1 H, C=CH_2), 6.23 (dd, *J* = 8.6, 1.0 Hz, 2 H, ArCH), 6.47 (t, *J* = 7.3 Hz, 1 H, ArCH), 6.63 (dd, *J* = 7.7, 1.6 Hz, 2 H, ArCH), 6.74 - 6.80 (m, 2 H, ArCH), 6.83 - 6.98 (m, 8 H, ArCH), 7.22 - 7.30 (m, 3 H, ArCH), 7.32 - 7.38 (m, 2 H, ArCH); ^{13}C NMR (101 MHz, $CDCl_3$) δ ppm -3.5 ($SiCH_3$), -3.0 ($SiCH_3$), 58.1 (CHN), 62.0 (*C*HC=CH_2), 113.4 (ArCH), 117.3 (ArCH), 126.4 (ArCH), 126.5 (ArCH), 126.9 (C=*C*H_2), 127.3 (ArCH), 127.6 (ArCH), 127.7 (ArCH), 128.0 (ArCH), 128.9 (ArCH), 129.3 (ArCH), 129.5 (ArCH), 133.9 (ArCH), 137.5 (ArC), 139.4 (ArC), 142.4 (ArC), 147.6 (ArC), 149.9 (*C*=CH_2).

NMR spectra

rac-N-((1*R*,2*S*)-1,2-Diphenyl-3-(4,4,5,5-tetramethyl-1,3,2-dioxaborolan-2-yl)but-3-en-1-yl)aniline (3a)

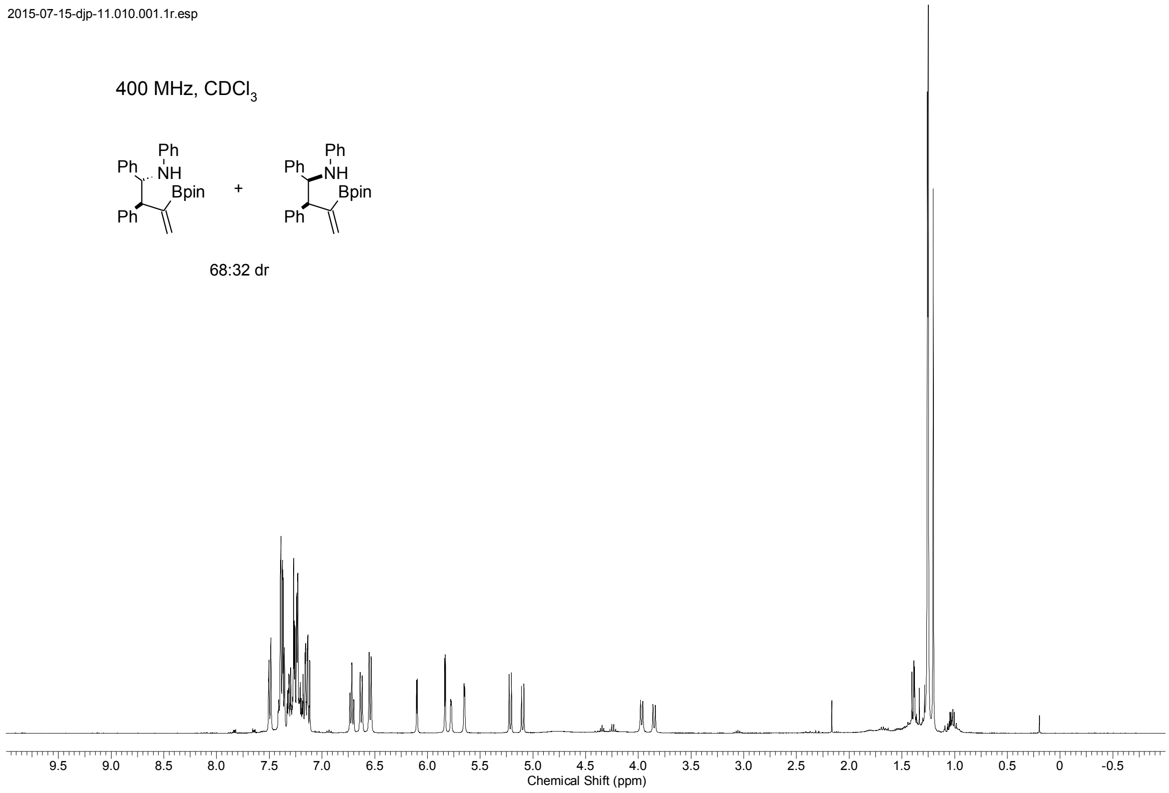

rac-N-((1*R*,2*S*)-1,2-Diphenyl-3-(4,4,5,5-tetramethyl-1,3,2-dioxaborolan-2-yl)but-3-en-1-yl)aniline (3a)

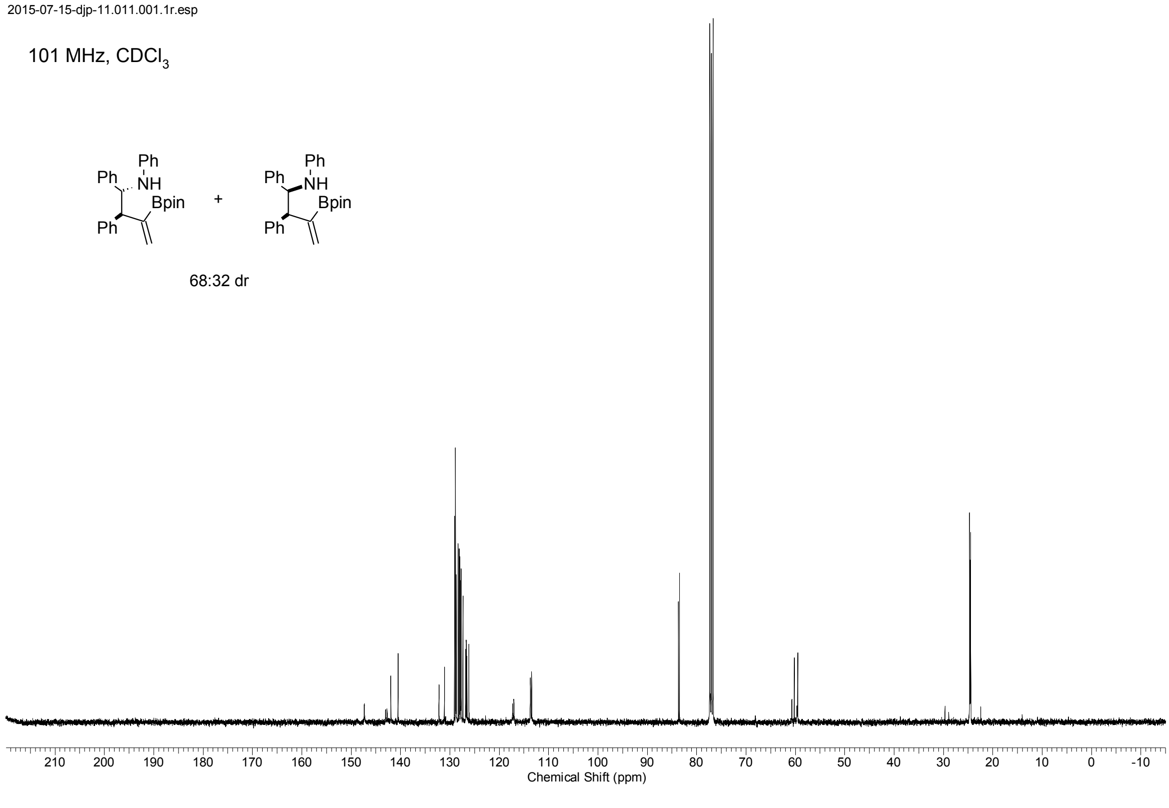

***rac-N*-((1*R*,2*R*)-2-Cyclohexyl-1-phenyl-3-(4,4,5,5-tetramethyl-1,3,2-dioxaborolan-2-yl)but-3-en-1-yl)aniline (3b)**

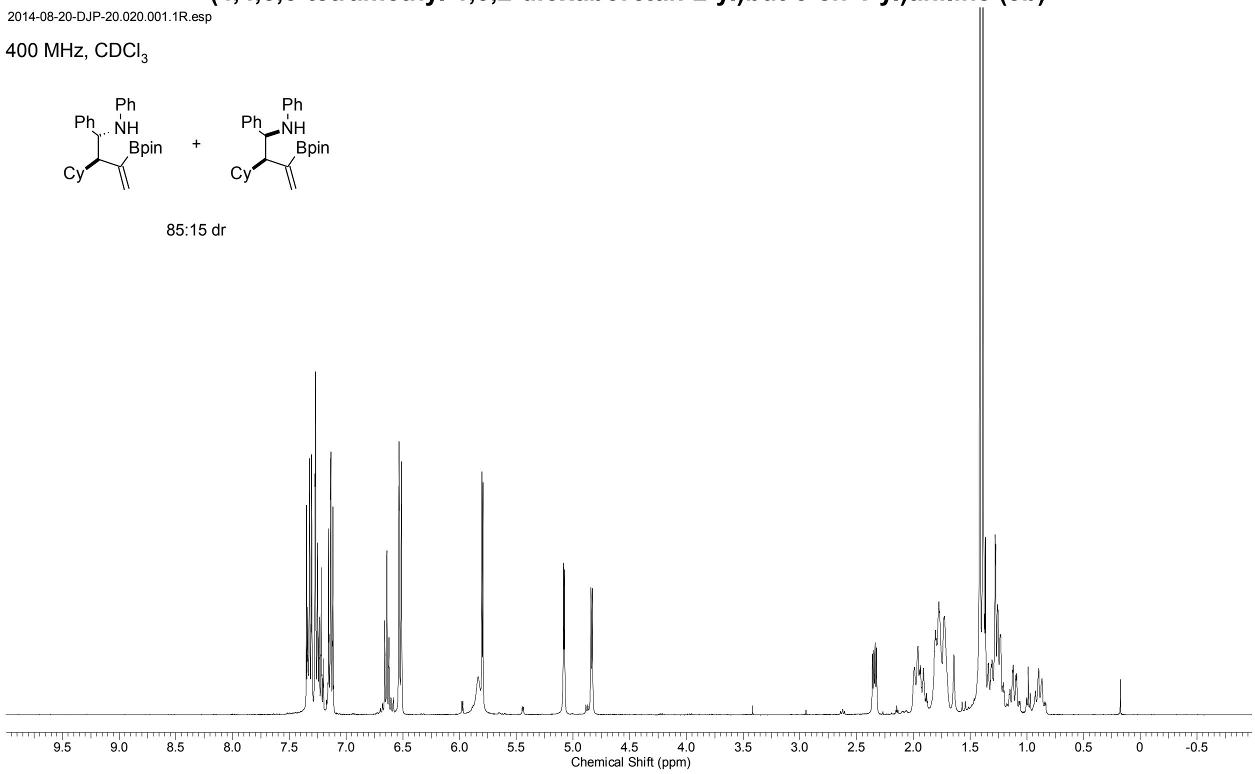

***rac-N*-((1*R*,2*R*)-2-Cyclohexyl-1-phenyl-3-(4,4,5,5-tetramethyl-1,3,2-dioxaborolan-2-yl)but-3-en-1-yl)aniline (3b)**

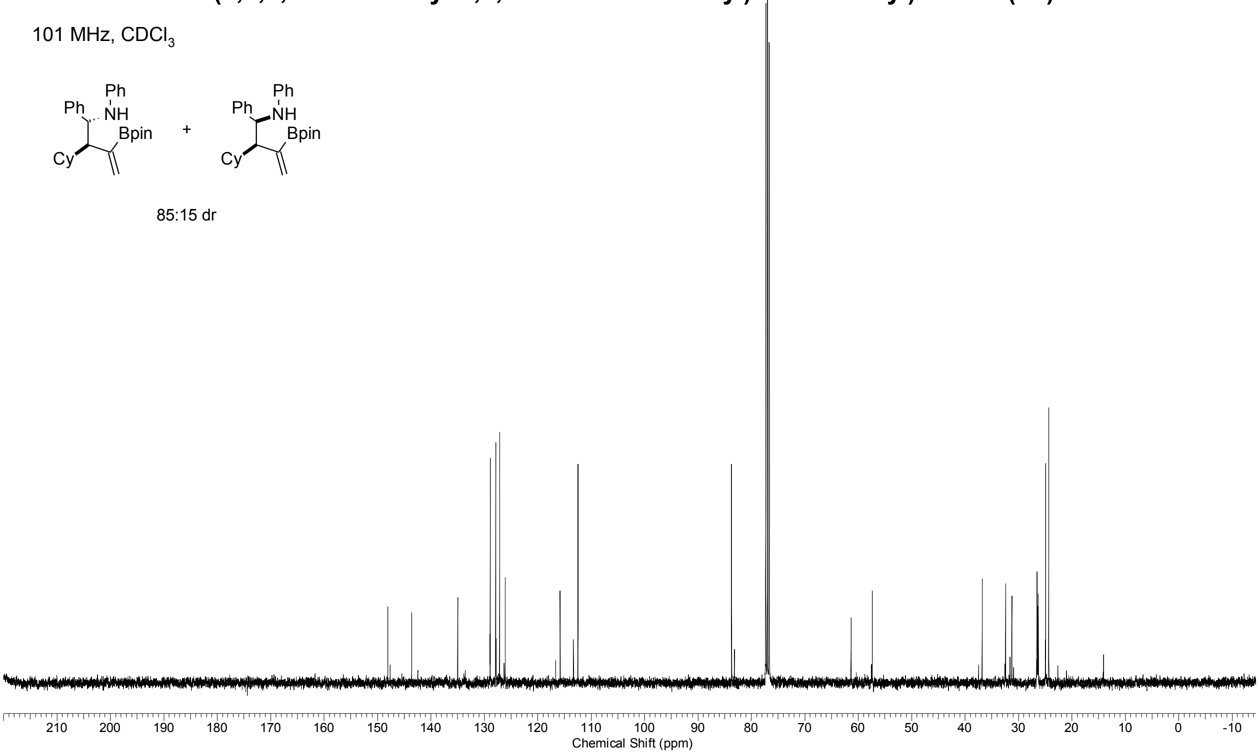

2015-01-07-DJP-8.010.001.1R.esp

rac-***N***-((1*R*,2*R*)-2-(Cyclohexylmethyl)-1-phenyl-3-(4,4,5,5-tetramethyl-1,3,2-dioxaborolan-2-yl)but-3-en-1-yl)aniline (3c)

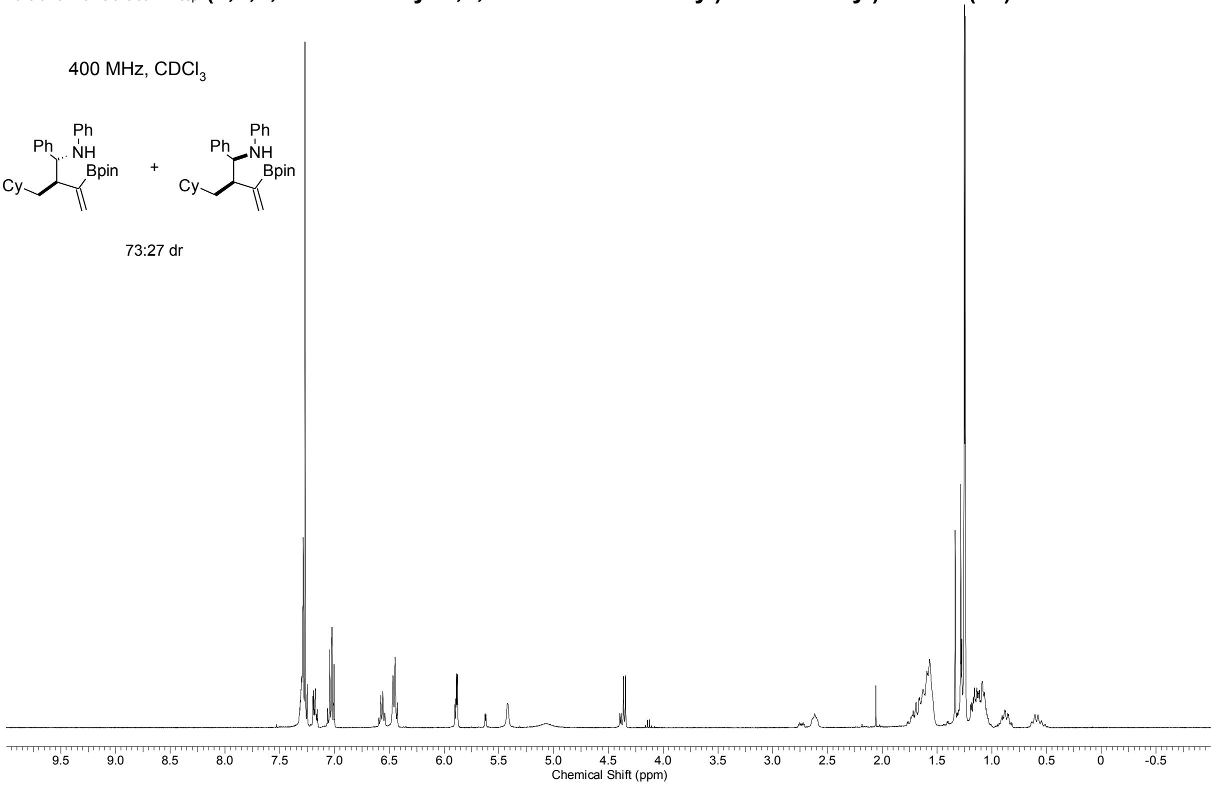

2015-03-05-djp-15.010.001.1r.esp

rac-***N***-((1*R*,2*R*)-2-(Cyclohexylmethyl)-1-phenyl-3-(4,4,5,5-tetramethyl-1,3,2-dioxaborolan-2-yl)but-3-en-1-yl)aniline (3c)

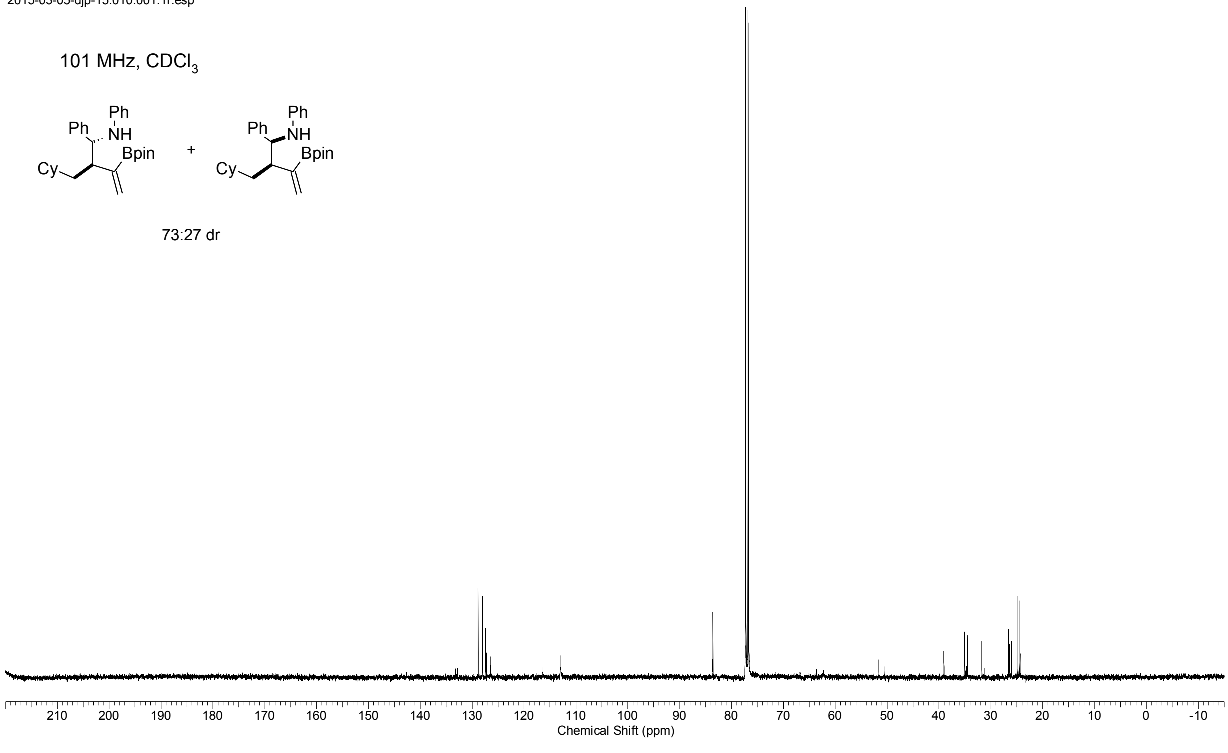

2014-11-28-DJP-28.010.001.1R.esp

rac-N-((1*R*,2*R*)-1-Phenyl-2-(1-(4,4,5,5-tetramethyl-1,3,2-dioxaborolan-2-yl)vinyl)decyl)aniline (3d)

500 MHz, $CDCl_3$

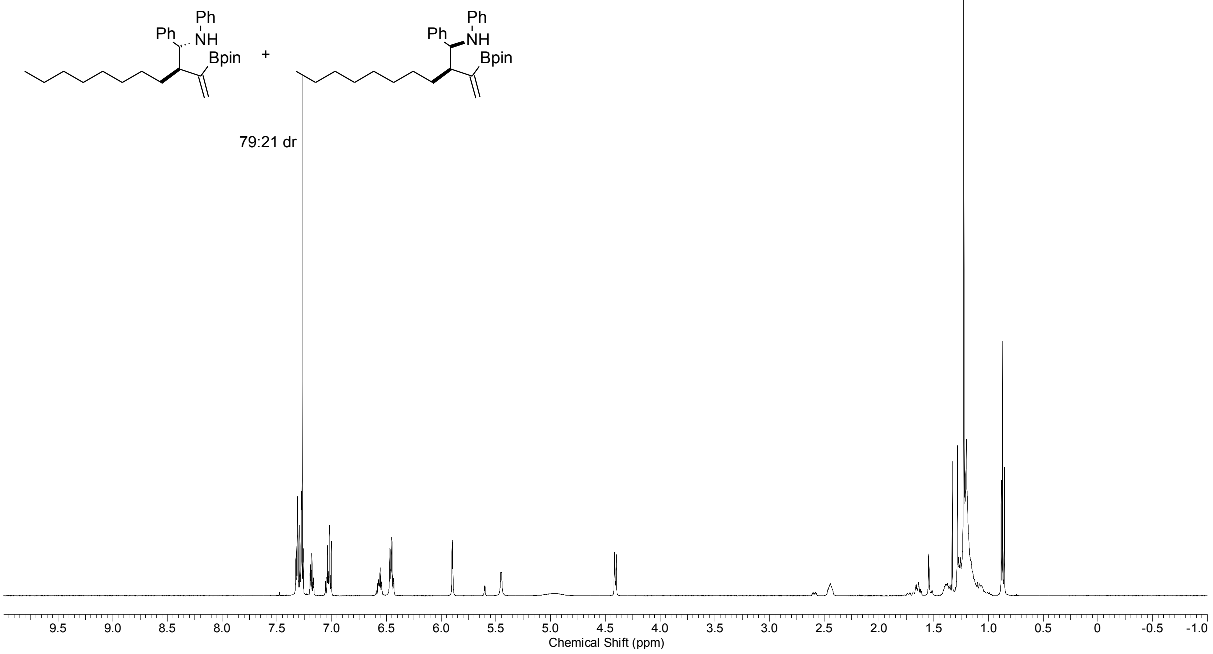

2014-10-16-DJP-4.011.001.1R.esp

rac-N-((1*R*,2*R*)-1-Phenyl-2-(1-(4,4,5,5-tetramethyl-1,3,2-dioxaborolan-2-yl)vinyl)decyl)aniline (3d)

126 MHz, $CDCl_3$

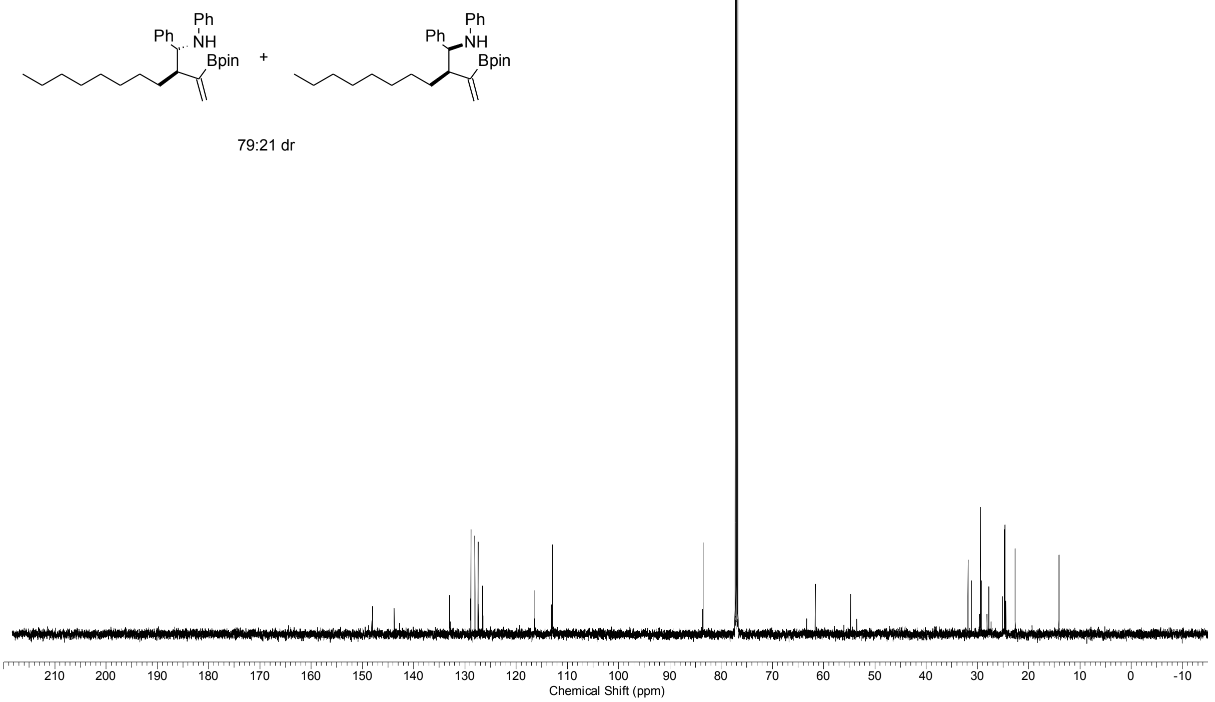

rac-N-((1*R*,2*R*)-2-Cyclohexyl-1-phenyl-3-(4,4,5,5-tetramethyl-1,3,2-dioxaborolan-2-yl)but-3-en-1-yl)-4-methoxyaniline (3e)

2014-10-14-DJP-1.030.001.1R.esp

500 MHz, $CDCl_3$

84:16 dr

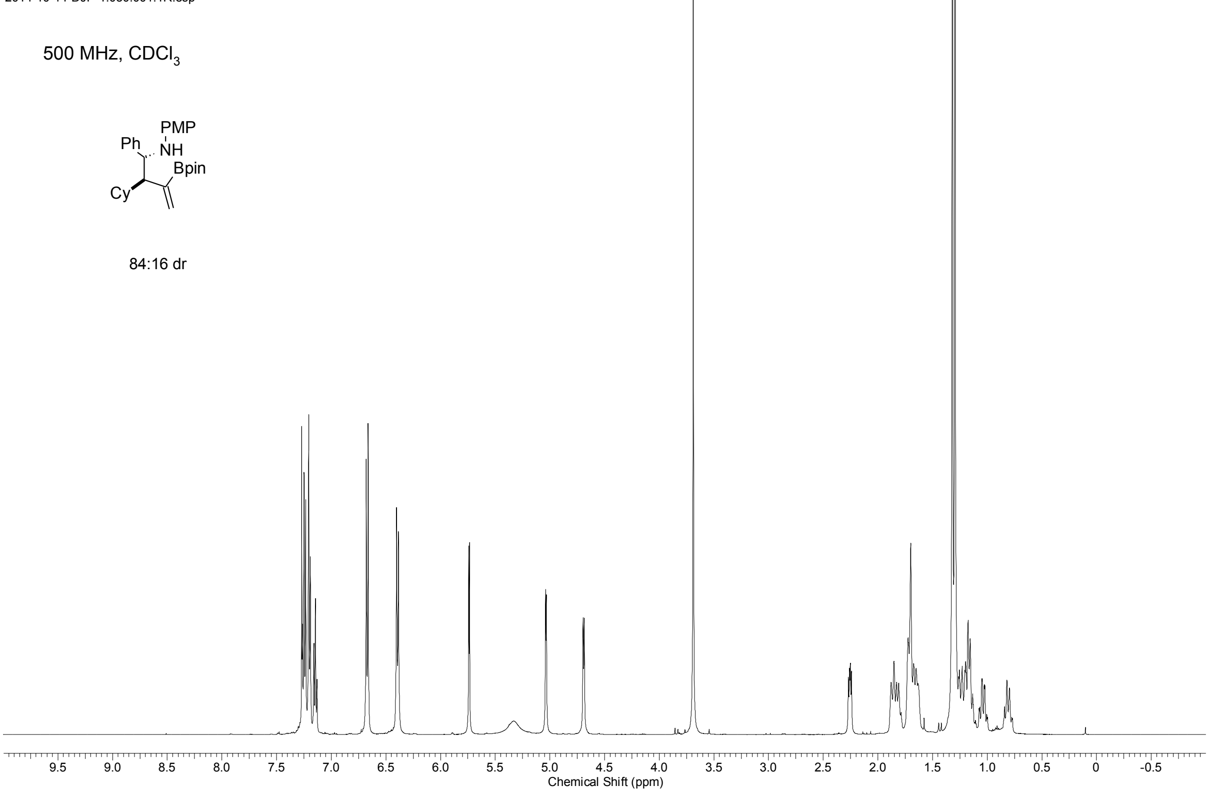

rac-N-((1*R*,2*R*)-2-Cyclohexyl-1-phenyl-3-(4,4,5,5-tetramethyl-1,3,2-dioxaborolan-2-yl)but-3-en-1-yl)-4-methoxyaniline (3e)

2014-10-14-DJP-1.032.001.1R.esp

126 MHz, $CDCl_3$

84:16 dr

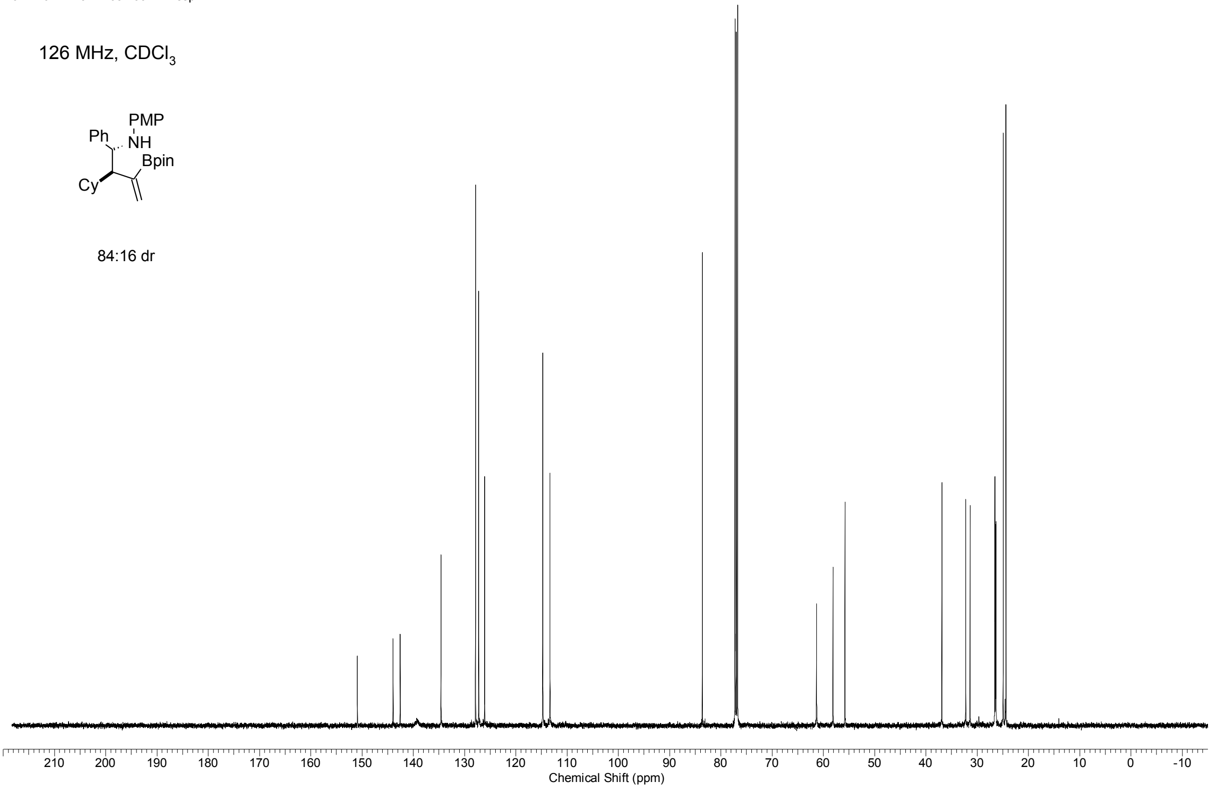

rac-_N_-((1_R_,2_R_)-2-Cyclohexyl-1-(4-methoxyphenyl)-3-(4,4,5,5-tetramethyl-1,3,2-dioxaborolan-2-yl)but-3-en-1-yl)-4-methoxyaniline (3f)

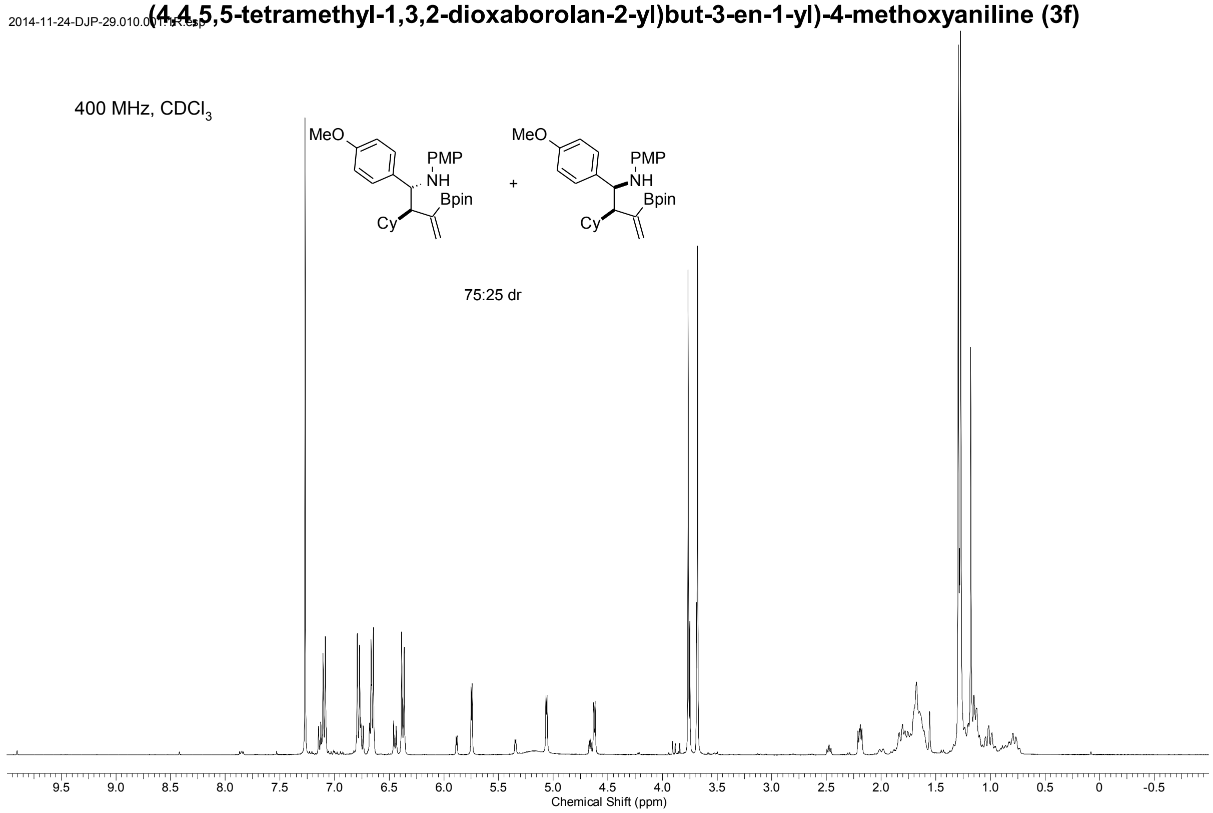

rac-_N_-((1_R_,2_R_)-2-Cyclohexyl-1-(4-methoxyphenyl)-3-(4,4,5,5-tetramethyl-1,3,2-dioxaborolan-2-yl)but-3-en-1-yl)-4-methoxyaniline (3f)

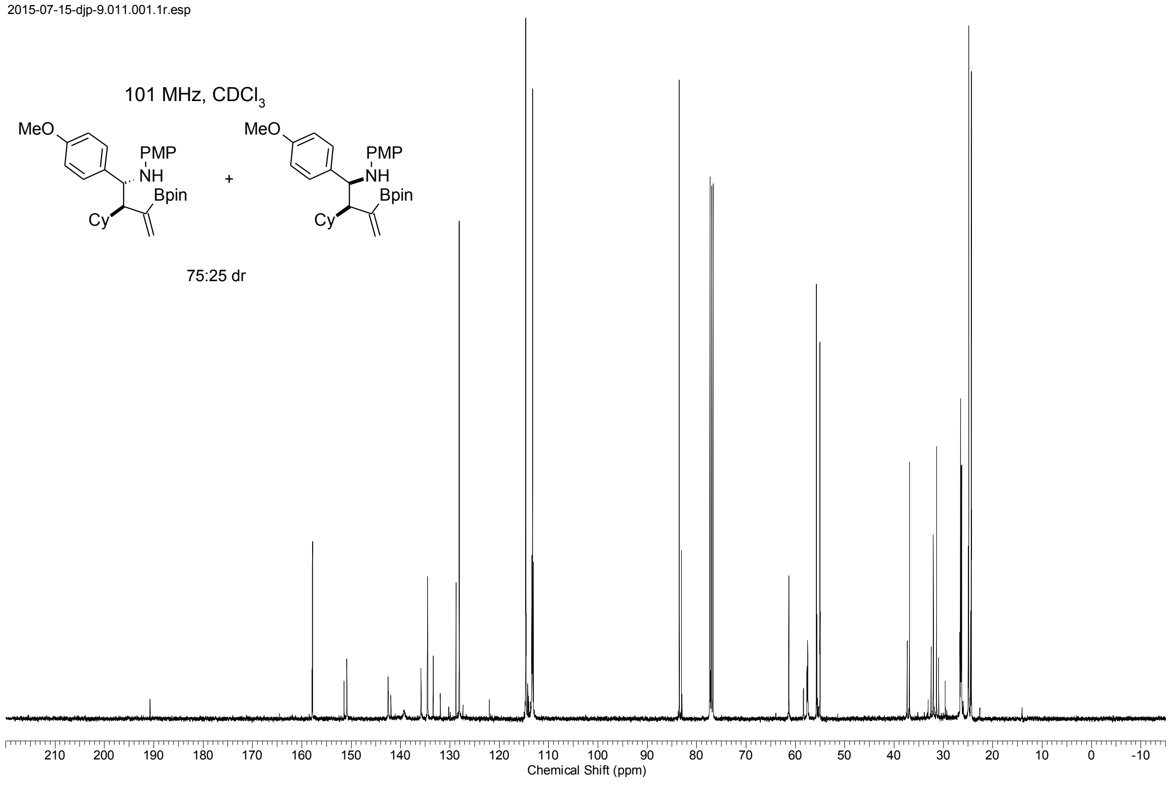

rac-*N*-((1*R*,2*R*)-2-Cyclohexyl-3-(4,4,5,5-tetramethyl-1,3,2-dioxaborolan-2-yl)-1-(4-(trifluoromethyl)phenyl)but-3-en-1-yl)-4-methoxyaniline (3g)

2014-11-19-DJP-59.010.001.1R.esp

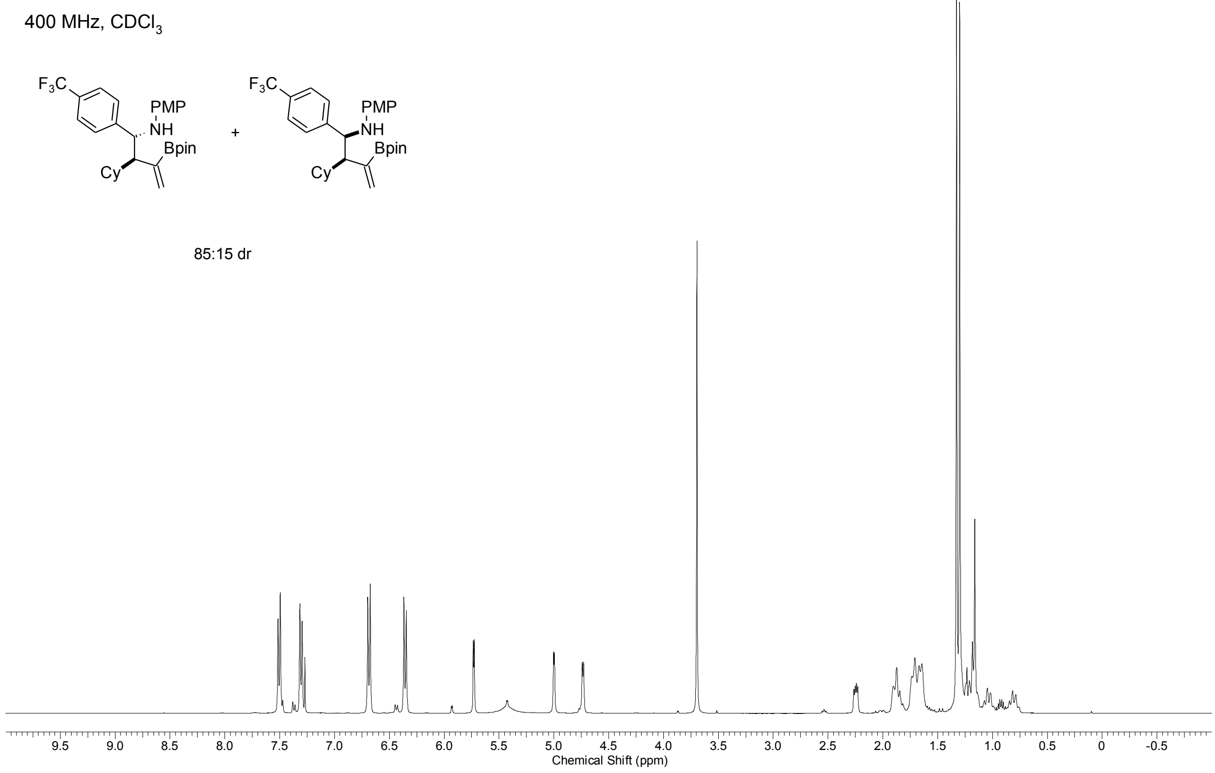

rac-*N*-((1*R*,2*R*)-2-Cyclohexyl-3-(4,4,5,5-tetramethyl-1,3,2-dioxaborolan-2-yl)-1-(4-(trifluoromethyl)phenyl)but-3-en-1-yl)-4-methoxyaniline (3g)

2015-01-08-djp-16.011.001.1r.esp

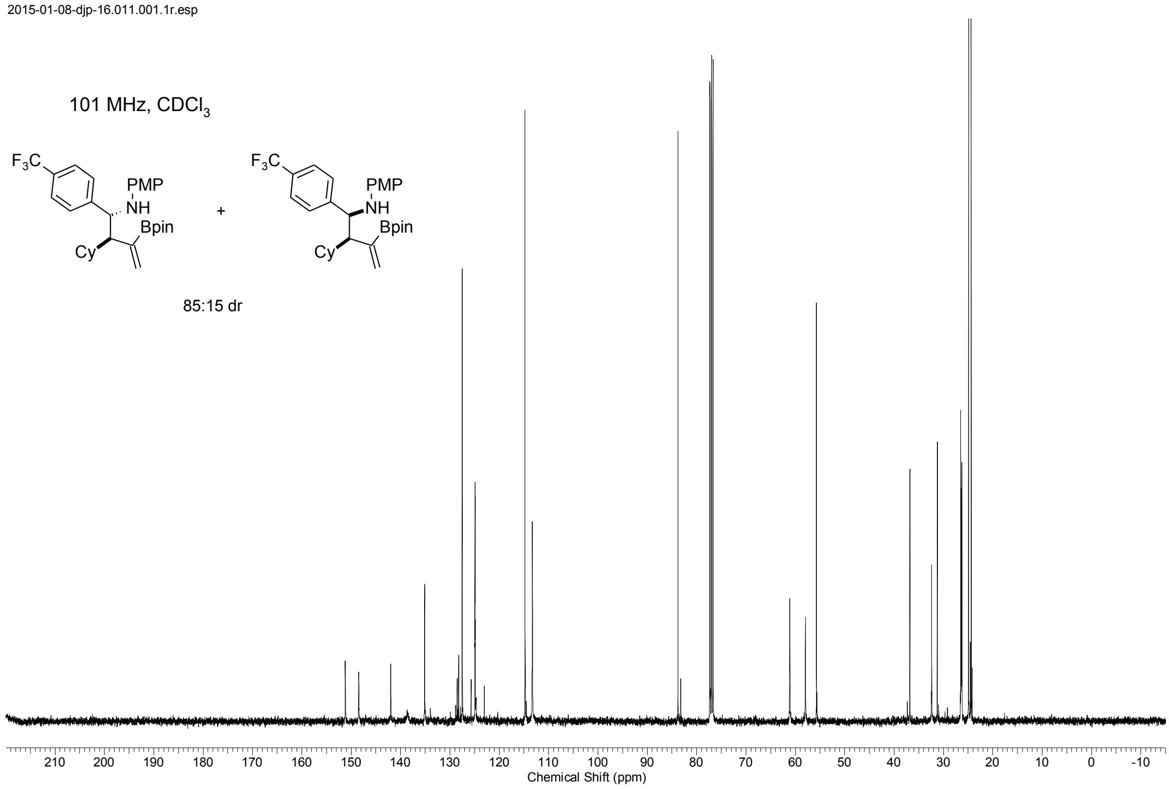

rac-N-((1_R_,2_R_)-1-(4-Bromophenyl)-2-cyclohexyl-3-(4,4,5,5-tetramethyl-1,3,2-dioxaborolan-2-yl)but-3-en-1-yl)-4-methoxyaniline (3h)

2015-02-27-djp-56.010.001.1r.esp

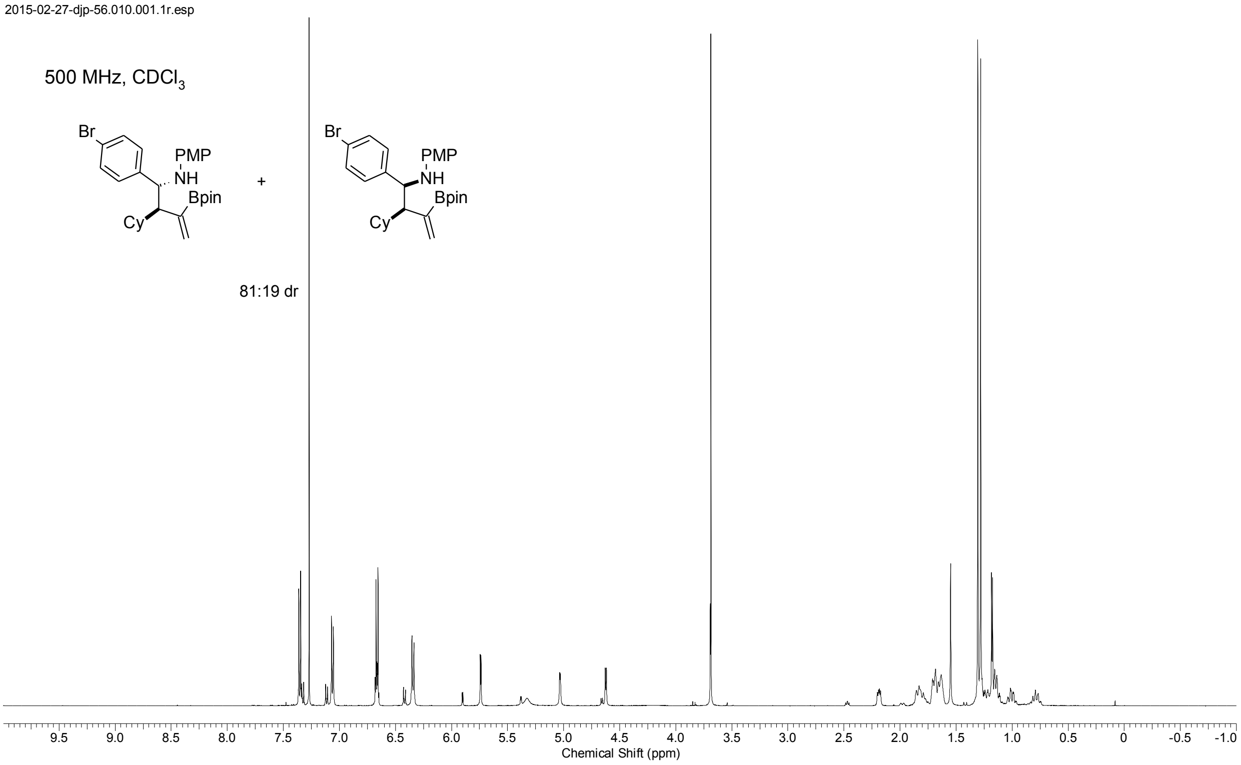

rac-N-((1_R_,2_R_)-1-(4-Bromophenyl)-2-cyclohexyl-3-(4,4,5,5-tetramethyl-1,3,2-dioxaborolan-2-yl)but-3-en-1-yl)-4-methoxyaniline (3h)

2015-02-27-djp-56.012.001.1r.esp

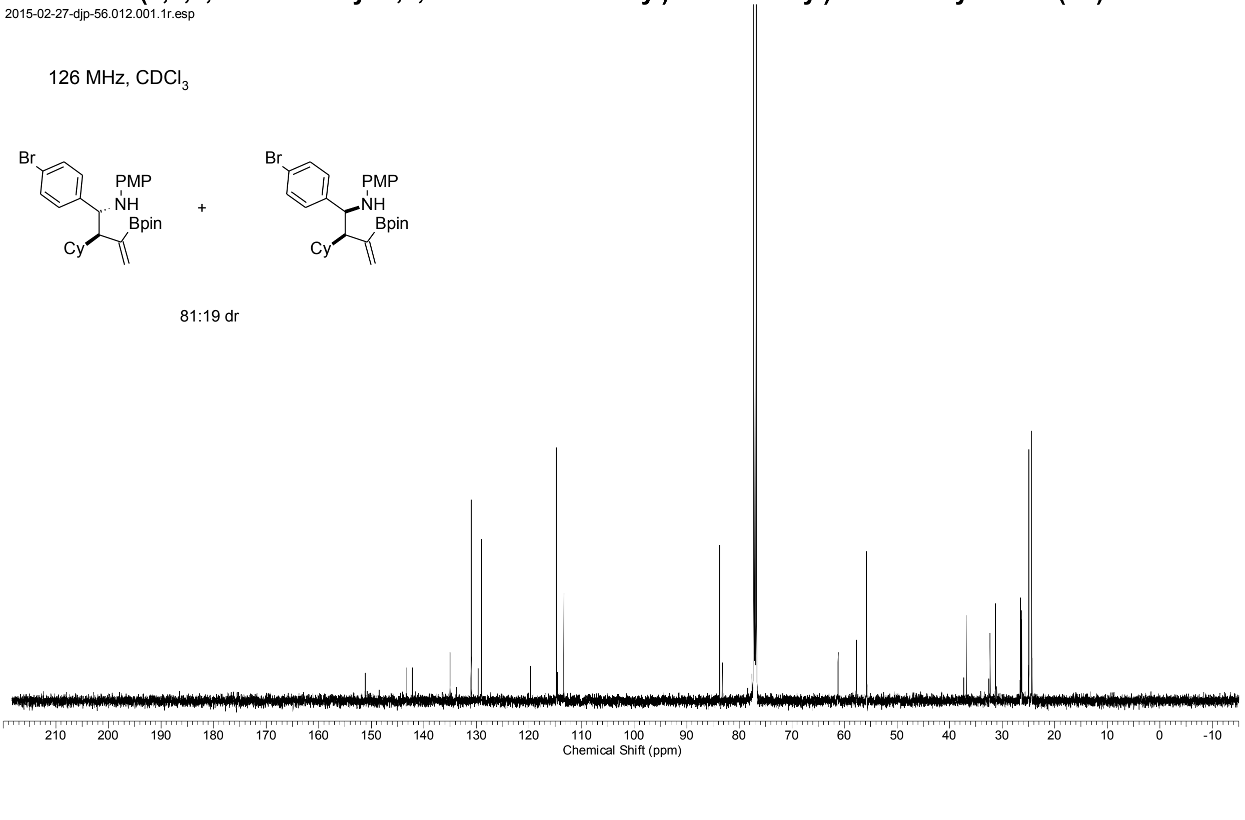

***rac-N*-((1*R*,2*R*)-2-Cyclohexyl-1-(furan-2-yl)-3-(4,4,5,5-tetramethyl-1,3,2-dioxaborolan-2-yl)but-3-en-1-yl)-4-methoxyaniline (3i)**

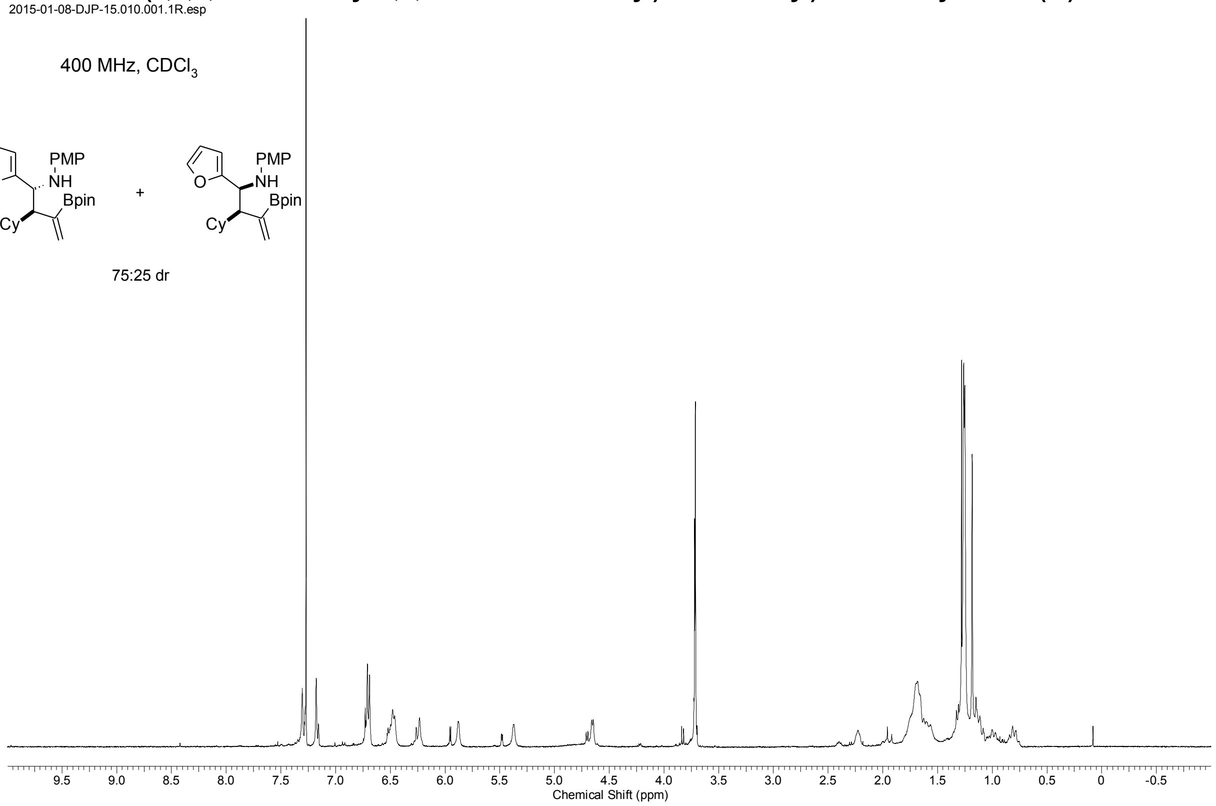

***rac-N*-((1*R*,2*R*)-2-Cyclohexyl-1-(furan-2-yl)-3-(4,4,5,5-tetramethyl-1,3,2-dioxaborolan-2-yl)but-3-en-1-yl)-4-methoxyaniline (3i)**

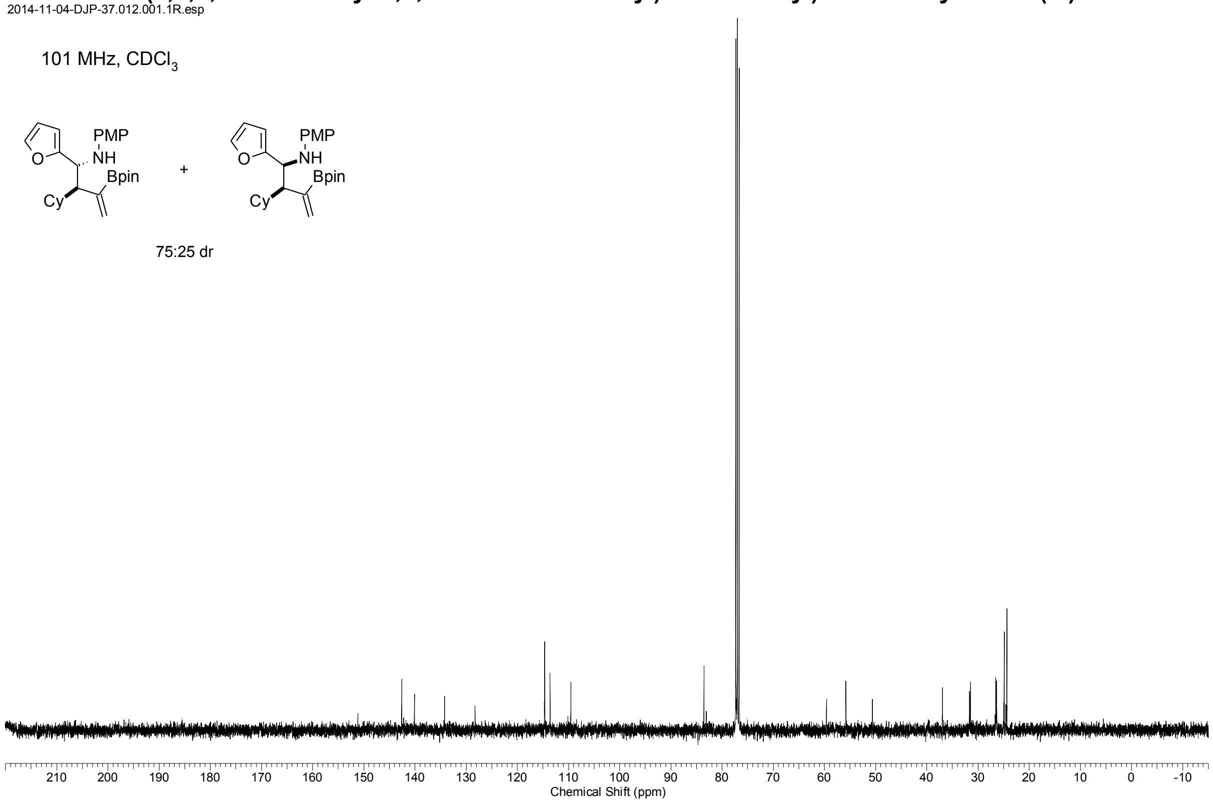

rac-N-((1*R*,2*R*)-2-Cyclohexyl-3-(4,4,5,5-tetramethyl-1,3,2-dioxaborolan-2-yl)-1-(thiophen-2-yl)but-3-en-1-yl)-4-methoxyaniline (3j)

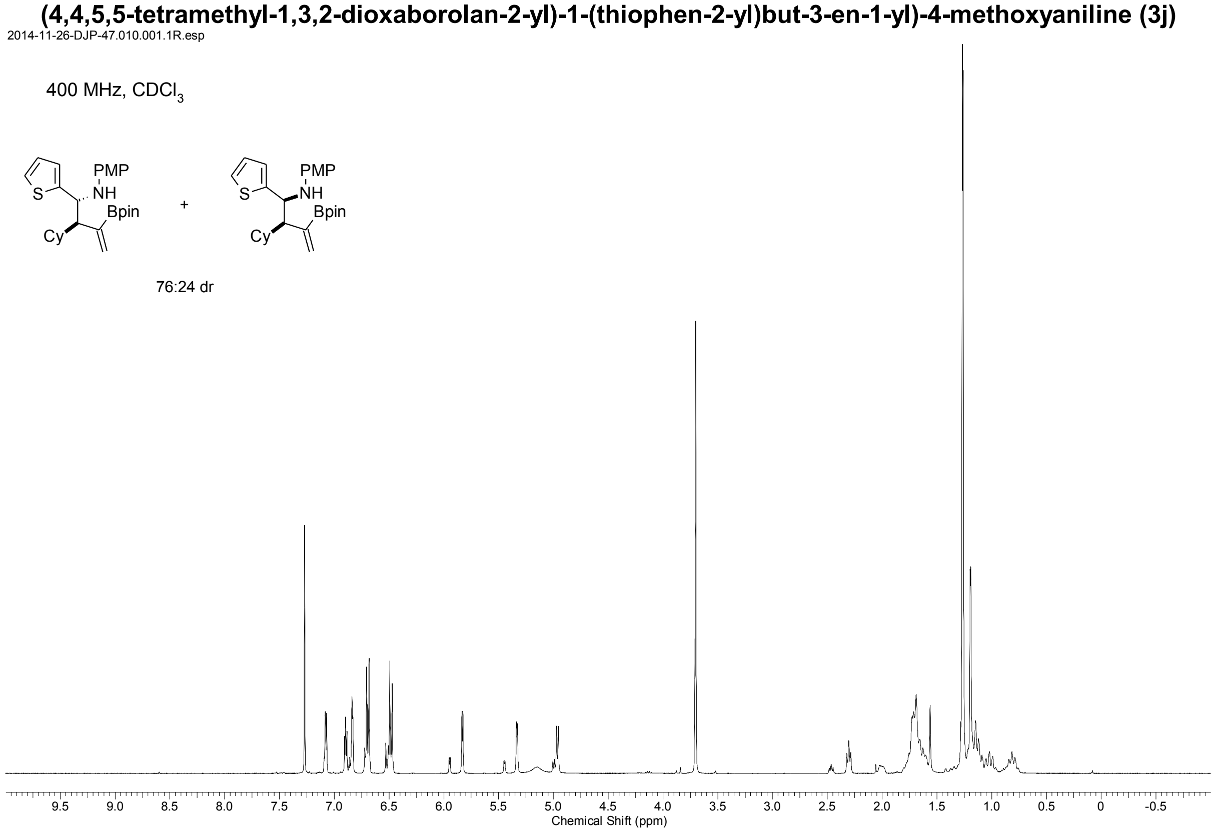

rac-N-((1*R*,2*R*)-2-Cyclohexyl-3-(4,4,5,5-tetramethyl-1,3,2-dioxaborolan-2-yl)-1-(thiophen-2-yl)but-3-en-1-yl)-4-methoxyaniline (3j)

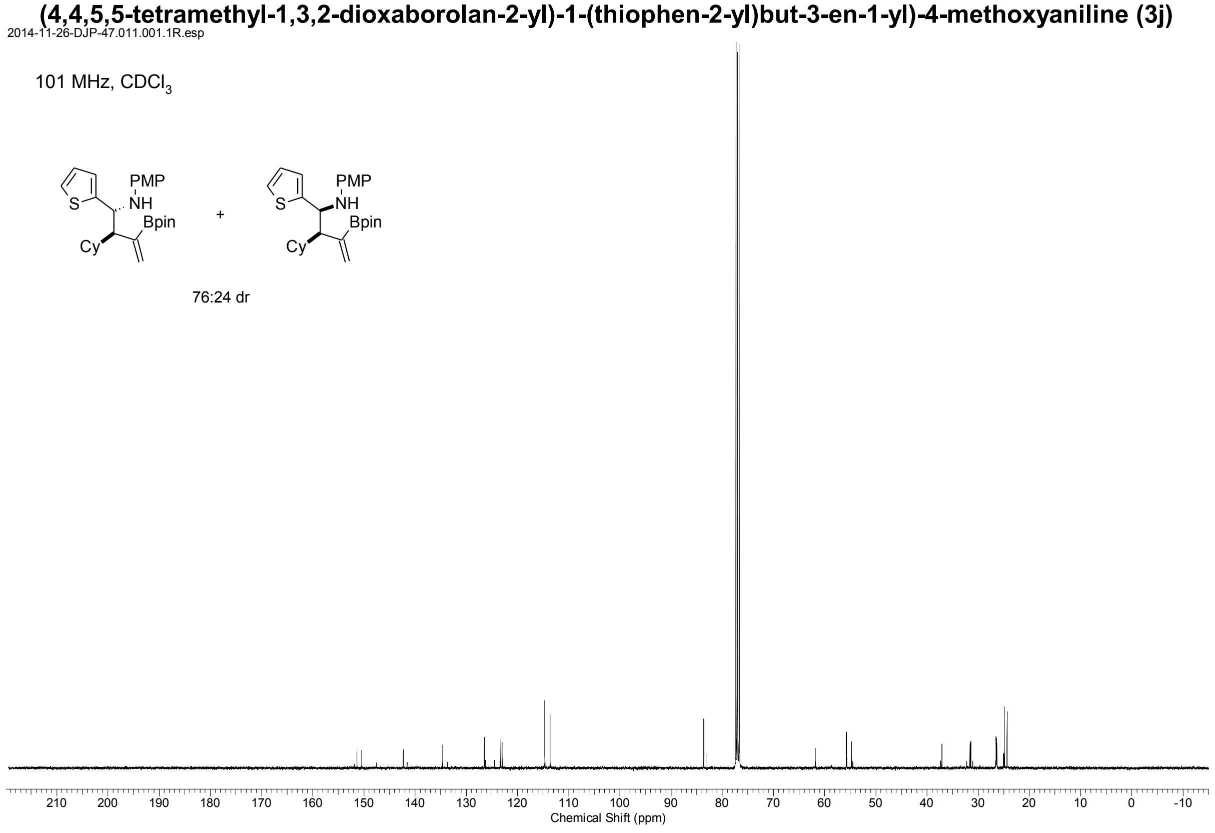

rac-*N*-((1*R*,2*R*)-2-Cyclohexyl-1-(naphthalen-1-yl)-3-(4,4,5,5-tetramethyl-1,3,2-dioxaborolan-2-yl)but-3-en-1-yl)-4-methoxyaniline (3k)

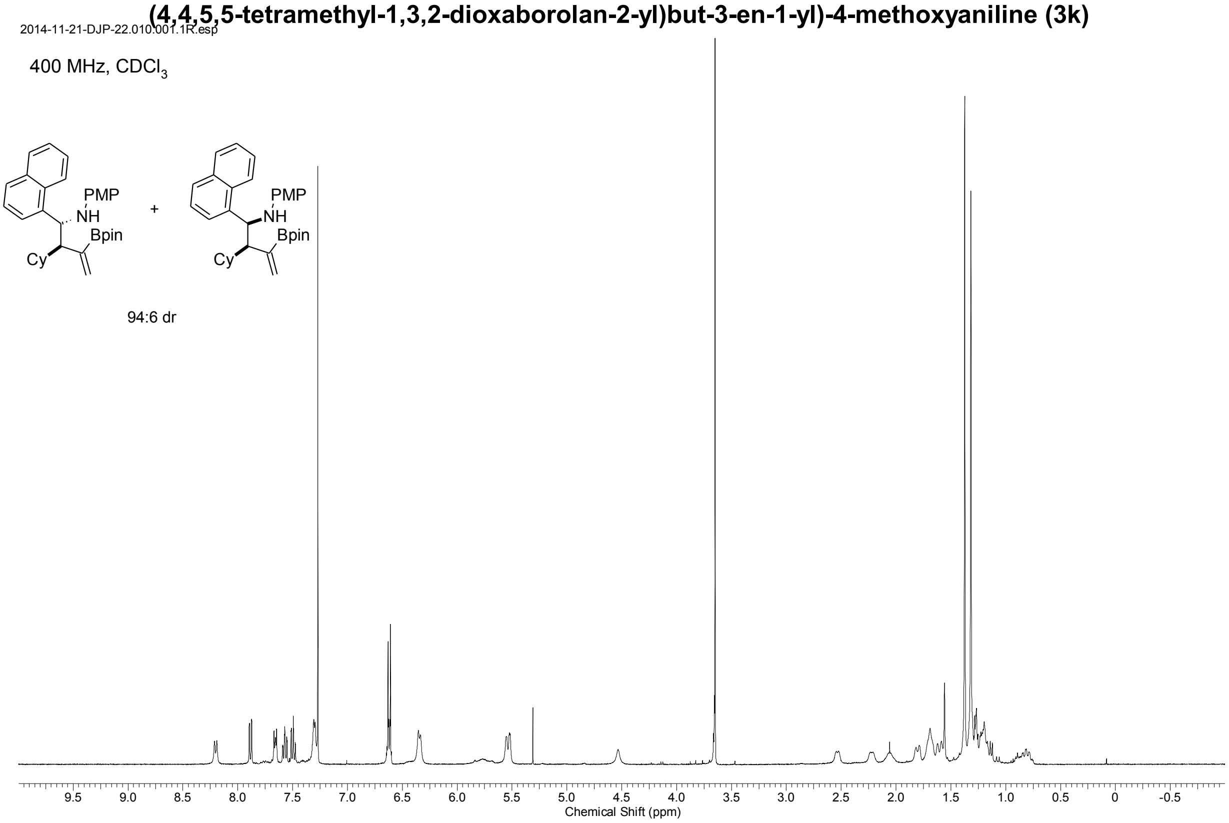

rac-*N*-((1*R*,2*R*)-2-Cyclohexyl-1-(naphthalen-1-yl)-3-(4,4,5,5-tetramethyl-1,3,2-dioxaborolan-2-yl)but-3-en-1-yl)-4-methoxyaniline (3k)

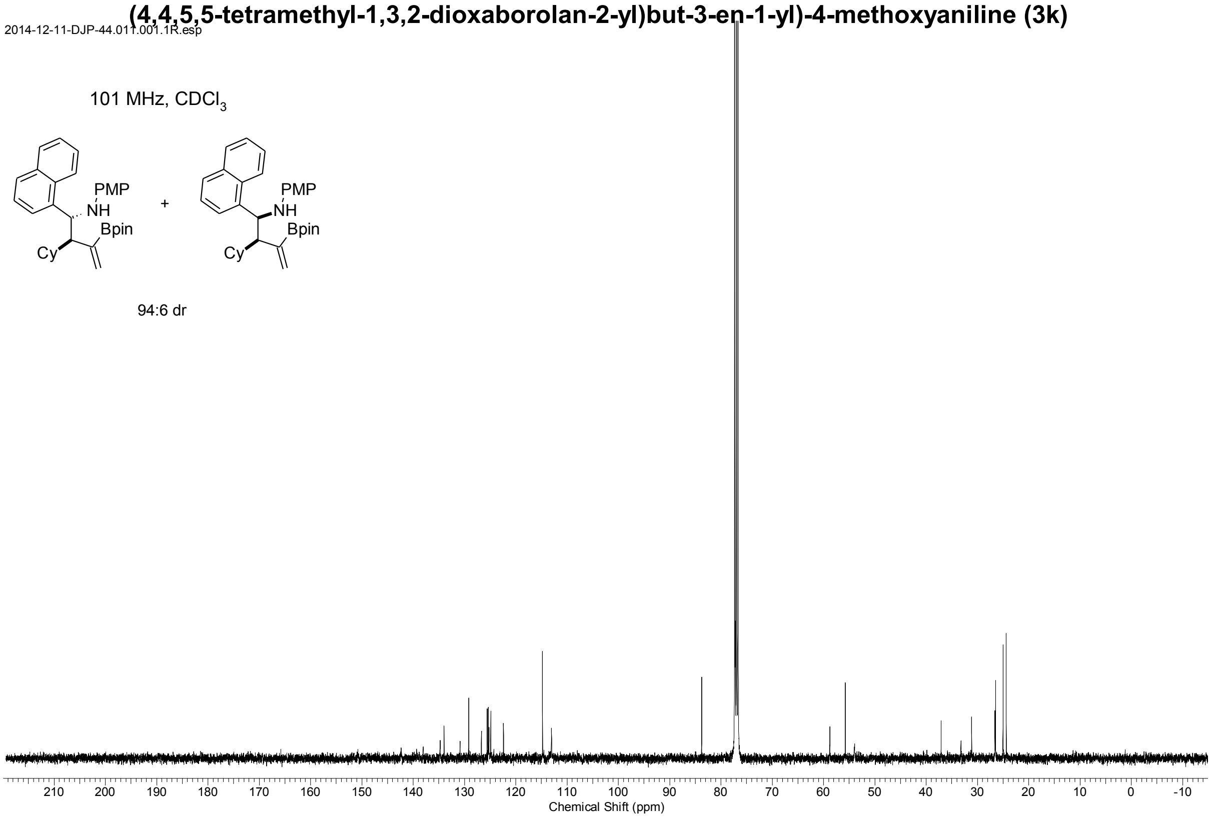

***rac-N*-((1*R*,2*R*)-2-Cyclohexyl-3-(4,4,5,5-tetramethyl-1,3,2-dioxaborolan-2-yl)-1-(*o*-tolyl)but-3-en-1-yl)-4-methoxyaniline (3m)**

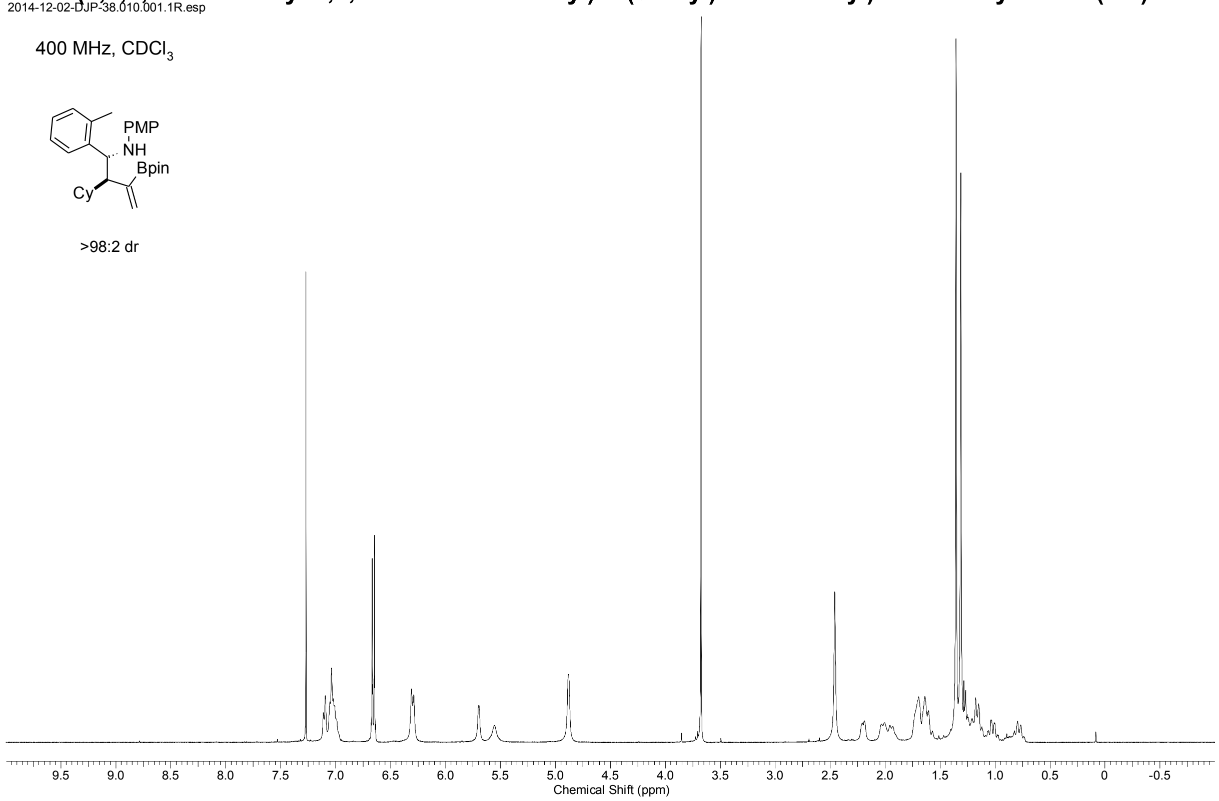

***rac-N*-((1*R*,2*R*)-2-Cyclohexyl-3-(4,4,5,5-tetramethyl-1,3,2-dioxaborolan-2-yl)-1-(*o*-tolyl)but-3-en-1-yl)-4-methoxyaniline (3m)**

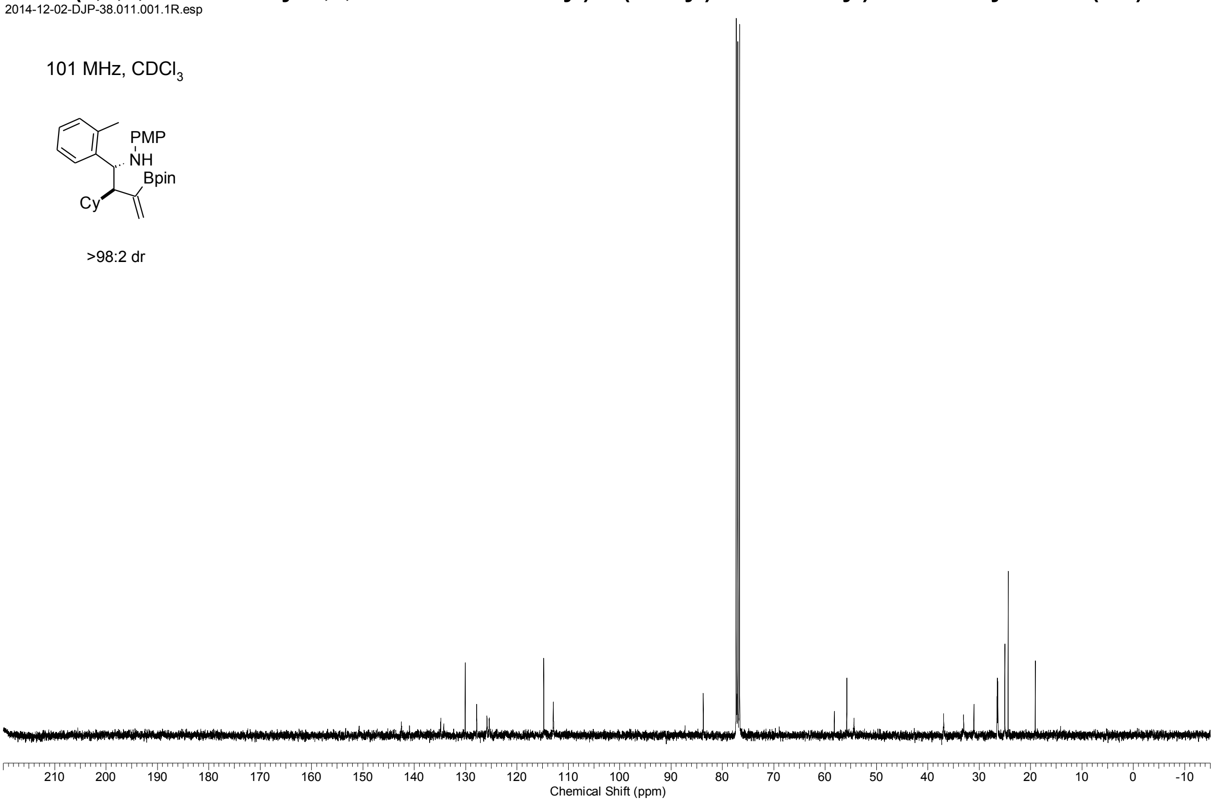

rac-***N***-((3*S*,4*R*)-4-Cyclohexyl-2-methyl-5-(4,4,5,5-tetramethyl-1,3,2-dioxaborolan-2-yl)hex-5-en-3-yl)-4-methoxyaniline (3n)

2015-02-12-DJP-6.020.001.1R.esp

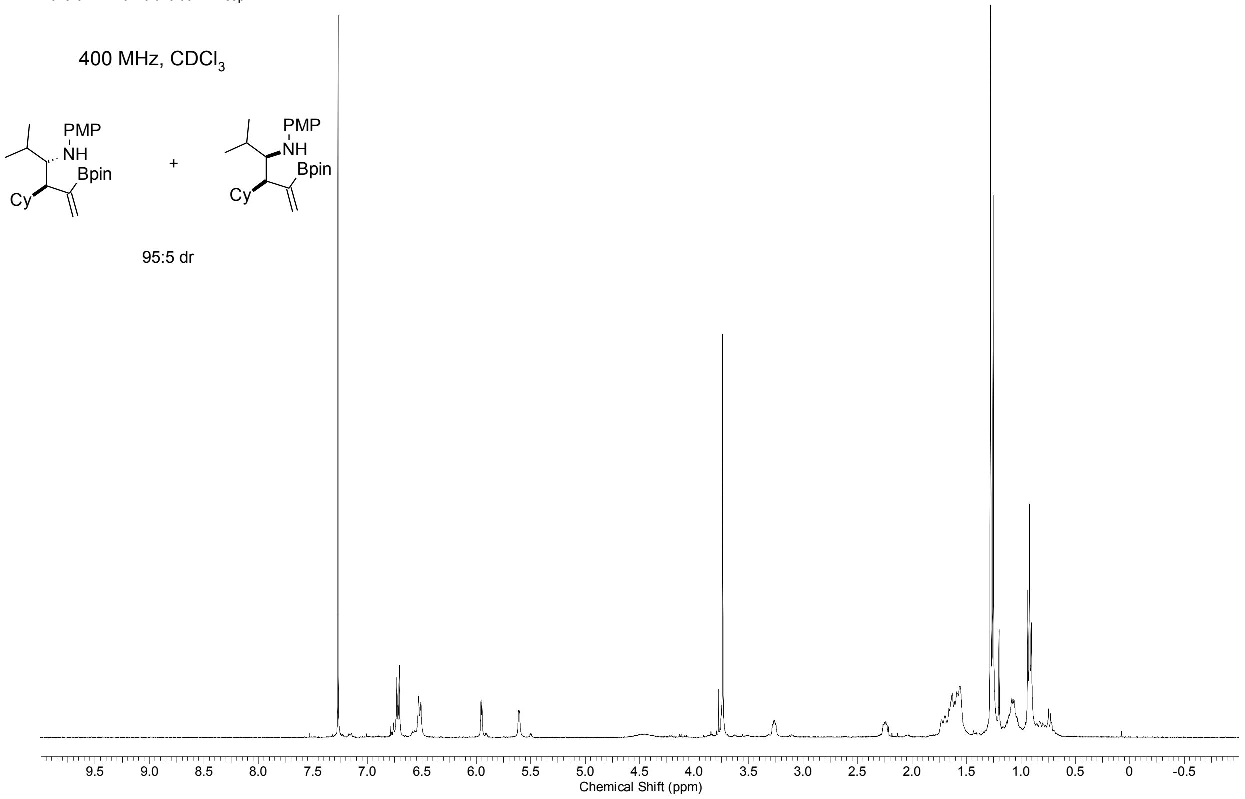

rac-***N***-((3*S*,4*R*)-4-Cyclohexyl-2-methyl-5-(4,4,5,5-tetramethyl-1,3,2-dioxaborolan-2-yl)hex-5-en-3-yl)-4-methoxyaniline (3n)

2015-02-23-DJP-55.011.001.1R.esp

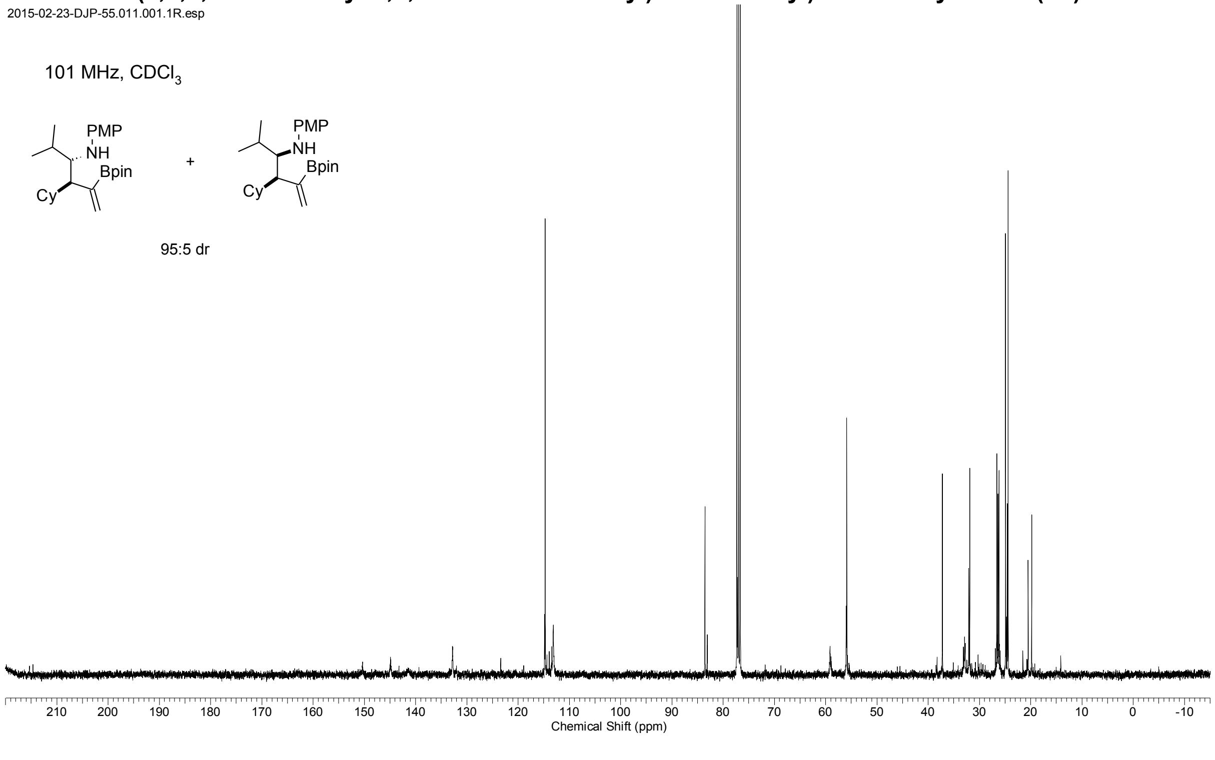

rac-N-((3*R*,4*R*)-4-Cyclohexyl-2,2-dimethyl-5-(4,4,5,5-tetramethyl-1,3,2-dioxaborolan-2-yl)hex-5-en-3-yl)-4-methoxyaniline (3o)

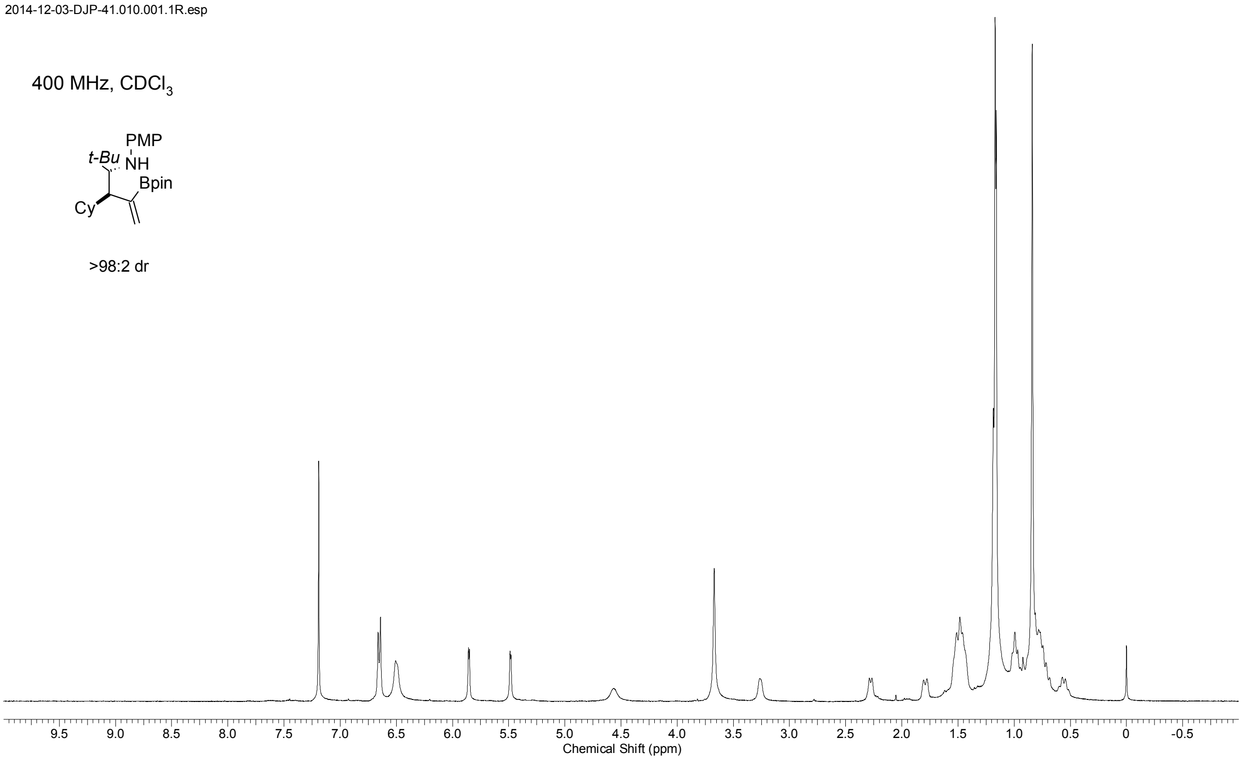

rac-N-((3*R*,4*R*)-4-Cyclohexyl-2,2-dimethyl-5-(4,4,5,5-tetramethyl-1,3,2-dioxaborolan-2-yl)hex-5-en-3-yl)-4-methoxyaniline (3o)

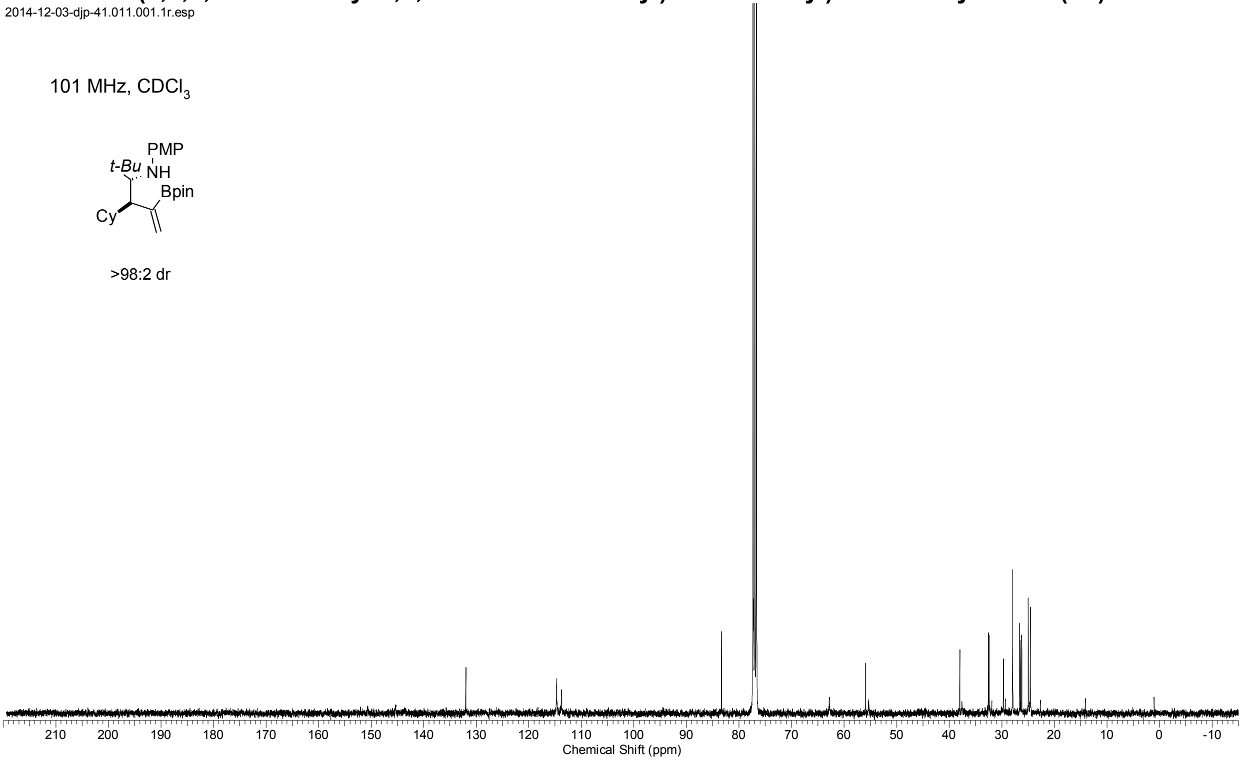

4-Methoxy-*N*-(1-(4-methoxyphenyl)-2,2-dimethyl-3-(4,4,5,5-tetramethyl-1,3,2-dioxaborolan-2-yl)but-3-en-1-yl)aniline (3p)

2015-03-23-DJP-20.010.001.1R.esp

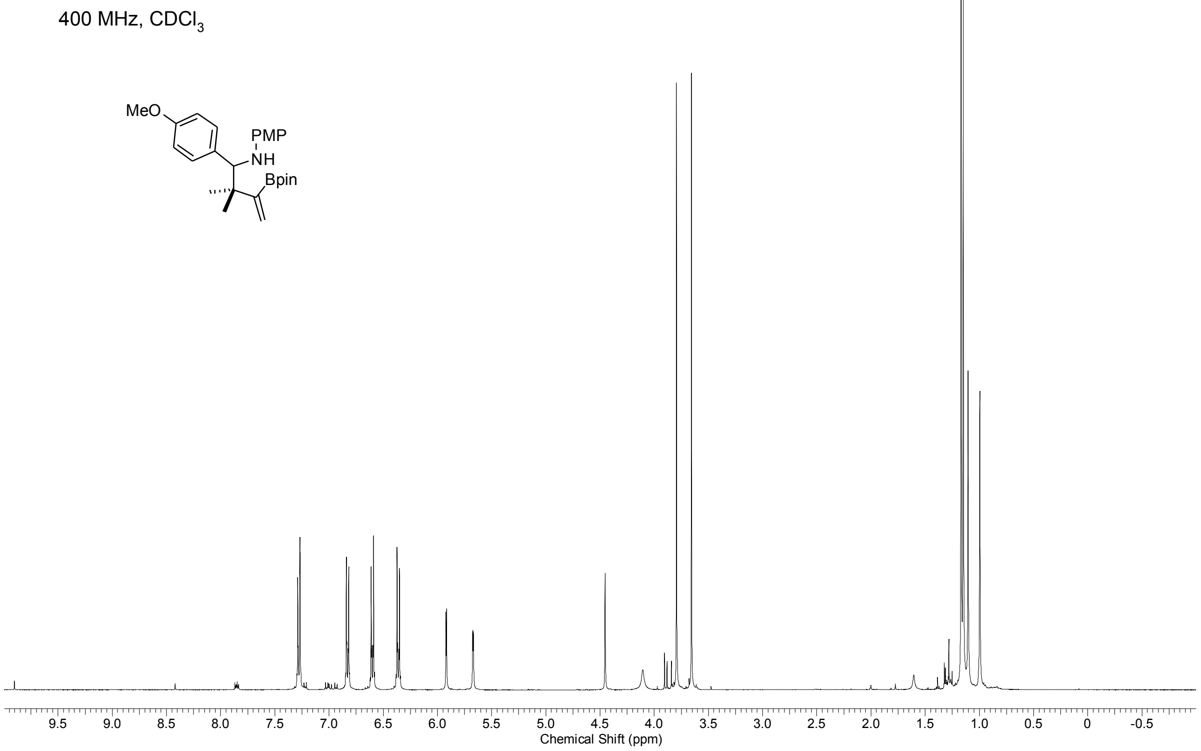

4-Methoxy-*N*-(1-(4-methoxyphenyl)-2,2-dimethyl-3-(4,4,5,5-tetramethyl-1,3,2-dioxaborolan-2-yl)but-3-en-1-yl)aniline (3p)

2015-03-23-DJP-20.011.001.1R.esp

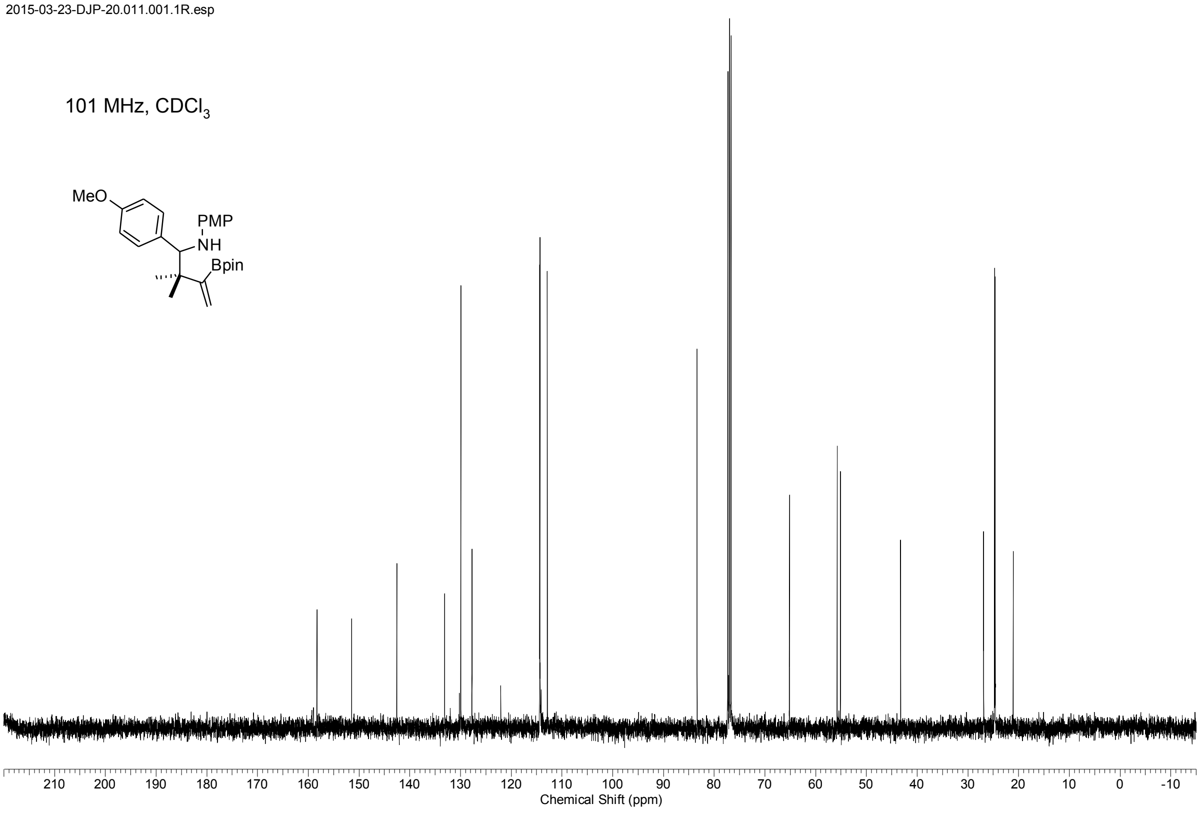

N-(1-(4-Bromophenyl)-2,2-dimethyl-3-(4,4,5,5-tetramethyl-1,3,2-dioxaborolan-2-yl)but-3-en-1-yl)-4-methoxyaniline (3q)

2015-03-23-DJP-19.010.001.1R.esp

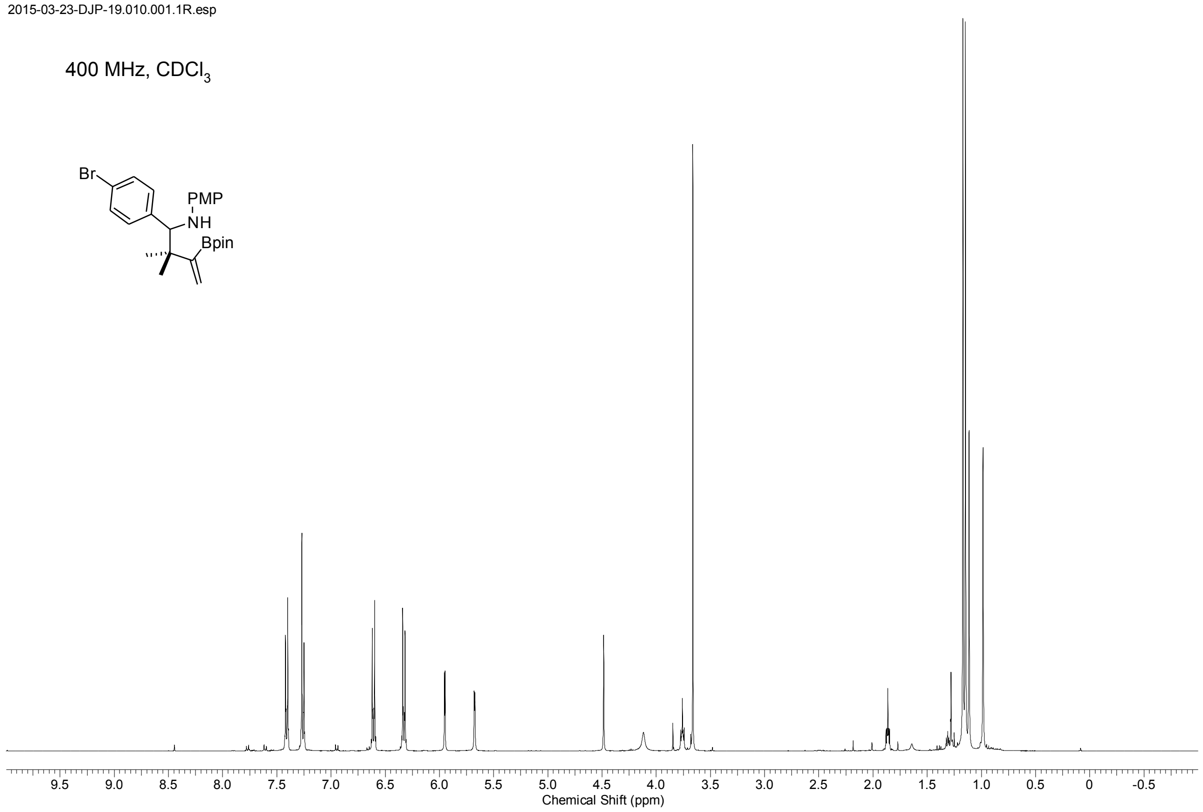

N-(1-(4-Bromophenyl)-2,2-dimethyl-3-(4,4,5,5-tetramethyl-1,3,2-dioxaborolan-2-yl)but-3-en-1-yl)-4-methoxyaniline (3q)

2015-03-23-DJP-19.011.001.1R.esp

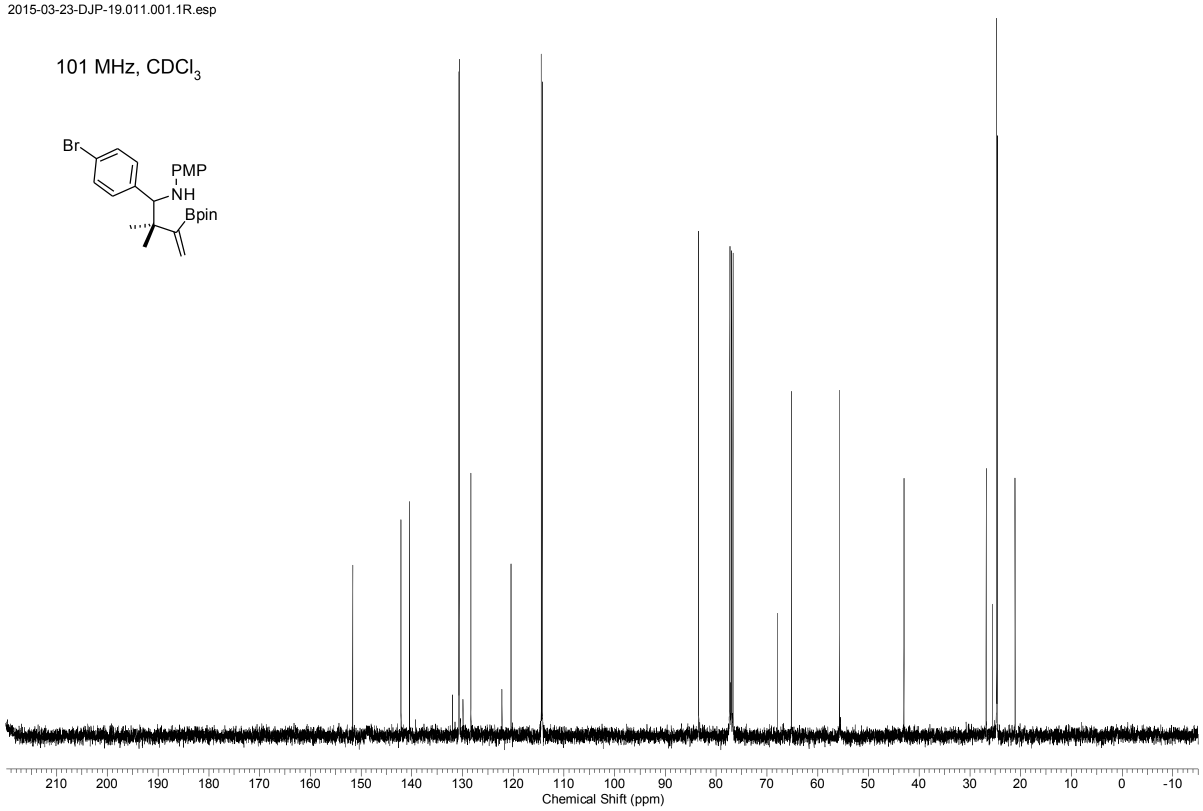

N-(2,2-Dimethyl-1-phenyl-3-(4,4,5,5-tetramethyl-1,3,2-dioxaborolan-2-yl)but-3-en-1-yl)aniline (3r)

2015-02-27-DJP-55.020.001.1R.esp

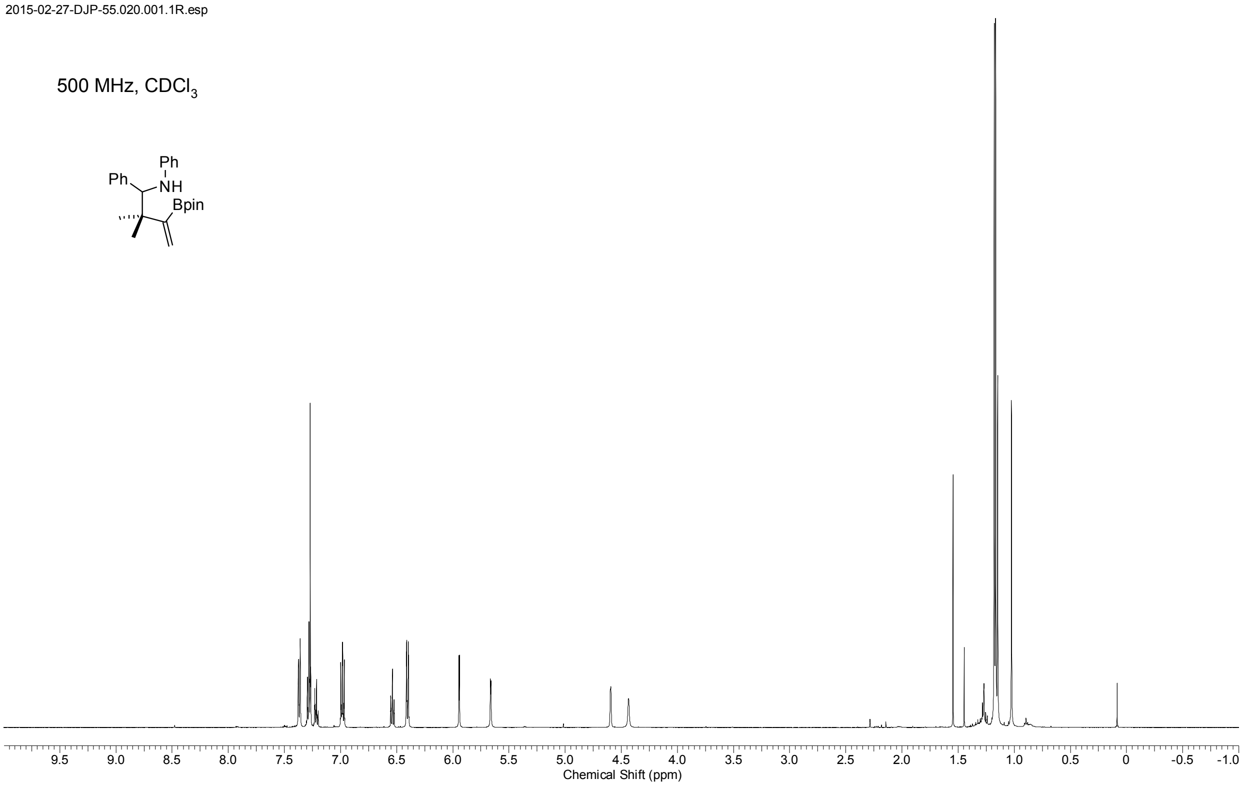

N-(2,2-Dimethyl-1-phenyl-3-(4,4,5,5-tetramethyl-1,3,2-dioxaborolan-2-yl)but-3-en-1-yl)aniline (3r)

2015-02-27-DJP-55.022.001.1R.esp

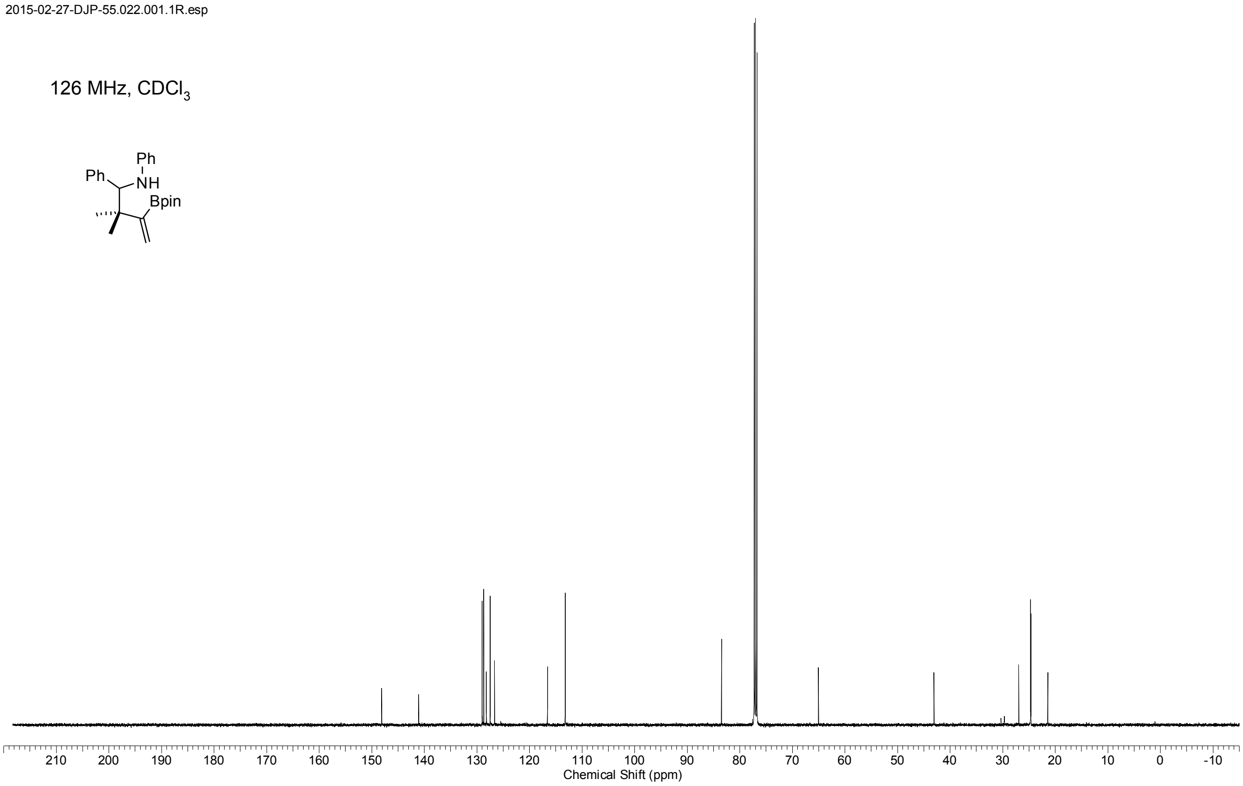

N-(Furan-2-yl(1-(1-(4,4,5,5-tetramethyl-1,3,2-dioxaborolan-2-yl)vinyl)cyclohexyl)methyl)-4-methoxyaniline (3s)

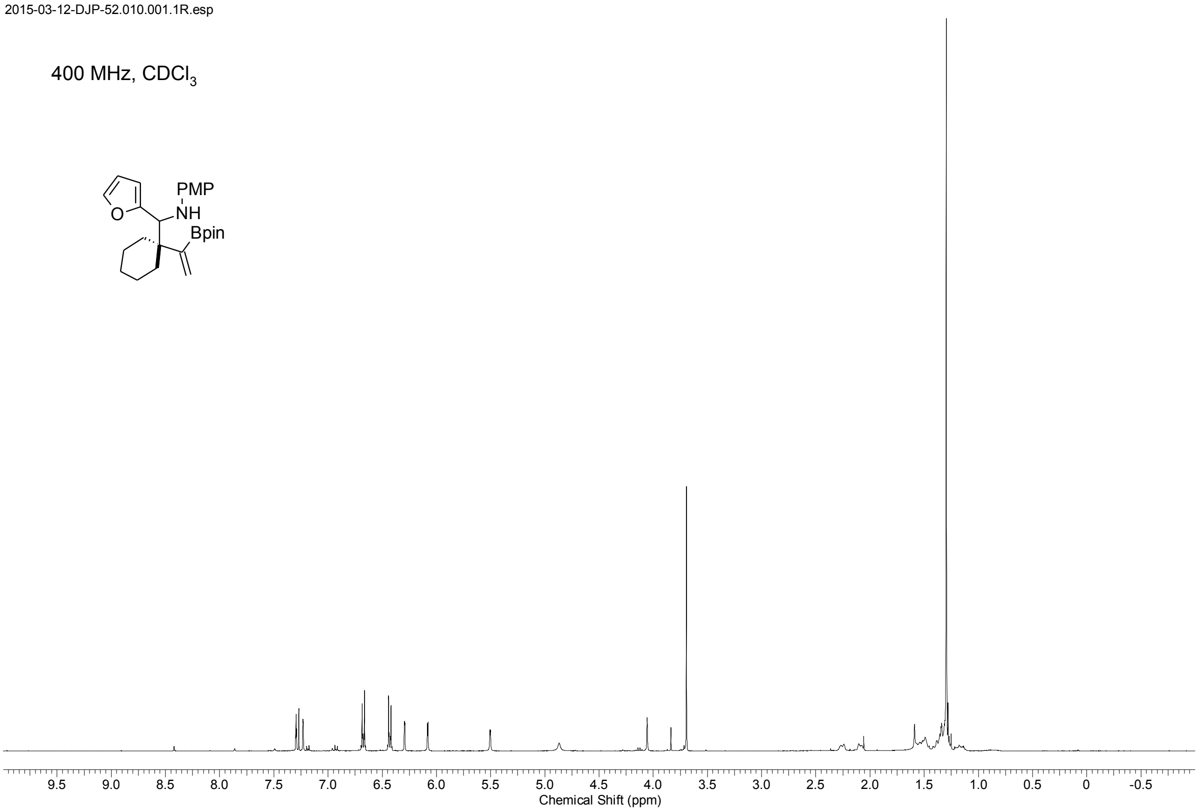

N-(Furan-2-yl(1-(1-(4,4,5,5-tetramethyl-1,3,2-dioxaborolan-2-yl)vinyl)cyclohexyl)methyl)-4-methoxyaniline (3s)

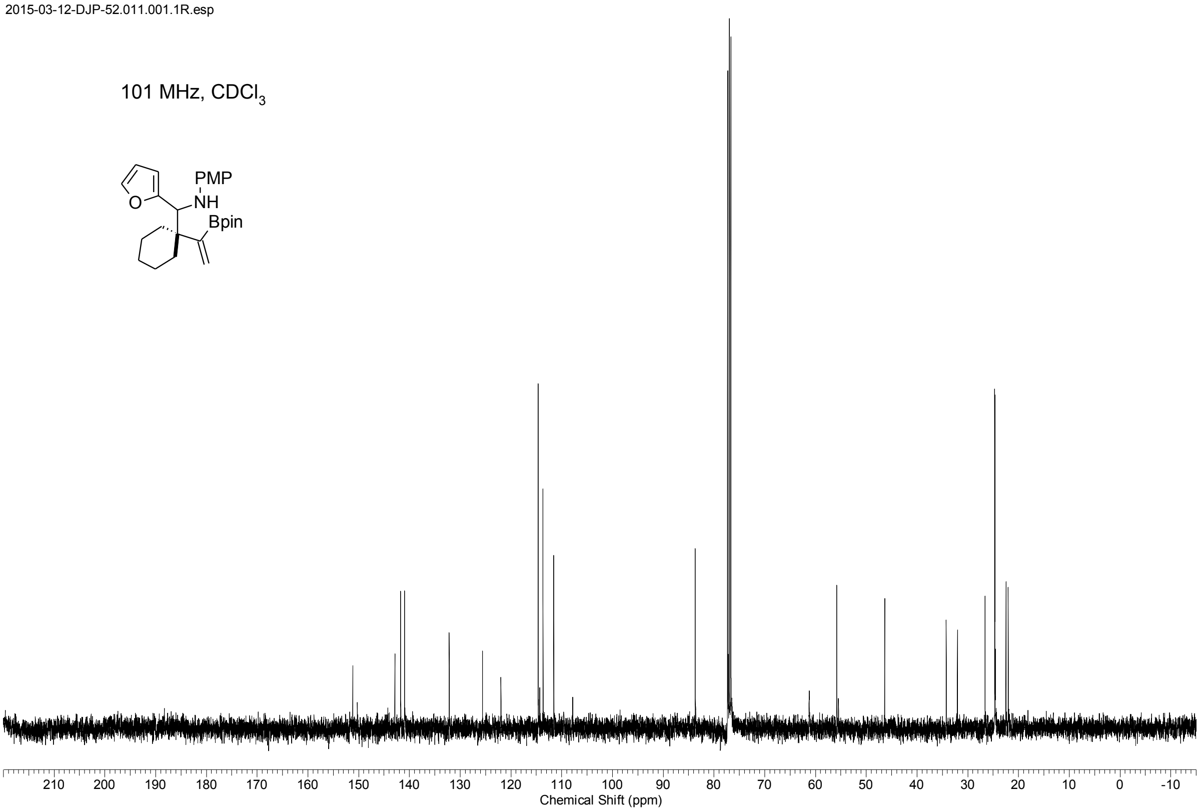

4-Methoxy-*N*-((1-(1-(4,4,5,5-tetramethyl-1,3,2-dioxaborolan-2-yl)vinyl)cyclohexyl)(thiophen-2-yl)methyl)aniline (3t)

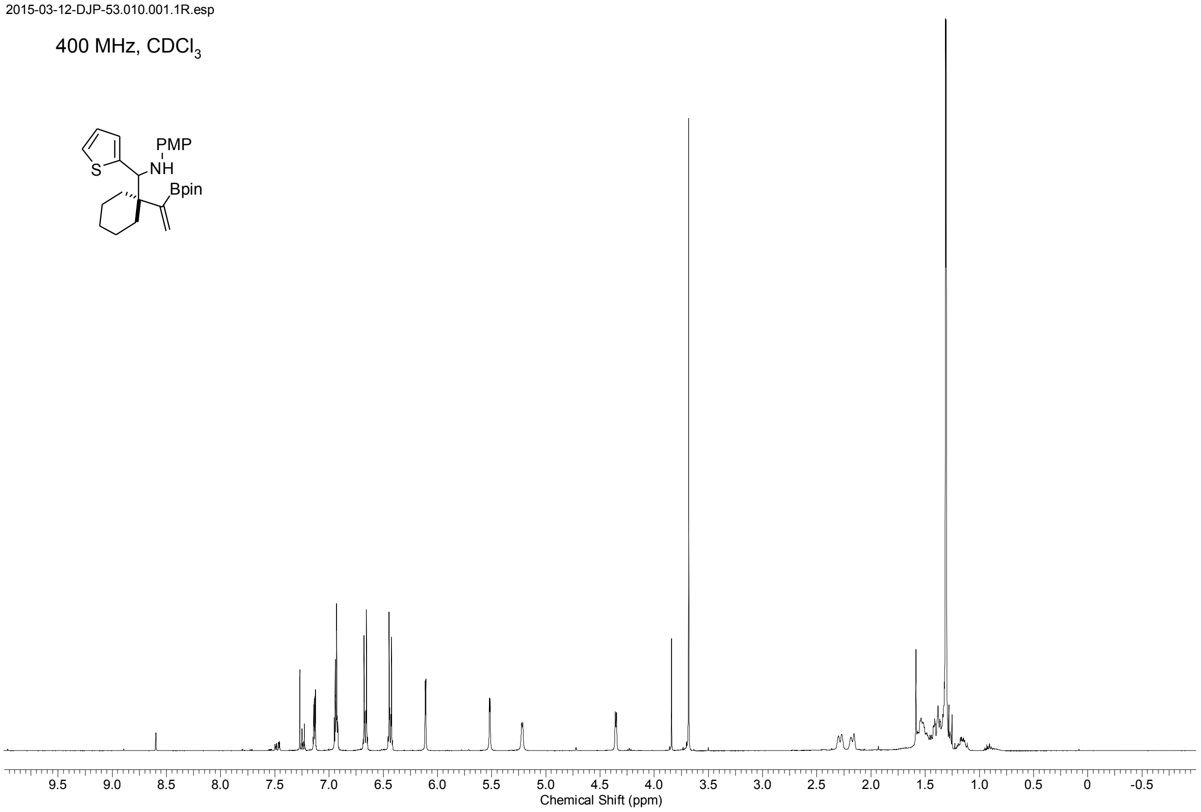

4-Methoxy-*N*-((1-(1-(4,4,5,5-tetramethyl-1,3,2-dioxaborolan-2-yl)vinyl)cyclohexyl)(thiophen-2-yl)methyl)aniline (3t)

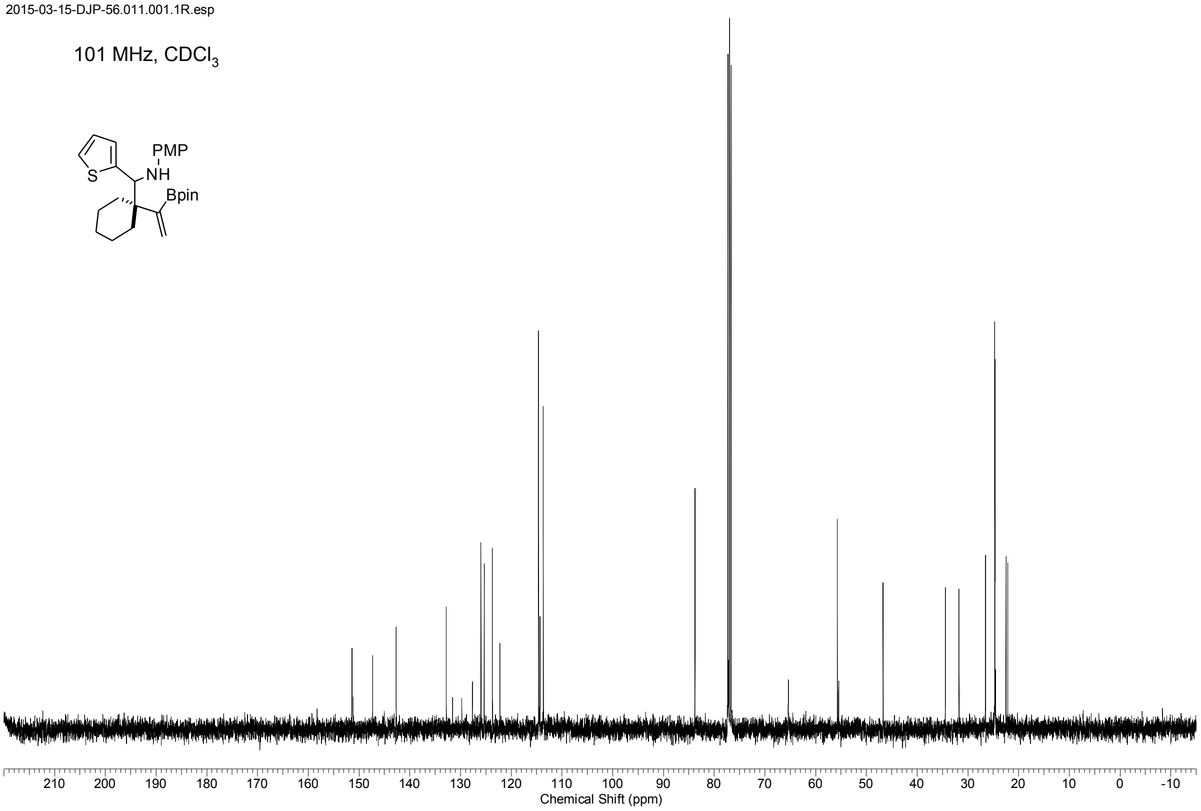

rac-4-Methoxy-*N*-((1*R*,2*R*)-2-(1-(4,4,5,5-tetramethyl-1,3,2-dioxaborolan-2-yl)vinyl)-1-(*o*-tolyl)decyl)aniline (3u)

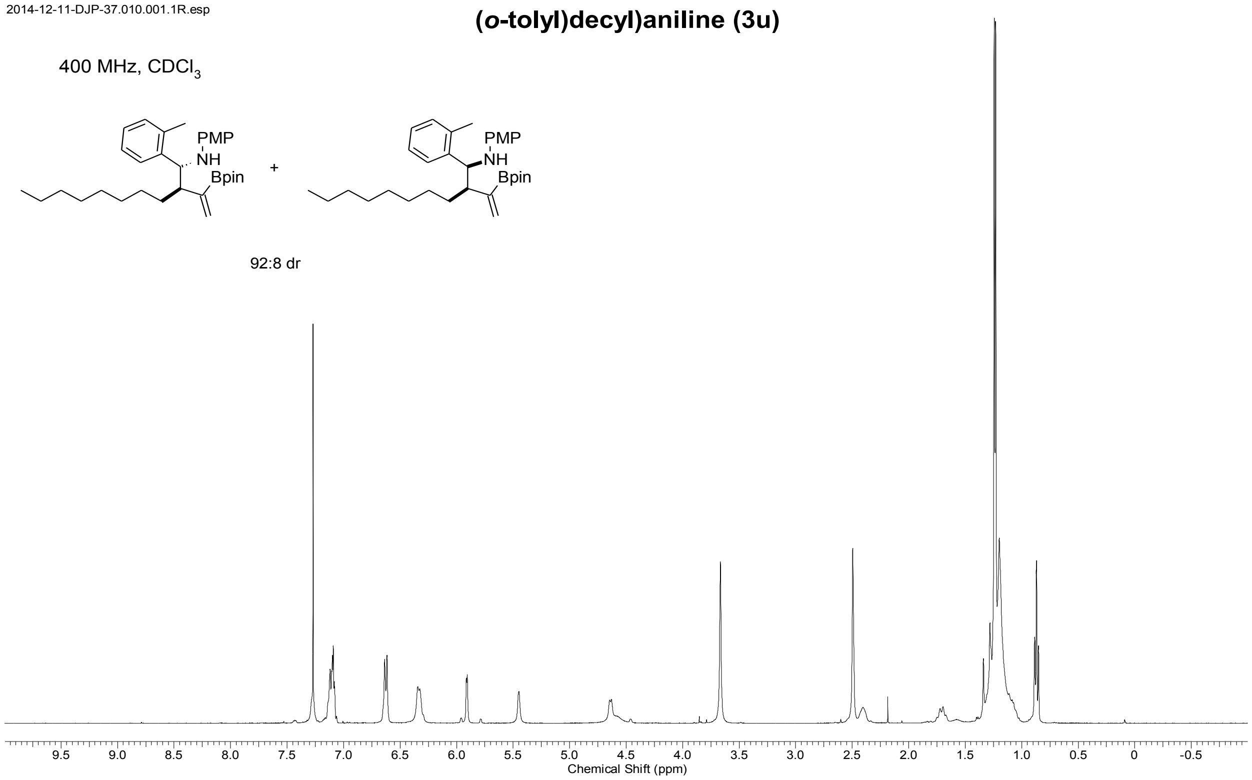

rac-4-Methoxy-*N*-((1*R*,2*R*)-2-(1-(4,4,5,5-tetramethyl-1,3,2-dioxaborolan-2-yl)vinyl)-1-(*o*-tolyl)decyl)aniline (3u)

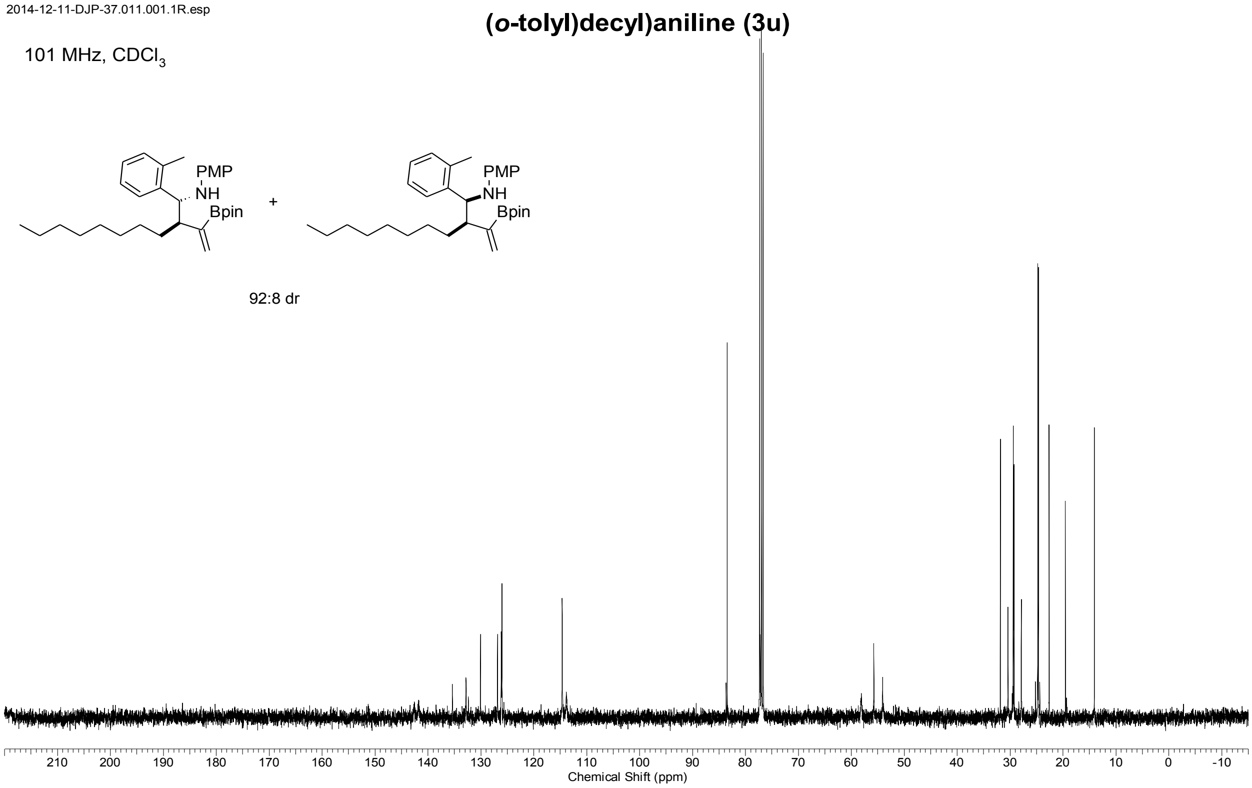

N-(Phenyl(1-(1-(4,4,5,5-tetramethyl-1,3,2-dioxaborolan-2-yl)vinyl)cyclohexyl)methyl)aniline (3v)

2015-03-04-djp-56.012.001.1r.esp

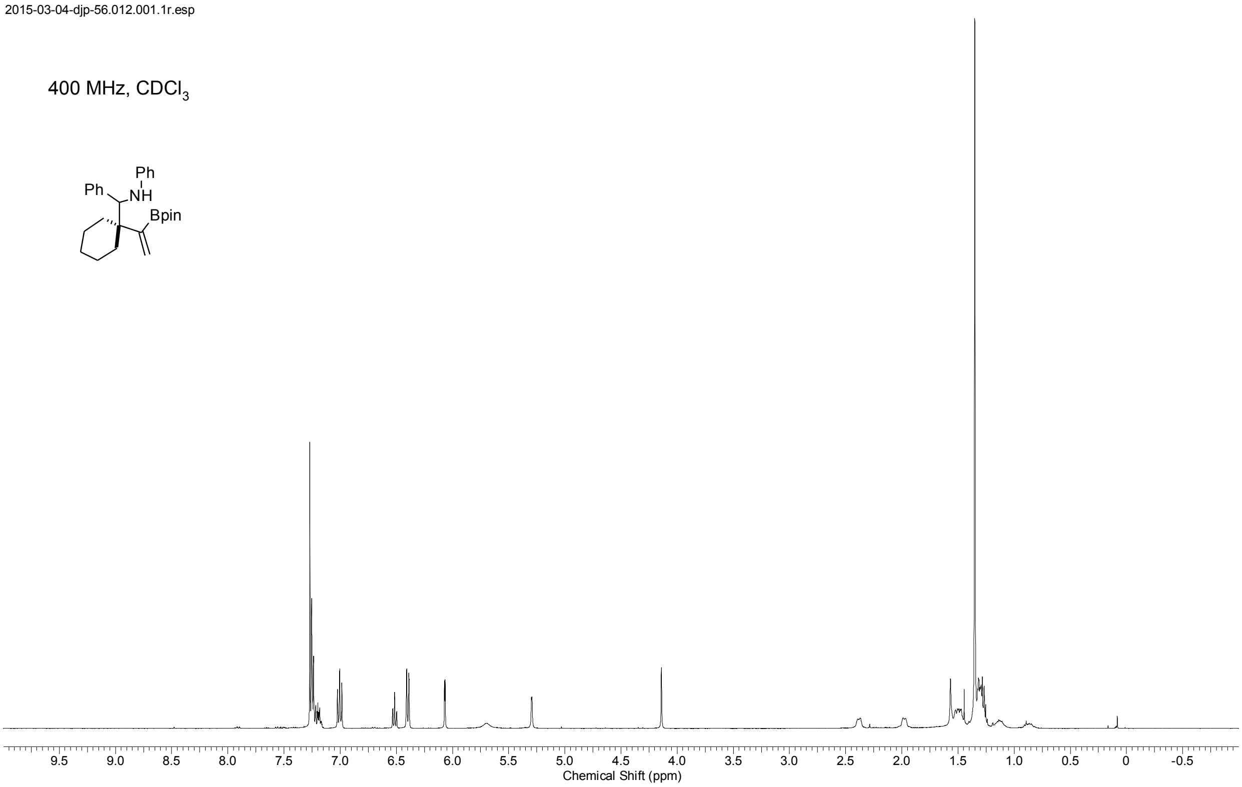

N-(Phenyl(1-(1-(4,4,5,5-tetramethyl-1,3,2-dioxaborolan-2-yl)vinyl)cyclohexyl)methyl)aniline (3v)

2015-03-04-djp-56.014.001.1r.esp

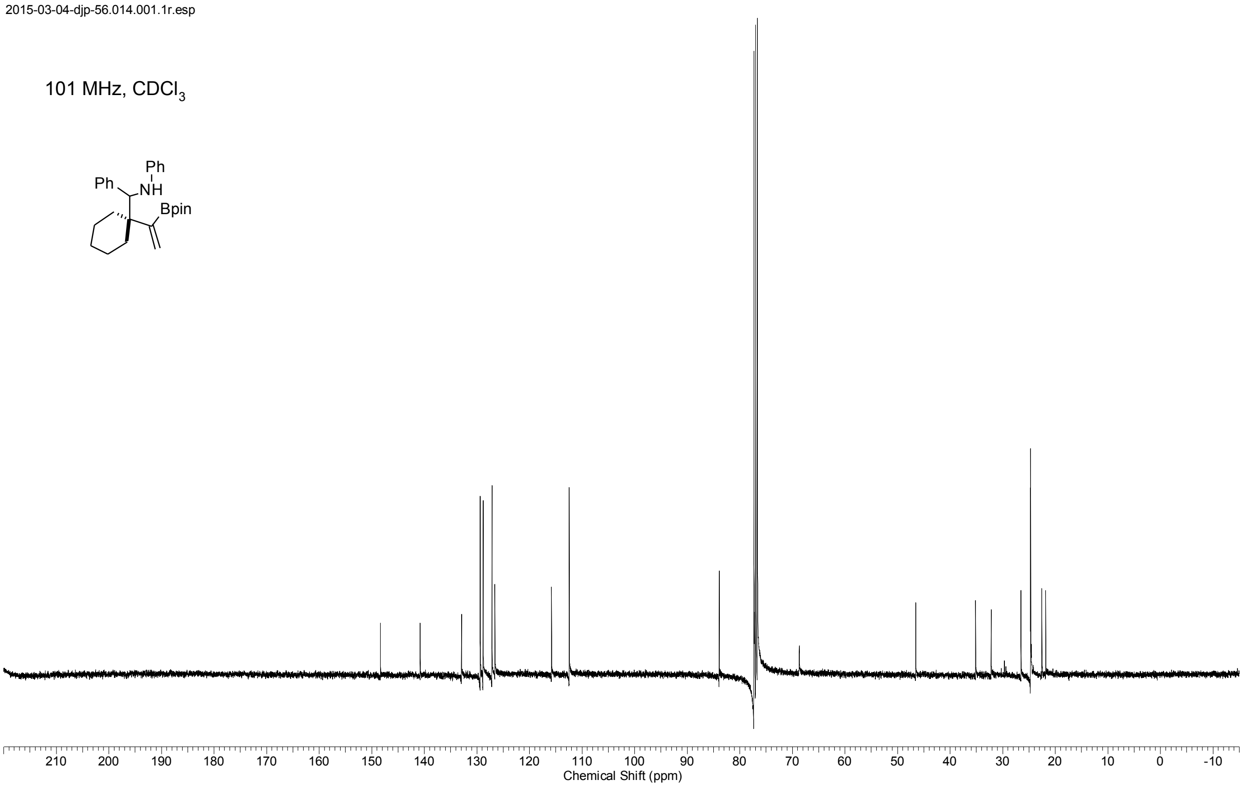

rac-(3*S*,4*S*)-4-(Benzylamino)-3-cyclohexyl-4-(*o*-tolyl)butan-2-one (4a)

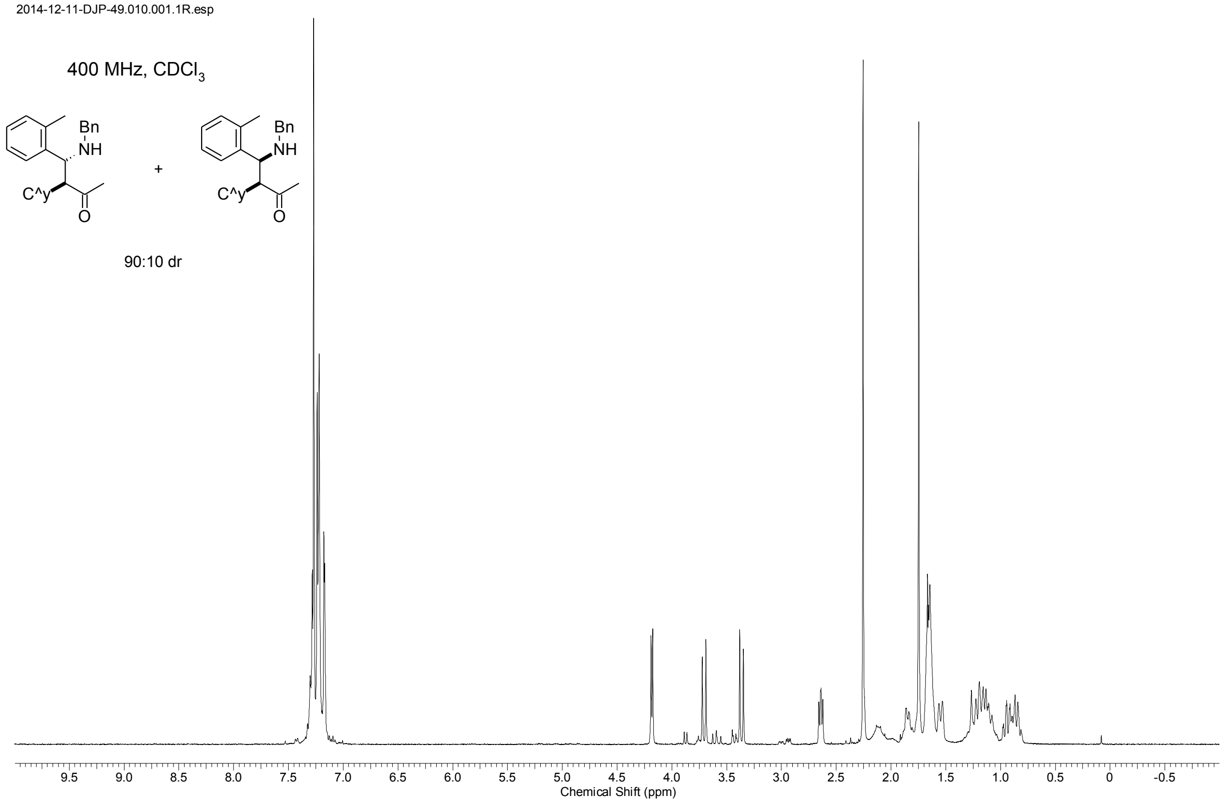

rac-(3*R*,4*S*)-4-(Benzylamino)-3-cyclohexyl-4-(*o*-tolyl)butan-2-one (4a)

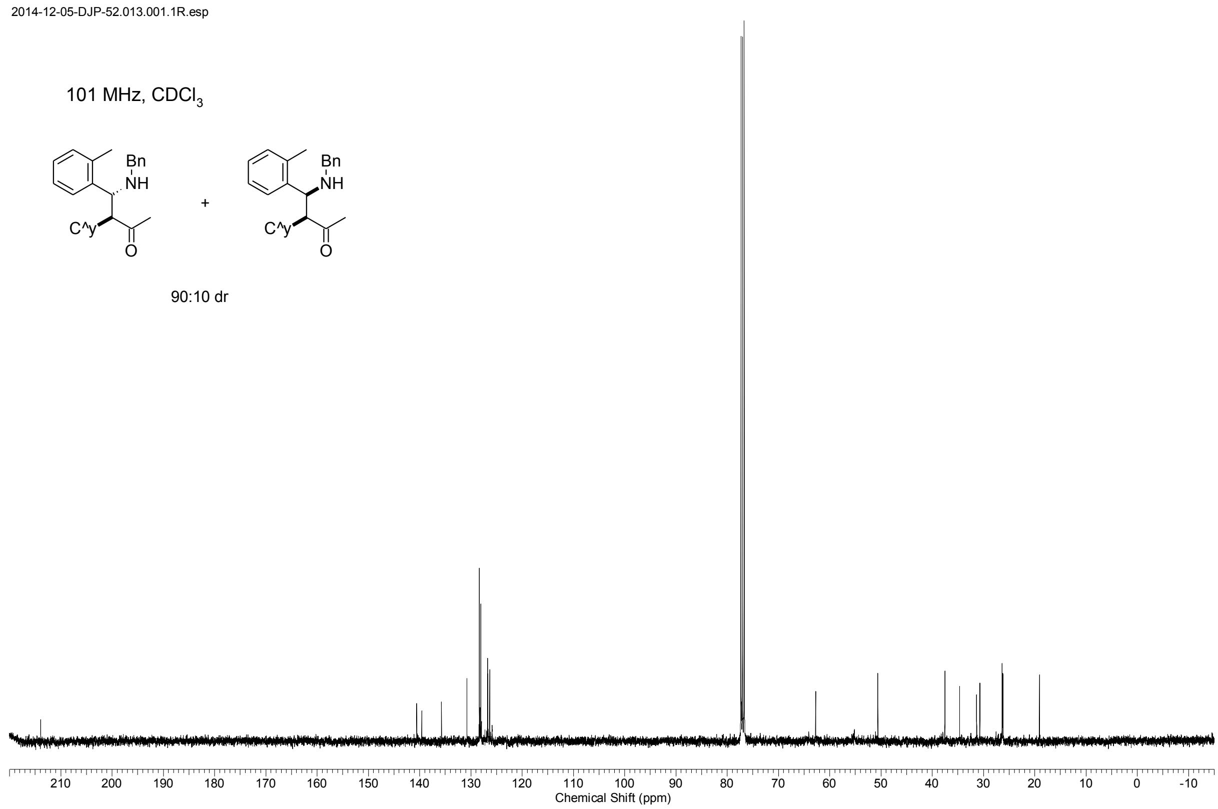

1-(1-((Benzylamino)(phenyl)methyl)cyclohexyl)ethan-1-one (4b)

2015-03-13-djp-48.010.001.1r.esp

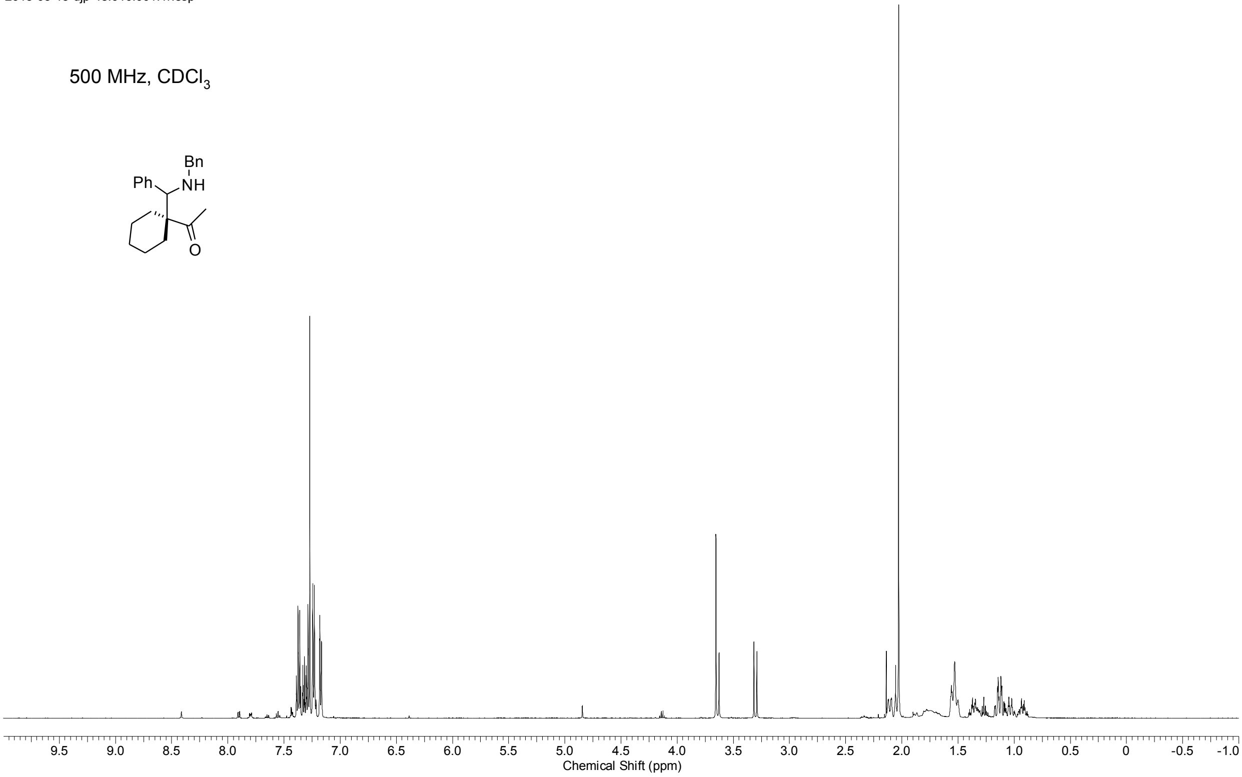

1-(1-((Benzylamino)(phenyl)methyl)cyclohexyl)ethan-1-one (4b)

2015-03-13-djp-48.012.001.1r.esp

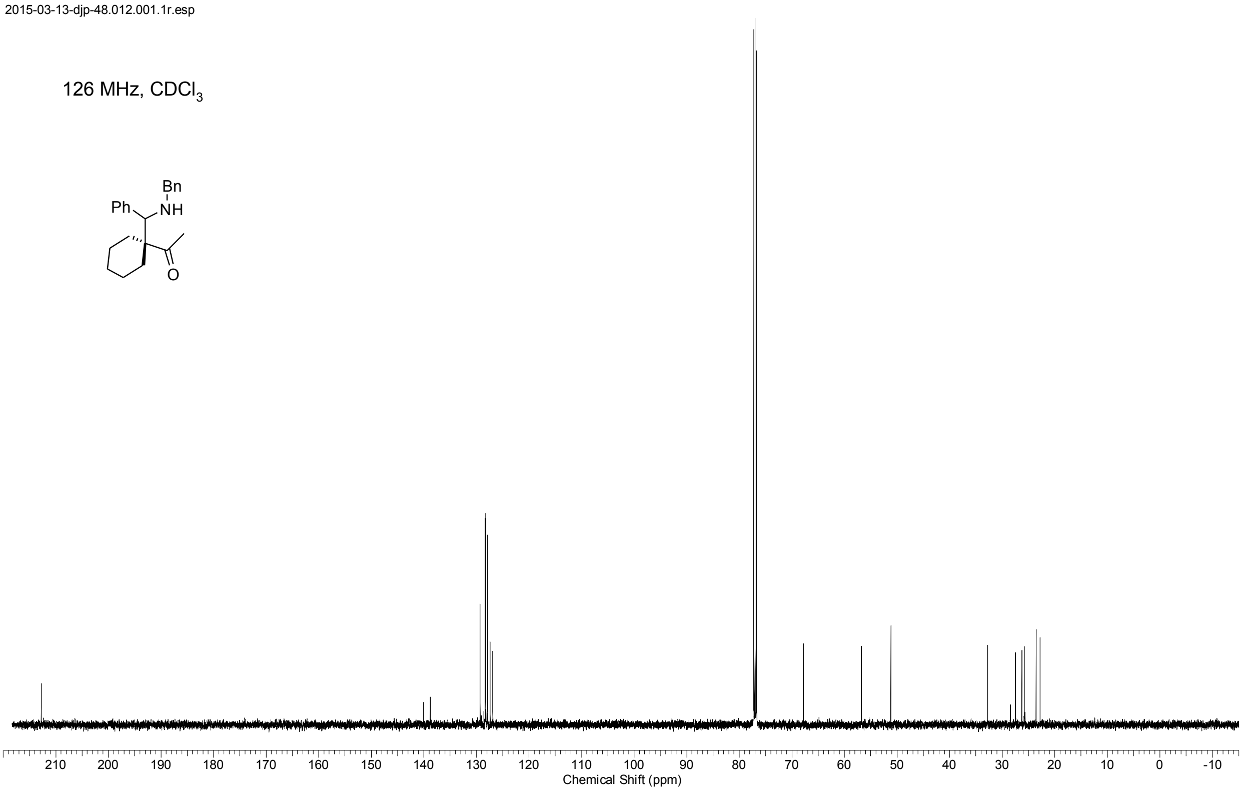

rac-(3*R*,4*S*)-3-Cyclohexyl-4-((4-methoxyphenyl)amino)-4-(*o*-tolyl)butan-2-one (4c)

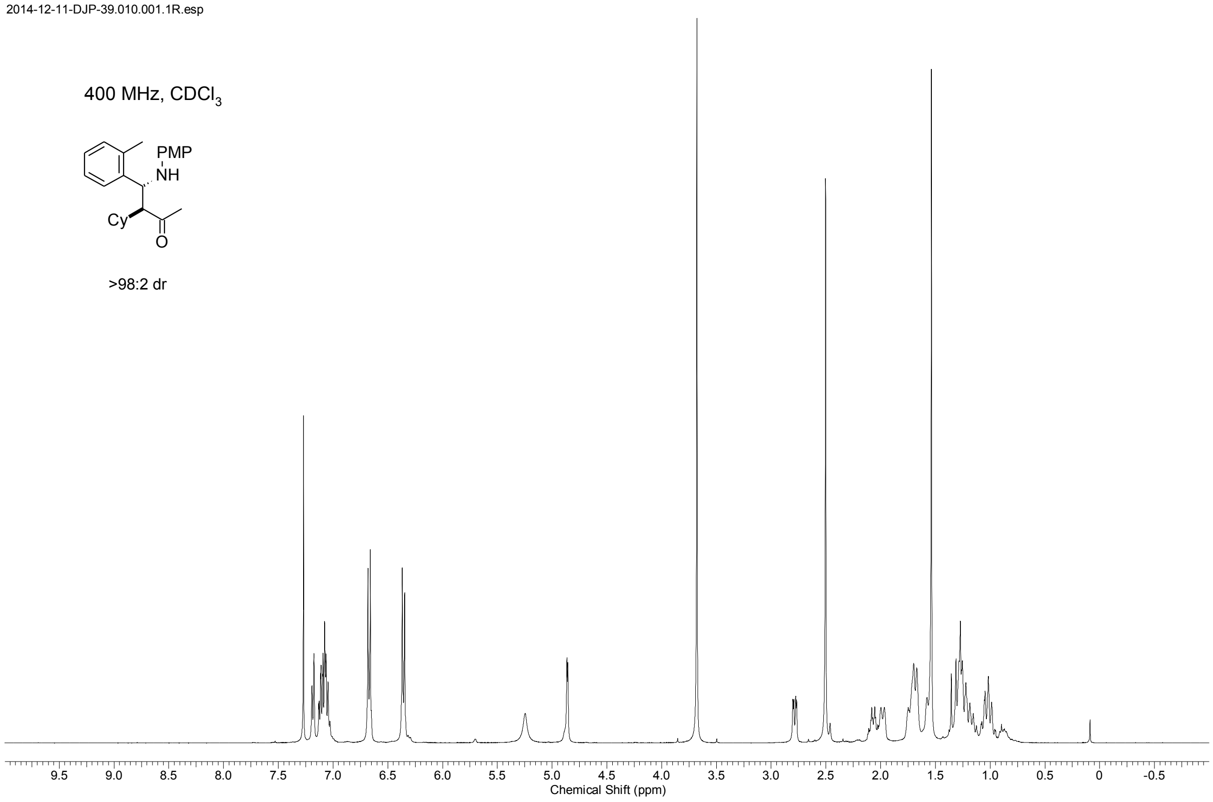

rac-(3*R*,4*S*)-3-Cyclohexyl-4-((4-methoxyphenyl)amino)-4-(*o*-tolyl)butan-2-one (4c)

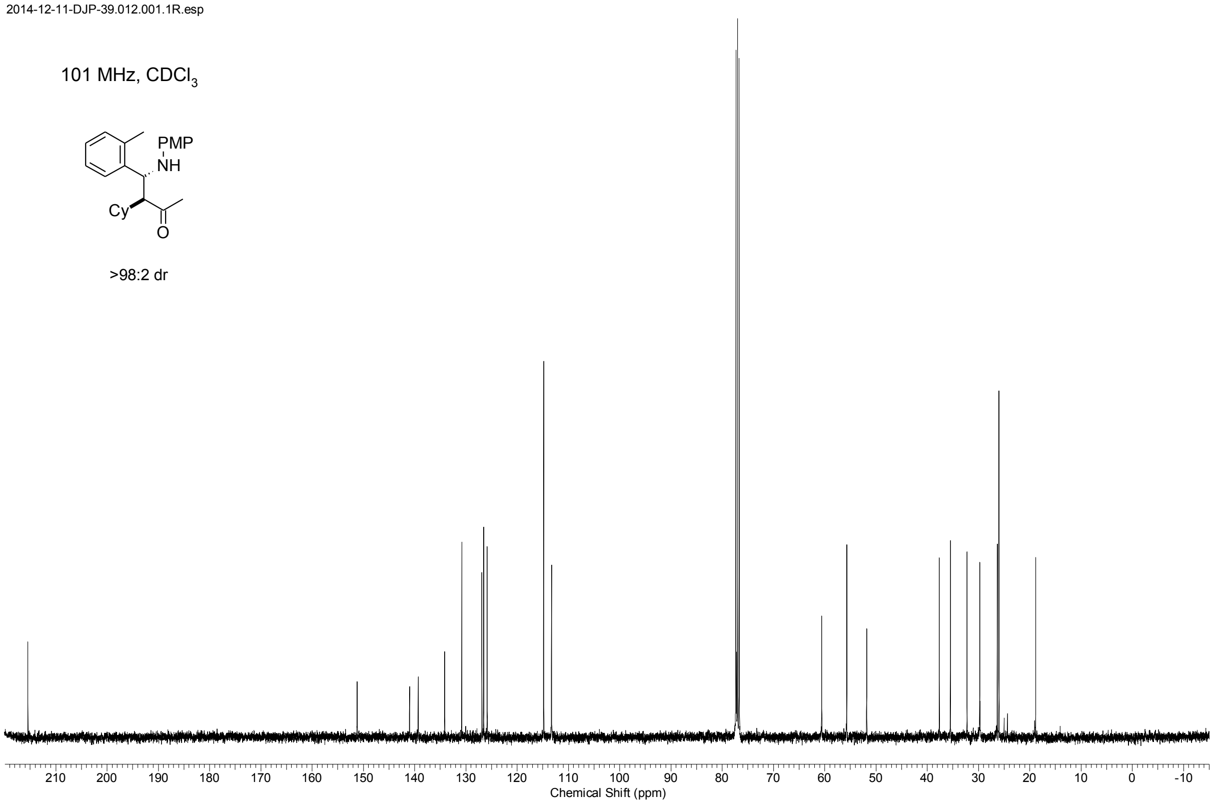

rac-N-((1R,2R)-2-Cyclohexyl-3-(4,4,5,5-tetramethyl-1,3,2-dioxaborolan-2-yl)-1-(o-tolyl)but-3-en-1-yl)-4-methoxybenzenaminium trifluoromethanesulfonate (5a)

2015-03-16-DJP-60.010.001.1R.esp

400 MHz, $CDCl_3$

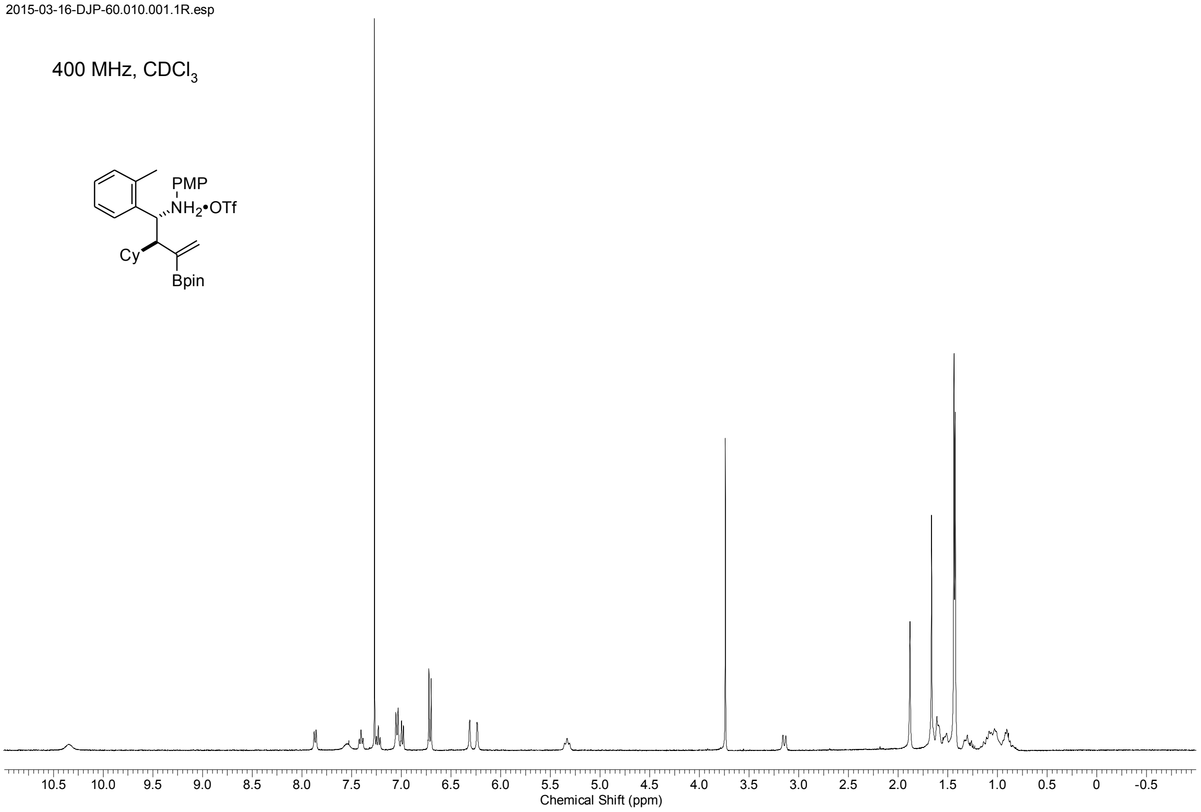

rac-N-((1R,2R)-2-Cyclohexyl-3-(4,4,5,5-tetramethyl-1,3,2-dioxaborolan-2-yl)-1-(o-tolyl)but-3-en-1-yl)-4-methoxybenzenaminium trifluoromethanesulfonate (5a)

2015-03-17-DJP-10.011.001.1R.esp

126 MHz, $CDCl_3$

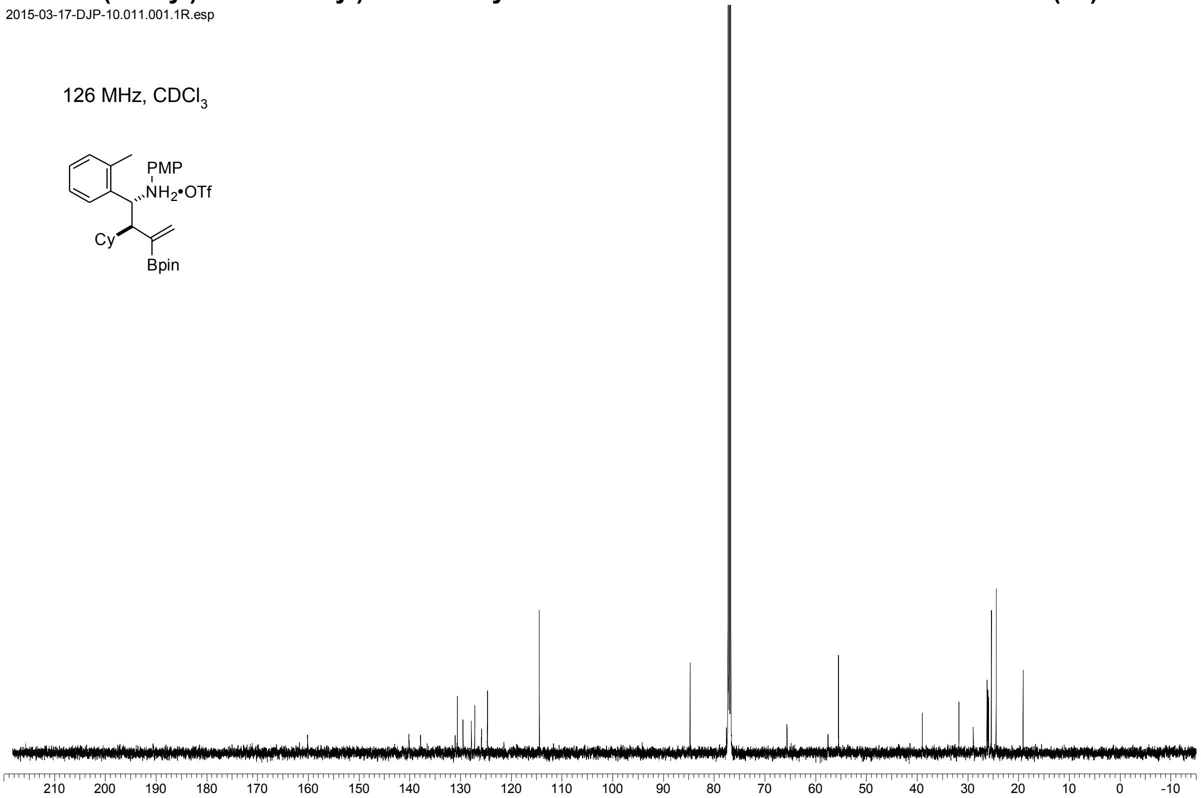

N-(2,2-Dimethyl-1-phenyl-3-(4,4,5,5-tetramethyl-1,3,2-dioxaborolan-2-yl)but-3-en-1-yl)-4-methylbenzenesulfonamide (3w)

2015-05-08-djp-16.020.001.1r.esp

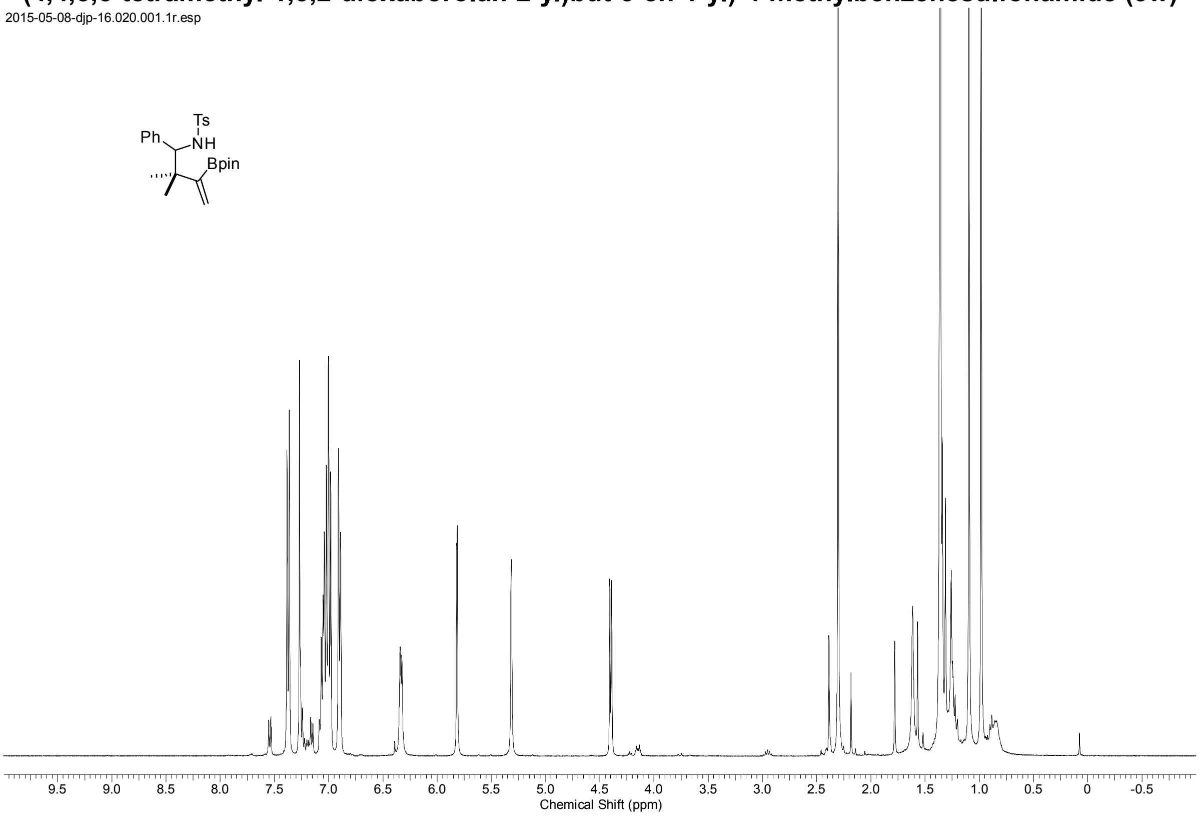

N-(2,2-Dimethyl-1-phenyl-3-(4,4,5,5-tetramethyl-1,3,2-dioxaborolan-2-yl)but-3-en-1-yl)-4-methylbenzenesulfonamide (3w)

2015-05-08-djp-16.021.001.1r.esp

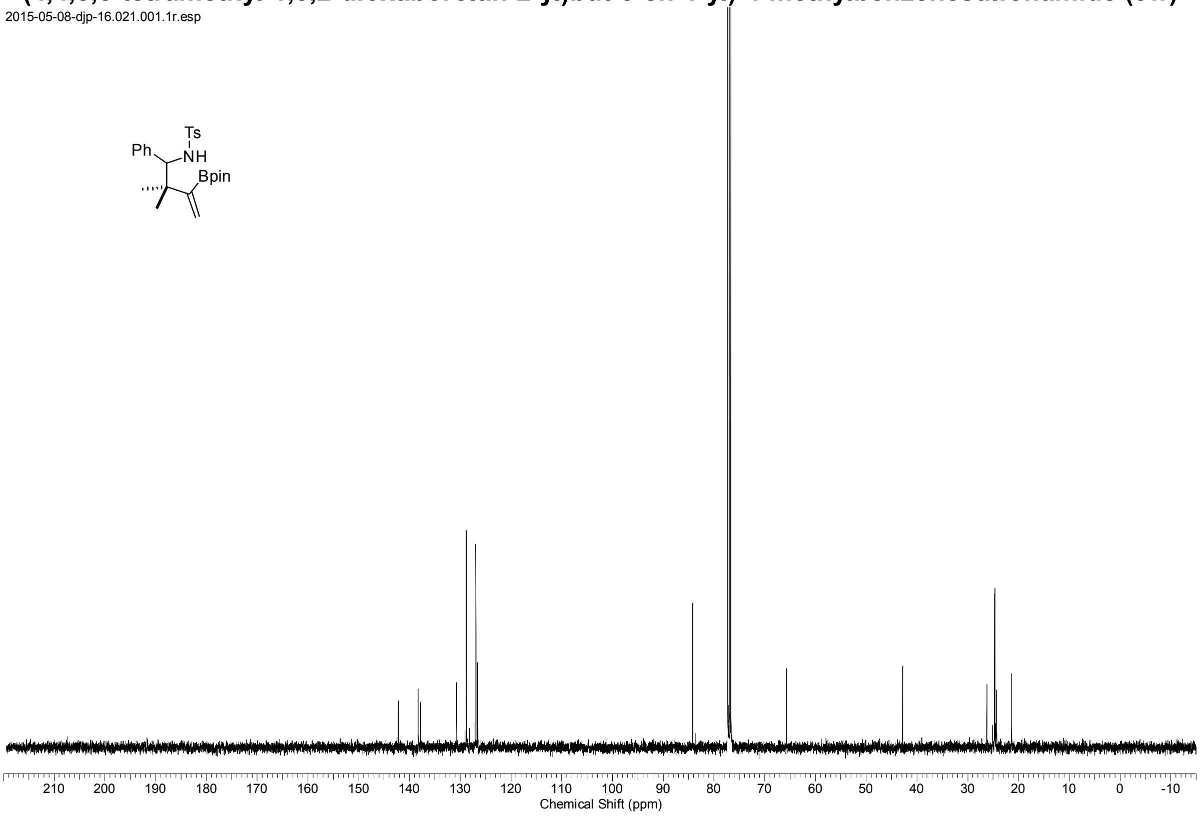

rac-*N*-((1*R*,2*S*)-3-(Dimethyl(phenyl)silyl)-1,2-diphenylbut-3-en-1-yl)aniline (3x)

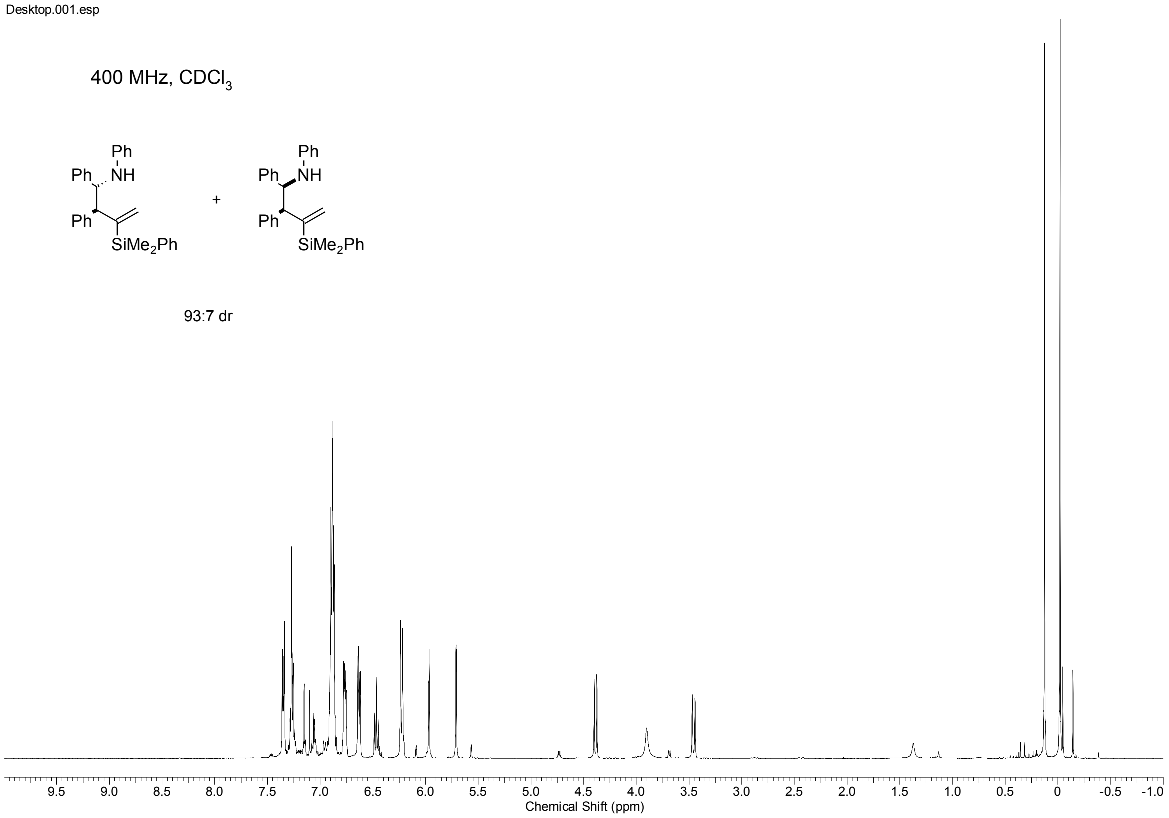

rac-*N*-((1*R*,2*S*)-3-(Dimethyl(phenyl)silyl)-1,2-diphenylbut-3-en-1-yl)aniline (3x)

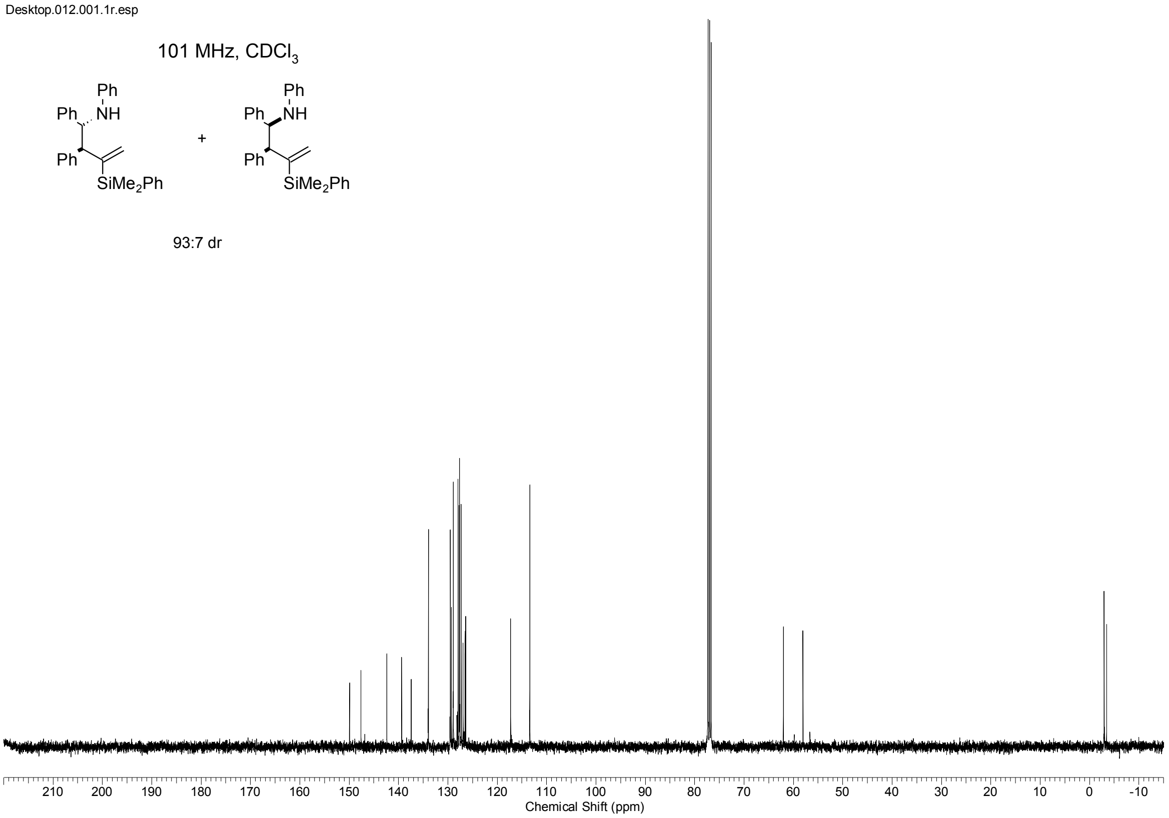

X-Ray structures:

***N*-(Phenyl(1-(1-(4,4,5,5-tetramethyl-1,3,2-dioxaborolan-2-yl)vinyl)cyclohexyl)methyl)aniline (3v)**

CCDC 1425291

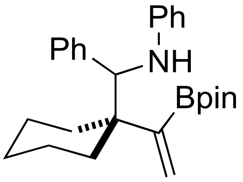

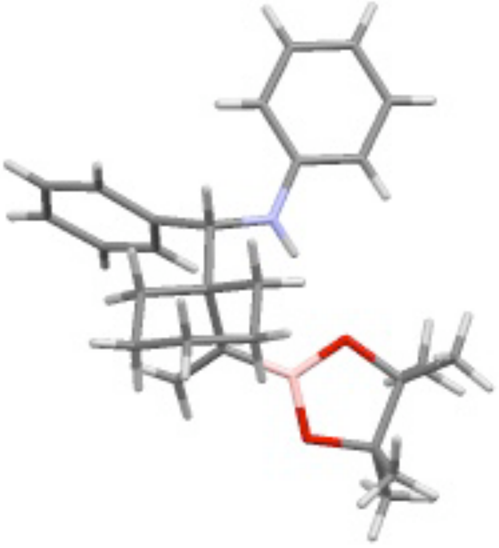

1-(1-((Benzylamino)(phenyl)methyl)cyclohexyl)ethan-1-one (4b)

CCDC 1425292

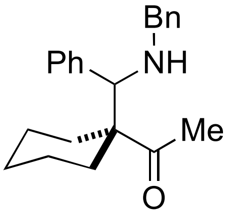

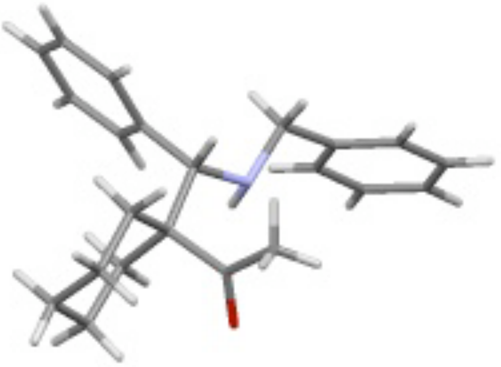

***rac-N*-((1*R*,2*R*)-2-Cyclohexyl-3-(4,4,5,5-tetramethyl-1,3,2-dioxaborolan-2-yl)-1-(*o*-tolyl)but-3-en-1-yl)-4-methoxybenzenaminium trifluoromethanesulfonate (5a)**

CCDC 1425293

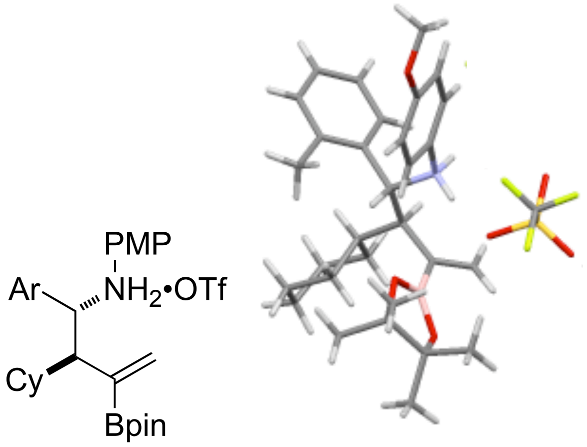

***N*-(2,2-Dimethyl-1-phenyl-3-(4,4,5,5-tetramethyl-1,3,2-dioxaborolan-2-yl)but-3-en-1-yl)-4-methylbenzenesulfonamide (3w)**

CCDC 1425294

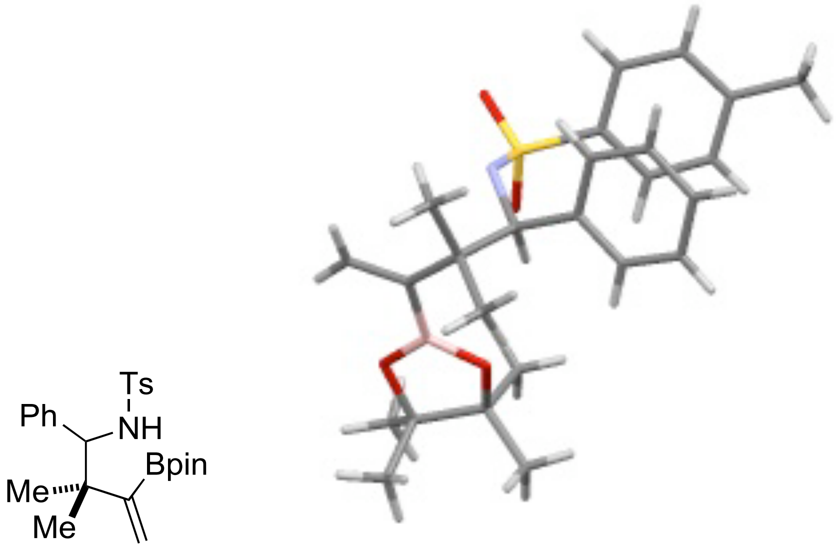

Computational studies:

The effect of solvent polarization was modelled in THF. While the details of the reaction remain unchanged, the energetics are slightly modified. Figure S1 summarises both sets of results.

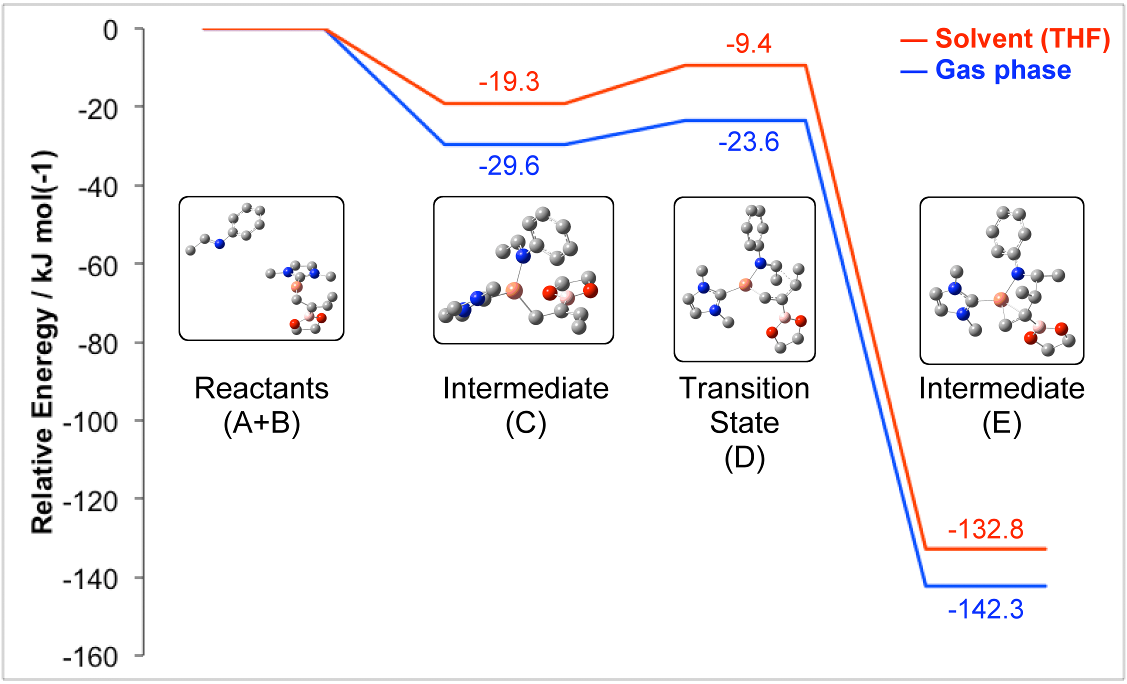

Figure S1 Modelling the effect of solvent polarization on the coupling of model substrates **A** and **B**. Hydrogen atoms have been omitted for clarity.

Alternative half chair-like transition structures, in which the imine nitrogen interacted with boron were calculated to be much higher in energy. Of these, the *anti*-transition structure **8b** was calculated to be higher in energy than the analogous *syn*-transition structure described below.

Alternative Reaction Path

A higher energy reaction path involving initial coordination of the imine with boron on the Bpin unit was located. The structures involved (**F** – **I**) are shown below. Figure S.1 summarises the energetic requirements of the mechanism (B3LYP/6-31G(d,p), gas phase).

The reactants **A** + **B** (see main text) form an intermediate **F** with the imine nitrogen coordinating to boron.

(**F**)

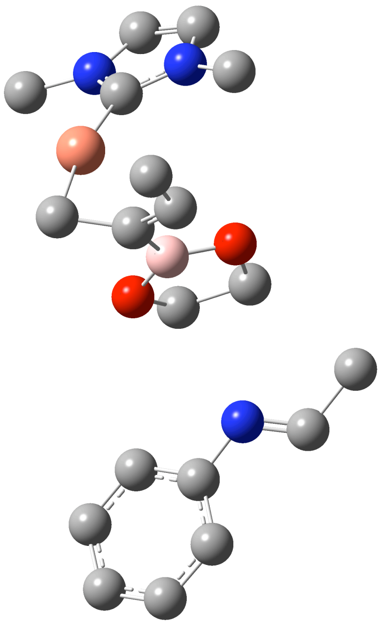

The 5-membered ring transition state, **G**, is formed at significant energetic cost (+67.4 kJ mol^{-1}).

(**G**)

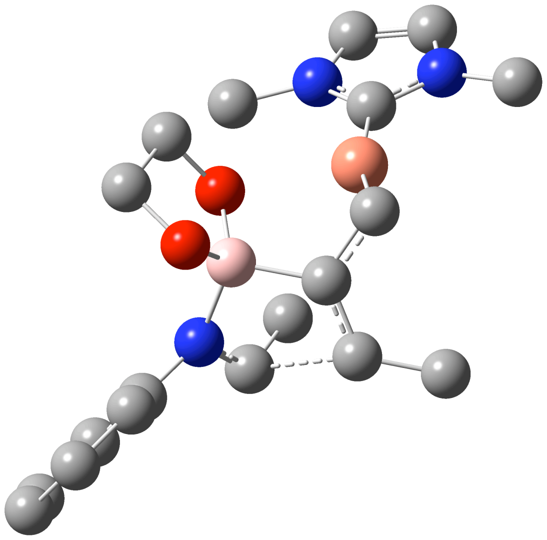

Transition structure **G** leads to the intermediate structure, **H**.

(**H**)

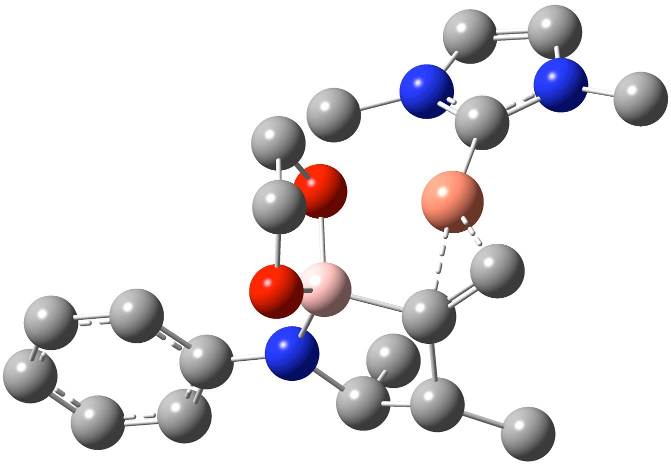

The Cu coordinates at the centre of the C=C in **H**. To proceed the coordination of the Cu moves to the imine nitrogen and the bond between the imine nitrogen and boron breaks in the transition state **I**. The barrier here is only 30.3 kJ mol^{-1}.

(**I**)

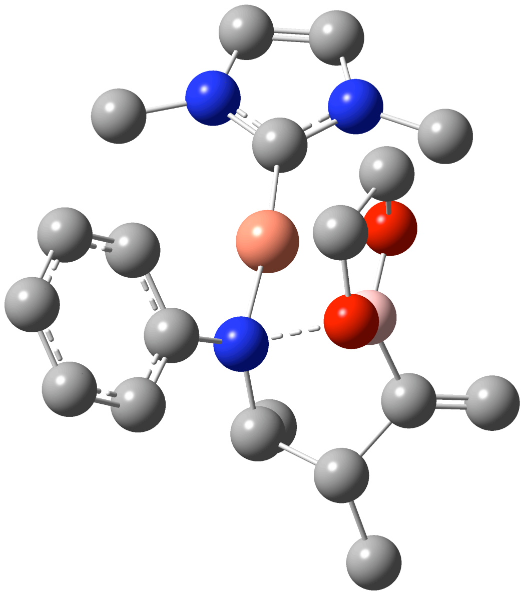

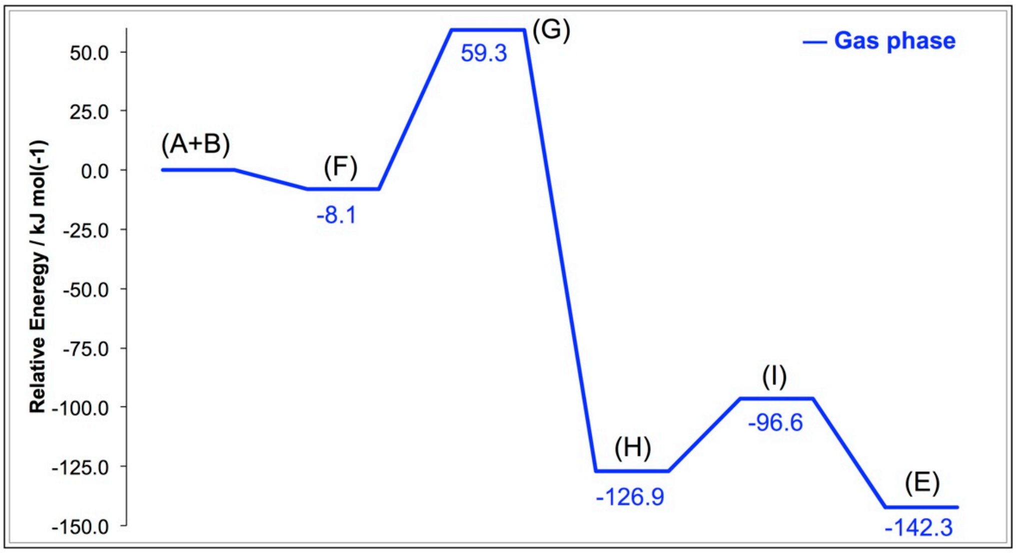

Figure S2 Energy profile for alternative mechanism (B3LYP/6-31G(d,p), gas phase).

Table S1 Cartesian coordinates in the gas phase and in PCM solvent (THF) for all optimized structures (see main text) and **F – I** in SI.

A. (gas phase)

18

B3LYP/6-31G(d,p): Energy = -365.022959 au

C	0.642991	-0.028272	0.345904
N	-0.467762	0.078476	-0.271030
H	1.053112	0.799194	0.946613
C	-1.230643	1.259124	-0.137887
C	-1.850824	1.772728	-1.287436
C	-1.437948	1.900913	1.094071
C	-2.622268	2.929247	-1.213619
H	-1.705065	1.254036	-2.229583
C	-2.227330	3.048705	1.164144
H	-1.006278	1.478266	1.996475
C	-2.814522	3.573056	0.011997
H	-3.084779	3.324437	-2.113578
H	-2.388704	3.530161	2.124605
H	-3.429460	4.466089	0.069988
C	1.464317	-1.278467	0.284383
H	0.975279	-2.024442	-0.344478
H	2.464135	-1.064176	-0.113037
H	1.609253	-1.691485	1.290604

B. (gas phase)

35

B3LYP/6-31G(d,p): Energy = -2355.199987 au

C	-2.475041	-1.349094	2.042998
C	-2.133565	-1.281939	0.722264
B	-2.033751	0.124386	0.073110
O	-2.153987	0.360383	-1.284467
C	-1.899506	1.747833	-1.520218
Cu	0.022691	-1.518112	-0.075800

O	-1.829544	1.296060	0.788350
C	-1.960377	2.395579	-0.119703
C	1.477320	-0.362105	0.139834
N	2.481486	-0.098081	-0.744942
C	3.365020	0.845069	-0.246365
C	2.907456	1.185482	0.986530
N	1.759530	0.441698	1.203918
C	-1.681473	-2.406916	-0.139453
H	-2.680283	-0.419712	2.571652
H	4.226191	1.186587	-0.798807
H	3.294923	1.880286	1.714901
H	-2.113131	-2.363989	-1.143856
H	-1.816577	-3.401878	0.289153
H	-1.153423	3.115467	0.052152
H	-0.909554	1.858416	-1.981665
H	-2.648354	2.148917	-2.210195
H	-2.917739	2.900887	0.060258
C	-2.572882	-2.617920	2.841650
H	-3.230685	-2.498823	3.709377
H	-2.956563	-3.446555	2.235163
H	-1.593087	-2.945406	3.226595
C	2.595543	-0.750106	-2.042655
H	3.547383	-1.282804	-2.121717
H	1.772313	-1.462279	-2.115934
H	2.519186	-0.018728	-2.852298
C	0.953099	0.500249	2.419392
H	0.614647	1.523019	2.599083
H	0.078629	-0.134484	2.278310
H	1.535322	0.147325	3.275295

C. (gas phase)

53

B3LYP/6-31G(d,p): Energy = -2720.234206 au

C	2.101085	-0.105211	2.620792
C	1.818693	-0.899325	1.548800
B	2.982692	-1.152380	0.555715
O	4.277678	-0.693290	0.732471
C	5.042916	-1.029413	-0.424984
Cu	-0.696268	-0.515802	0.006326
O	2.858437	-1.888801	-0.617595
C	4.159546	-2.035193	-1.196420
C	-2.570743	-0.876622	0.046550
N	-3.598240	-0.104609	0.516380
C	-4.800001	-0.800902	0.538911
C	-4.535768	-2.042457	0.061755
N	-3.179144	-2.071428	-0.230358
C	0.493797	-1.495092	1.274063
H	-5.720864	-0.361861	0.889552
H	-5.183294	-2.891538	-0.091486
H	-0.071930	-1.652265	2.201411
H	0.620651	-2.482916	0.806731

H	4.113734	-1.826604	-2.270502
H	5.237704	-0.119914	-1.008494
H	6.005676	-1.454811	-0.123125
H	4.498407	-3.070499	-1.062234
H	3.100811	0.312406	2.715010
C	0.581576	0.419559	-2.458902
H	1.059873	1.192344	-3.067225
N	0.099563	0.719022	-1.298777
C	-3.429292	1.263864	0.976590
H	-2.433220	1.597037	0.685608
H	-4.175537	1.916290	0.514076
H	-3.522387	1.326361	2.065630
C	-2.455947	-3.217695	-0.753327
H	-2.623694	-3.339275	-1.829024
H	-1.395618	-3.041448	-0.566959
H	-2.766755	-4.129708	-0.236791
C	1.110627	0.244028	3.697803
H	1.568716	0.833232	4.498350
H	0.262271	0.825723	3.301962
H	0.668457	-0.653104	4.153218
C	0.137191	2.082077	-0.884558
C	-0.363207	3.108151	-1.699571
C	0.634911	2.383038	0.391809
C	-0.351917	4.426791	-1.245298
H	-0.780680	2.862303	-2.671371
C	0.652748	3.707512	0.828295
H	1.055172	1.585984	0.999749
C	0.157509	4.732220	0.018333
H	-0.747416	5.215335	-1.879393
H	1.058815	3.937142	1.809209
H	0.165454	5.759775	0.369576
C	0.521482	-0.984308	-2.965685
H	0.903861	-1.061516	-3.986513
H	1.116503	-1.633720	-2.309676
H	-0.511818	-1.354903	-2.948773

D. (gas phase)

53

B3LYP/6-31G(d,p): Energy = -2720.231949 au

C	0.837198	-2.773180	-0.698937
C	1.438166	-1.553918	-0.986273
B	2.880536	-1.364864	-0.452960
O	3.592042	-0.173366	-0.533219
C	4.930562	-0.416762	-0.085356
Cu	-0.171967	0.570464	-0.152695
O	3.628598	-2.364395	0.145840
C	4.865319	-1.803707	0.591850
C	-0.089594	2.466621	0.044591
N	-1.130731	3.355069	-0.010350
C	-0.696691	4.667939	0.088524
C	0.652365	4.617293	0.217620

N	1.003854	3.276316	0.185635
C	0.790978	-0.446647	-1.645744
H	-1.372072	5.508357	0.056266
H	1.380816	5.405205	0.327633
H	0.019512	-0.714478	-2.373372
H	1.482481	0.282548	-2.071899
H	5.694105	-2.457345	0.301912
H	5.605132	-0.409394	-0.950551
H	5.242291	0.377965	0.600324
H	4.853840	-1.727630	1.686762
H	1.448907	-3.519212	-0.195698
C	-0.391512	-1.877340	1.300373
H	-0.863358	-2.851151	1.439385
N	-1.081346	-0.899621	0.740545
C	-2.435320	-1.137918	0.409969
C	-3.307977	-1.800601	1.293675
C	-2.968234	-0.633741	-0.792340
C	-4.654420	-1.967651	0.974161
H	-2.927140	-2.157932	2.245131
C	-4.315170	-0.800720	-1.103080
H	-2.299944	-0.142345	-1.492745
C	-5.169735	-1.471269	-0.224370
H	-5.307379	-2.481200	1.674828
H	-4.696914	-0.414758	-2.044518
H	-6.219084	-1.603443	-0.469489
C	0.800293	-1.565847	2.156106
H	1.511338	-2.393830	2.185276
H	1.313560	-0.673131	1.788486
H	0.473413	-1.351760	3.185497
C	2.369332	2.788608	0.307516
H	2.411915	1.757784	-0.042154
H	3.035613	3.398731	-0.308526
H	2.706467	2.833661	1.348697
C	-2.522008	2.955288	-0.143078
H	-2.919425	3.233853	-1.124314
H	-2.566904	1.872956	-0.031692
H	-3.128560	3.423732	0.637129
C	-0.382045	-3.307268	-1.403170
H	-0.839473	-4.136969	-0.853174
H	-1.149428	-2.544238	-1.547936
H	-0.121952	-3.690454	-2.402311

E. (gas phase)

53

B3LYP/6-31G(d,p): Energy = -2720.277141 au

C	-1.637510	-1.415192	1.715474
C	-1.951655	-0.048583	1.084193
B	-2.912775	0.046069	-0.134338
O	-3.887456	-0.873244	-0.442294
C	-4.524165	-0.462791	-1.660765
Cu	-0.158153	0.557694	0.449936

O	-2.919211	1.133873	-0.995700
C	-4.034925	0.985754	-1.888520
C	1.148337	1.955626	0.217975
N	1.050217	3.126147	-0.472961
C	2.222182	3.865002	-0.388256
C	3.077354	3.143544	0.379752
N	2.405373	1.985817	0.739158
C	-1.642059	1.182257	1.679800
H	2.343657	4.819449	-0.875573
H	4.088506	3.348670	0.693932
H	-1.238194	1.242917	2.687504
H	-2.130133	2.088648	1.332663
H	-3.707010	1.167778	-2.916005
H	-4.214155	-1.136982	-2.467404
H	-5.609662	-0.532967	-1.546085
H	-4.799178	1.728332	-1.629461
H	-2.581320	-1.975257	1.763536
C	-0.656571	-2.220206	0.799162
H	-0.134730	-2.928424	1.465544
N	0.301181	-1.281536	0.192803
C	-0.128163	3.534916	-1.226365
H	0.099304	3.582573	-2.295544
H	-0.917869	2.801649	-1.060992
H	-0.470432	4.517557	-0.888984
C	2.977075	0.898983	1.527405
H	3.917615	0.567726	1.082253
H	3.147537	1.222206	2.558614
H	2.269102	0.070560	1.505454
C	1.427189	-1.806311	-0.406379
C	1.921978	-3.123793	-0.195120
C	2.195492	-0.996790	-1.288790
C	3.105903	-3.563342	-0.785050
H	1.382938	-3.808684	0.449387
C	3.375375	-1.445476	-1.867657
H	1.823471	-0.004574	-1.524591
C	3.856014	-2.736690	-1.621992
H	3.444081	-4.577674	-0.583022
H	3.919558	-0.782686	-2.537834
H	4.772860	-3.090466	-2.083437
C	-1.092964	-1.320934	3.147859
H	-0.991052	-2.322579	3.576774
H	-0.101510	-0.854451	3.170621
H	-1.756660	-0.744336	3.800483
C	-1.392438	-3.067554	-0.255203
H	-0.673975	-3.614714	-0.871267
H	-2.060800	-3.796348	0.218999
H	-1.994406	-2.439923	-0.916219

F. (gas phase)

53

B3LYP/6-31G(d,p): Energy = -2720.226017 au

C	-0.247742	1.844158	1.939588
C	-0.296651	0.535872	1.550778
B	-0.038012	0.211241	0.054917
O	0.327290	-1.041455	-0.412185
C	0.335067	-1.000709	-1.843016
Cu	-2.280248	-0.605421	1.224871
O	-0.180274	1.127646	-0.977634
C	0.264525	0.501646	-2.187540
C	-3.570264	-0.355650	-0.104928
N	-4.362022	-1.296564	-0.695725
C	-5.176004	-0.739932	-1.668481
C	-4.891561	0.588162	-1.692921
N	-3.913475	0.802926	-0.735976
C	-0.744291	-0.614551	2.380408
H	-5.873685	-1.323245	-2.248479
H	-5.295540	1.384224	-2.298311
H	-0.842667	-0.403664	3.446914
H	-0.131623	-1.507234	2.224677
H	1.247998	0.900995	-2.456883
H	1.244441	-1.479462	-2.218456
H	-0.533515	-1.554908	-2.221160
H	-0.440383	0.721193	-2.996311
H	-0.001306	2.597885	1.194017
C	3.845822	1.982757	-1.180443
H	4.834679	2.031763	-1.663872
N	3.490527	0.983951	-0.470673
C	-3.324712	2.103120	-0.430236
H	-2.530451	1.953235	0.301047
H	-2.890382	2.541722	-1.331400
H	-4.084969	2.775215	-0.022082
C	-4.343145	-2.704261	-0.321708
H	-3.620960	-2.808095	0.489561
H	-5.330393	-3.022693	0.024828
H	-4.035814	-3.326155	-1.167339
C	-0.529329	2.334238	3.331731
H	-0.131241	1.647786	4.088543
H	-0.093325	3.323274	3.508330
H	-1.610100	2.419139	3.533099
C	4.386386	-0.081297	-0.224126
C	3.826554	-1.356229	-0.044023
C	5.776866	0.079997	-0.097548
C	4.647944	-2.454912	0.198282
H	2.746294	-1.457202	-0.084674
C	6.590293	-1.022500	0.159323
H	6.215071	1.071135	-0.166685
C	6.032304	-2.295044	0.296413
H	4.203528	-3.437581	0.328384
H	7.662943	-0.884713	0.264331
H	6.669235	-3.150722	0.500502
C	2.936353	3.152890	-1.398261
H	2.834690	3.373392	-2.468242

H	1.953538	2.954034	-0.966701
H	3.359356	4.053433	-0.934059

G. (gas phase)

53

B3LYP/6-31G(d,p): Energy = -2720.200369

C	0.923942	2.395691	-0.479816
C	0.463878	1.608543	0.570899
B	1.104700	0.134218	0.836008
O	1.851735	-0.035705	2.054830
C	1.626451	-1.350986	2.524192
Cu	-1.815369	0.597196	0.499713
O	0.011553	-0.899809	0.892630
C	0.189377	-1.661571	2.084372
C	-3.278698	-0.376401	-0.174854
N	-3.420660	-1.685214	-0.532669
C	-4.720734	-1.971890	-0.917342
C	-5.424165	-0.816412	-0.800470
N	-4.532054	0.140925	-0.349291
C	-0.680394	1.931741	1.381003
H	1.963381	2.309185	-0.777099
H	-5.028088	-2.955853	-1.235055
H	-6.462461	-0.599562	-0.996163
H	-0.995315	2.976425	1.407022
H	-0.636893	1.501719	2.388638
H	0.027127	-2.729606	1.886122
H	2.334883	-2.062107	2.067698
H	1.757569	-1.389708	3.612067
H	-0.534205	-1.341392	2.849855
C	1.422491	0.275914	-1.631975
H	2.094582	0.546078	-2.447527
N	2.002290	-0.132490	-0.503343
C	-2.335034	-2.659073	-0.526948
H	-2.191151	-3.066877	-1.532031
H	-1.426035	-2.153693	-0.199339
H	-2.567609	-3.478576	0.159599
C	-4.862533	1.533941	-0.078390
H	-5.624540	1.604477	0.703036
H	-3.940095	2.006919	0.263492
H	-5.221144	2.029502	-0.985108
C	3.417182	-0.280939	-0.516252
C	4.014471	-1.029293	-1.546585
C	4.240842	0.298475	0.464558
C	5.398787	-1.177985	-1.609900
H	3.381673	-1.516698	-2.282247
C	5.622989	0.135388	0.396167
H	3.789483	0.860656	1.270247
C	6.212239	-0.595785	-0.637880
H	5.838080	-1.762219	-2.413705
H	6.246541	0.595396	1.157704
H	7.290822	-0.714091	-0.681674

C	0.018217	-0.042399	-2.040968
H	-0.017265	-1.055958	-2.471731
H	-0.683487	-0.008429	-1.207229
H	-0.317184	0.661825	-2.806845
C	0.209752	3.629895	-0.963116
H	0.229910	4.441096	-0.218112
H	0.657293	4.024276	-1.881362
H	-0.851386	3.427882	-1.164691

H. (gas phase)

53

B3LYP/6-31G(d,p): Energy = -2720.271290

C	-0.461755	-2.158349	-1.637915
C	0.056344	-2.069195	-0.219374
B	-1.037305	-1.177354	0.629997
O	-1.841103	-1.884615	1.617584
C	-1.193361	-1.804172	2.863297
Cu	1.456780	-0.575041	0.246452
O	-0.224129	-0.177441	1.490202
C	-0.508120	-0.424365	2.864056
C	2.684068	0.872535	0.125910
N	2.383411	2.198999	0.211266
C	3.511830	2.984885	0.053628
C	4.556510	2.135598	-0.126001
N	4.032235	0.854310	-0.078847
C	1.210702	-2.655800	0.227513
H	-1.240646	-2.935581	-1.587181
H	3.476405	4.062449	0.084294
H	5.606789	2.329957	-0.275435
H	1.827262	-3.281726	-0.416577
H	1.413734	-2.762605	1.294706
H	-1.184807	0.353345	3.246029
H	-1.920632	-1.909598	3.678369
H	-0.443380	-2.606748	2.983201
H	0.411807	-0.403046	3.463072
C	-1.228237	-0.822167	-1.836781
H	-2.020937	-0.971334	-2.582345
N	-1.810434	-0.539068	-0.521200
C	1.031779	2.720441	0.414316
H	0.377185	1.889545	0.680281
H	1.038804	3.451155	1.226831
H	0.668687	3.199366	-0.499020
C	4.812636	-0.366928	-0.221890
H	5.545566	-0.453000	0.585261
H	4.114835	-1.202887	-0.171008
H	5.330176	-0.380403	-1.185087
C	-2.887417	0.335294	-0.417193
C	-3.450466	0.998767	-1.533997
C	-3.482412	0.596496	0.842106
C	-4.528735	1.873013	-1.391895
H	-3.051435	0.836303	-2.528011

C	-4.551421	1.474184	0.968231
H	-3.118985	0.060556	1.707436
C	-5.089653	2.130354	-0.143430
H	-4.928950	2.358242	-2.279183
H	-4.979856	1.639429	1.954203
H	-5.926682	2.813760	-0.037378
C	-0.305594	0.315303	-2.314242
H	-0.831740	1.272817	-2.305365
H	0.548385	0.397521	-1.628977
H	0.083127	0.143650	-3.323738
C	0.513443	-2.547913	-2.749796
H	0.908859	-3.555352	-2.584231
H	0.014736	-2.550407	-3.725235
H	1.366132	-1.863558	-2.812098

I. (gas phase)

53

B3LYP/6-31G(d,p): Energy = -2720.259742

C	-2.312992	-2.054922	-0.949117
C	-1.433346	-2.432962	0.223151
B	-1.135961	-1.197720	1.148313
O	-2.151838	-0.648739	1.928722
C	-1.555660	0.177188	2.927159
Cu	0.832641	0.098529	-0.322109
O	0.128829	-0.999286	1.774770
C	-0.099796	-0.320256	3.019513
C	2.676730	0.259822	-0.217167
N	3.442233	1.279596	-0.707673
C	4.791798	1.051804	-0.495551
C	4.884739	-0.142928	0.143193
N	3.589997	-0.606822	0.307454
C	-0.944674	-3.656210	0.460155
H	-3.222452	-1.612758	-0.514123
H	5.555187	1.747959	-0.805075
H	5.745736	-0.690745	0.492348
H	-1.174866	-4.520396	-0.160203
H	-0.296713	-3.843675	1.313521
H	0.622688	0.494517	3.138901
H	-1.606202	1.227355	2.618644
H	-2.098732	0.060773	3.871186
H	0.040892	-1.033123	3.842514
C	-1.577010	-0.875244	-1.664823
H	-2.299448	-0.349592	-2.302488
N	-1.037350	0.055802	-0.649437
C	2.887059	2.452232	-1.369294
H	3.151668	3.364215	-0.826633
H	3.251134	2.522717	-2.398138
H	1.803650	2.332257	-1.375022
C	3.234368	-1.878913	0.927523
H	3.799848	-2.008031	1.854072
H	2.168238	-1.856889	1.153106

H	3.455335	-2.711252	0.252567
C	-1.731580	1.250295	-0.430844
C	-3.105699	1.452669	-0.687514
C	-1.023391	2.342071	0.125720
C	-3.715485	2.676488	-0.414290
H	-3.709377	0.643023	-1.082237
C	-1.639361	3.555243	0.408500
H	0.041713	2.204205	0.303917
C	-2.998131	3.739503	0.136899
H	-4.775719	2.793344	-0.625481
H	-1.051920	4.367742	0.829898
H	-3.483915	4.686999	0.348436
C	-0.439116	-1.360333	-2.572684
H	0.092951	-0.502429	-2.993778
H	0.278585	-1.972140	-2.014505
H	-0.815053	-1.964787	-3.404601
C	-2.750013	-3.188517	-1.877966
H	-3.372831	-3.911651	-1.341147
H	-3.339853	-2.800330	-2.716263
H	-1.898076	-3.734057	-2.295508

A. PCM-(THF)

18

B3LYP/6-31G(d,p) PCM(Solvent=THF): Energy = -365.026985 au

C	0.640055	-0.032887	0.342914
N	-0.471809	0.073122	-0.276736
H	1.047258	0.795689	0.941434
C	-1.231575	1.257931	-0.142160
C	-1.853072	1.774919	-1.290332
C	-1.436105	1.898469	1.091583
C	-2.625218	2.931932	-1.213658
H	-1.708888	1.261836	-2.236178
C	-2.224734	3.047495	1.164493
H	-1.000932	1.476837	1.992661
C	-2.815252	3.573880	0.013959
H	-3.088278	3.328836	-2.112471
H	-2.382577	3.528071	2.125847
H	-3.429424	4.467171	0.074483
C	1.464814	-1.279293	0.287930
H	0.986590	-2.034292	-0.338952
H	2.464917	-1.058662	-0.104294
H	1.607732	-1.683465	1.297614

B. PCM-(THF)

35

B3LYP/6-31G(d,p) PCM(Solvent=THF): Energy = -2355.212701 au

C	-2.530576	-1.348680	2.033296
C	-2.147415	-1.295712	0.724078
B	-2.029559	0.104020	0.056388
O	-2.129788	0.314890	-1.307212
C	-1.874576	1.707496	-1.559135

Cu	0.018428	-1.490097	-0.041718
O	-1.860190	1.282606	0.759495
C	-1.966903	2.373808	-0.170905
C	1.481348	-0.332275	0.154372
N	2.462625	-0.058362	-0.750220
C	3.371950	0.858595	-0.250541
C	2.954757	1.170302	1.004458
N	1.802638	0.436365	1.232342
C	-1.663725	-2.430409	-0.109385
H	-2.746604	-0.412011	2.544852
H	4.220375	1.204312	-0.819470
H	3.371177	1.838171	1.741680
H	-2.089861	-2.417698	-1.118505
H	-1.797462	-3.417944	0.339117
H	-1.161497	3.090897	0.010401
H	-0.876899	1.811819	-2.001175
H	-2.612533	2.092882	-2.267787
H	-2.926869	2.880623	-0.018590
C	-2.641605	-2.601612	2.856132
H	-3.368857	-2.487209	3.667684
H	-2.944472	-3.462102	2.248227
H	-1.683906	-2.876333	3.327845
C	2.537829	-0.661515	-2.076174
H	3.498396	-1.164949	-2.209157
H	1.730940	-1.391044	-2.149146
H	2.415402	0.098310	-2.852145
C	1.041877	0.465619	2.477794
H	0.800459	1.497015	2.743268
H	0.117203	-0.090011	2.325710
H	1.619107	0.008947	3.286040

C. PCM-(THF)

53

B3LYP/6-31G(d,p) PCM(Solvent=THF): Energy = -2720.247023 au

C	2.242321	0.093683	2.569746
C	1.912193	-0.771513	1.566741
B	3.027916	-1.095374	0.534026
O	4.309177	-0.571076	0.576317
C	5.025368	-1.024324	-0.581793
Cu	-0.662974	-0.522418	0.083945
O	2.864448	-1.969374	-0.530244
C	4.135346	-2.134164	-1.180517
C	-2.531761	-0.954817	0.120839
N	-3.587584	-0.205186	0.559698
C	-4.773126	-0.929080	0.553193
C	-4.466676	-2.166677	0.089625
N	-3.102060	-2.164492	-0.165387
C	0.580787	-1.386777	1.395222
H	-5.712394	-0.508058	0.875651
H	-5.087928	-3.032267	-0.078399
H	0.052145	-1.465354	2.355293

H	0.685179	-2.408985	1.002320
H	4.009794	-2.040810	-2.262985
H	5.159246	-0.183493	-1.272786
H	6.013427	-1.388219	-0.285110
H	4.520269	-3.136889	-0.961192
H	3.238116	0.532024	2.579362
C	0.466847	0.394179	-2.472737
H	0.871063	1.169809	-3.128567
N	0.038653	0.704835	-1.294427
C	-3.469585	1.171805	1.016227
H	-2.477830	1.533395	0.745436
H	-4.225442	1.796902	0.533995
H	-3.591452	1.235815	2.101700
C	-2.349006	-3.297621	-0.680470
H	-2.515145	-3.429048	-1.754301
H	-1.292994	-3.096485	-0.497829
H	-2.639854	-4.212706	-0.159142
C	1.306513	0.509641	3.672865
H	1.810658	1.123158	4.426242
H	0.452772	1.093576	3.291762
H	0.866418	-0.356614	4.187158
C	0.039393	2.082816	-0.925281
C	-0.530108	3.063507	-1.751095
C	0.575781	2.444158	0.319233
C	-0.546315	4.396802	-1.339848
H	-0.977057	2.773719	-2.697153
C	0.565688	3.782334	0.713578
H	1.042026	1.680599	0.935675
C	0.002914	4.762162	-0.108553
H	-0.995028	5.149359	-1.981788
H	1.000413	4.059310	1.669702
H	-0.011815	5.800378	0.209008
C	0.440623	-1.021251	-2.949105
H	0.738848	-1.096898	-3.997000
H	1.122229	-1.630339	-2.341090
H	-0.564247	-1.446566	-2.835495

D. PCM-(THF)

53

B3LYP/6-31G(d,p) PCM(Solvent=THF): Energy = -2720.243278 au

C	0.644989	-2.820873	-0.657761
C	1.295872	-1.641145	-1.001759
B	2.767405	-1.495759	-0.529793
O	3.525640	-0.348746	-0.708803
C	4.873778	-0.628680	-0.294488
Cu	-0.174249	0.569570	-0.169560
O	3.490489	-2.500492	0.088204
C	4.776805	-1.975625	0.452913
C	0.085706	2.451278	0.044387
N	-0.791670	3.465284	-0.225941
C	-0.226251	4.711502	0.000418

C	1.039196	4.486002	0.433963
N	1.213051	3.109956	0.449805
C	0.678784	-0.526583	-1.674876
H	-0.763685	5.631539	-0.166492
H	1.818441	5.170985	0.728593
H	-0.134712	-0.773429	-2.363489
H	1.387517	0.160137	-2.140966
H	5.558667	-2.681112	0.158016
H	5.512693	-0.692046	-1.182623
H	5.239483	0.184423	0.338792
H	4.815633	-1.848639	1.541122
H	1.233437	-3.579689	-0.145474
C	-0.505015	-1.835246	1.328574
H	-1.028223	-2.777755	1.495925
N	-1.152100	-0.830306	0.763039
C	-2.523138	-1.001370	0.457187
C	-3.413508	-1.617396	1.357343
C	-3.049045	-0.472301	-0.737351
C	-4.773021	-1.714808	1.060145
H	-3.037579	-1.994493	2.303184
C	-4.408070	-0.568461	-1.025985
H	-2.368649	-0.017590	-1.450231
C	-5.281896	-1.192939	-0.130927
H	-5.439809	-2.192484	1.772896
H	-4.785004	-0.161308	-1.960169
H	-6.340968	-1.268568	-0.357257
C	0.710582	-1.573835	2.167566
H	1.369412	-2.442937	2.216596
H	1.276883	-0.724552	1.776935
H	0.403420	-1.317670	3.193223
C	2.428823	2.445651	0.901206
H	2.552513	1.510598	0.353859
H	3.286211	3.091128	0.700349
H	2.384775	2.239899	1.975441
C	-2.147558	3.262119	-0.714293
H	-2.222167	3.504458	-1.778746
H	-2.407307	2.215376	-0.563990
H	-2.844984	3.889535	-0.154421
C	-0.611796	-3.320867	-1.322867
H	-1.087807	-4.120224	-0.744399
H	-1.350898	-2.529146	-1.463023
H	-0.396534	-3.734342	-2.321335

E. PCM-(THF)

53

B3LYP/6-31G(d,p) PCM(Solvent=THF): Energy = -2720.290267 au

C	-1.540974	-1.465625	1.702570
C	-1.906895	-0.109956	1.077749
B	-2.936074	-0.038675	-0.088940
O	-2.987136	1.022247	-0.974403
C	-4.168715	0.866180	-1.786393

Cu	-0.128609	0.538914	0.405007
O	-3.930939	-0.962622	-0.308750
C	-4.649827	-0.571453	-1.495208
C	1.078691	2.028175	0.160049
N	2.295123	2.197253	0.746702
C	2.876594	3.400099	0.375264
C	2.004252	4.004191	-0.471476
N	0.913157	3.154263	-0.586953
C	-1.604593	1.127785	1.659264
H	3.841923	3.714172	0.739281
H	2.064200	4.944744	-0.995760
H	-1.167313	1.203187	2.652327
H	-2.116741	2.023820	1.320100
H	-5.724140	-0.629733	-1.303897
H	-4.904699	1.619796	-1.486167
H	-3.911359	1.024472	-2.836397
H	-4.397753	-1.265621	-2.303820
H	-2.467766	-2.050051	1.786687
C	-0.579257	-2.249922	0.751946
H	-0.062027	-2.988133	1.387944
N	0.386598	-1.304709	0.168019
C	1.529511	-1.823075	-0.391737
C	1.966941	-3.175298	-0.262494
C	2.385181	-0.978489	-1.159231
C	3.163502	-3.617120	-0.827721
H	1.370251	-3.889085	0.293286
C	3.572834	-1.432021	-1.716232
H	2.081224	0.052635	-1.307687
C	3.988322	-2.761689	-1.559911
H	3.449819	-4.658444	-0.691629
H	4.182458	-0.738083	-2.292020
H	4.913996	-3.116540	-2.002864
C	-1.336733	-3.046103	-0.327199
H	-2.019718	-3.776198	0.123273
H	-1.926178	-2.382779	-0.964691
H	-0.634022	-3.588581	-0.965840
C	-0.249800	3.422485	-1.426202
H	-1.014977	2.677045	-1.209695
H	-0.642942	4.418540	-1.209086
H	0.020221	3.367118	-2.484415
C	2.899210	1.222658	1.648332
H	2.850421	1.571238	2.683733
H	2.345209	0.289773	1.545639
H	3.942121	1.055750	1.371724
C	-0.946301	-1.357663	3.113755
H	-0.801031	-2.356472	3.537180
H	0.030319	-0.861023	3.102645
H	-1.602675	-0.800289	3.789764

Preliminary studies on the development of a catalytic enantioselective variant

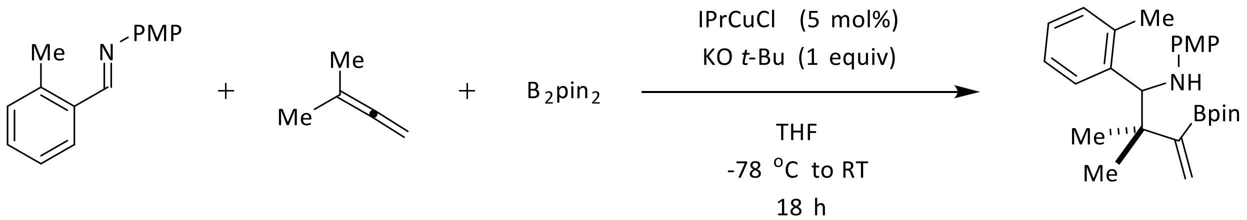

2j **1c** **S3y**

***N*-(2,2-Dimethyl-3-(4,4,5,5-tetramethyl-1,3,2-dioxaborolan-2-yl)-1-(*o*-tolyl)but-3-en-1-yl)-4-methoxyaniline (S3y)**

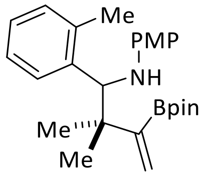

Prepared according to General Procedure 1, on a 0.258 mmol scale, column chromatography (3% EtOAc in Hexanes) afforded the title compound as a yellow gum (72 mg, 0.171 mmol, 66%). MS (ES+) *m/z*: 422 ($M+H^+$). ν_{max} (thin film/cm^{-1}): 2976, 1510, 1464, 1411, 1353, 1302, 1235, 1218, 1145, 1124, 1101, 1039; ^1H NMR (500 MHz, CDCl3) δ ppm 1.06 (s, 3 H, CH_3), 1.09 (s, 3 H, CH_3), 1.16 (s, 12 H, 4 x CH_3), 2.57 (s, 3 H, Ar-CH_3), 3.65 (s, 3 H, OCH_3), 3.96 (br. s, 1 H, NH), 4.92 (s, 1 H, CHN), 5.75 (d, J = 2.4 Hz, 1 H, C=CH_2), 5.97 (d, J = 2.6 Hz, 1 H, C=CH_2), 6.30 (d, J = 8.9 Hz, 2 H, ArCH), 6.59 (d, J = 8.9 Hz, 2 H, ArCH), 7.10 - 7.14 (m, 2 H, ArCH), 7.14 - 7.18 (m, 1 H, ArCH), 7.40 - 7.42 (m, 1 H, ArCH); ^{13}C NMR (126 MHz, $CDCl_3$) δ ppm 20.4 (Ar-CH_3), 20.6 (CH_3), 24.7 (CH_3), 24.7 (CH_3), 26.2 (CH_3), 44.8 (*C*C=CH_2), 55.8 (OCH_3), 60.3 (CHN), 83.3 (OC), 114.3 (ArCH), 114.5 (ArCH), 125.3 (ArCH), 126.3 (ArCH), 127.7 (C=*C*H_2), 129.1 (ArCH), 130.2 (ArCH), 137.0 (ArC), 139.3 (ArC), 142.8 (ArC), 151.7 (ArC), (B*C*=CH_2 not observed); ^{11}B NMR (160 MHz, $CDCl_3$) δ -2.63.

N-(2,2-Dimethyl-3-(4,4,5,5-tetramethyl-1,3,2-dioxaborolan-2-yl)-1-(o-tolyl)but-3-en-1-yl)-4-methoxyaniline (S3y)

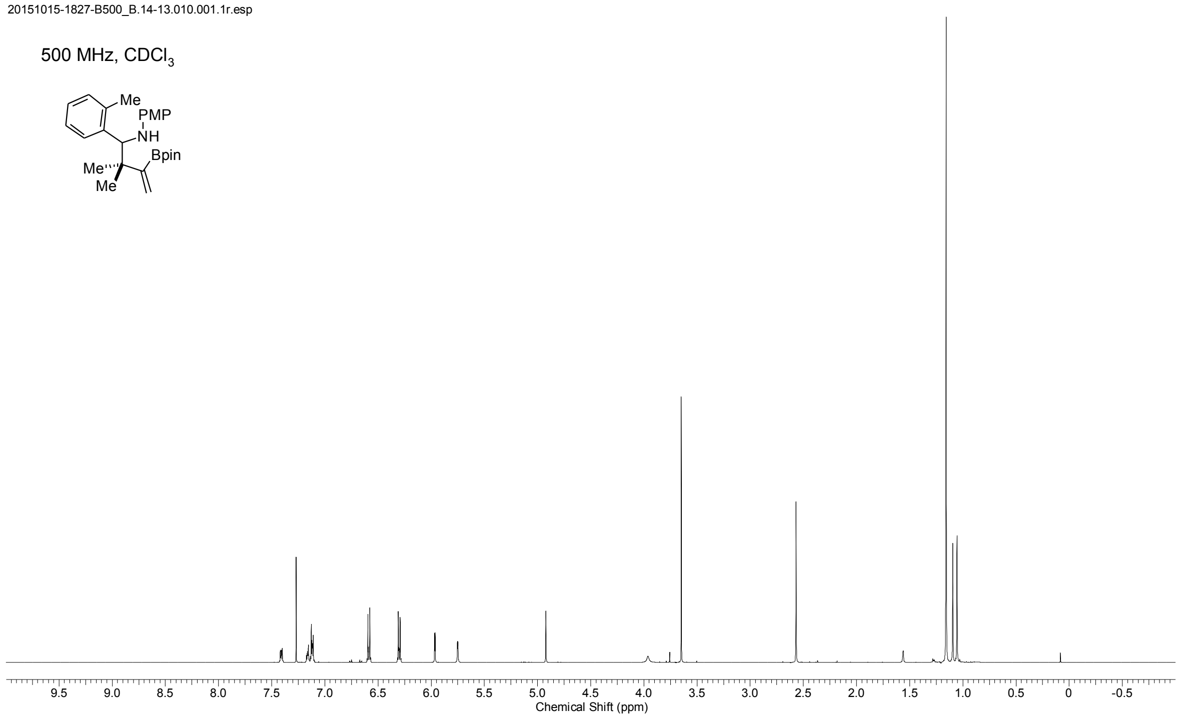

N-(2,2-Dimethyl-3-(4,4,5,5-tetramethyl-1,3,2-dioxaborolan-2-yl)-1-(o-tolyl)but-3-en-1-yl)-4-methoxyaniline (S3y)

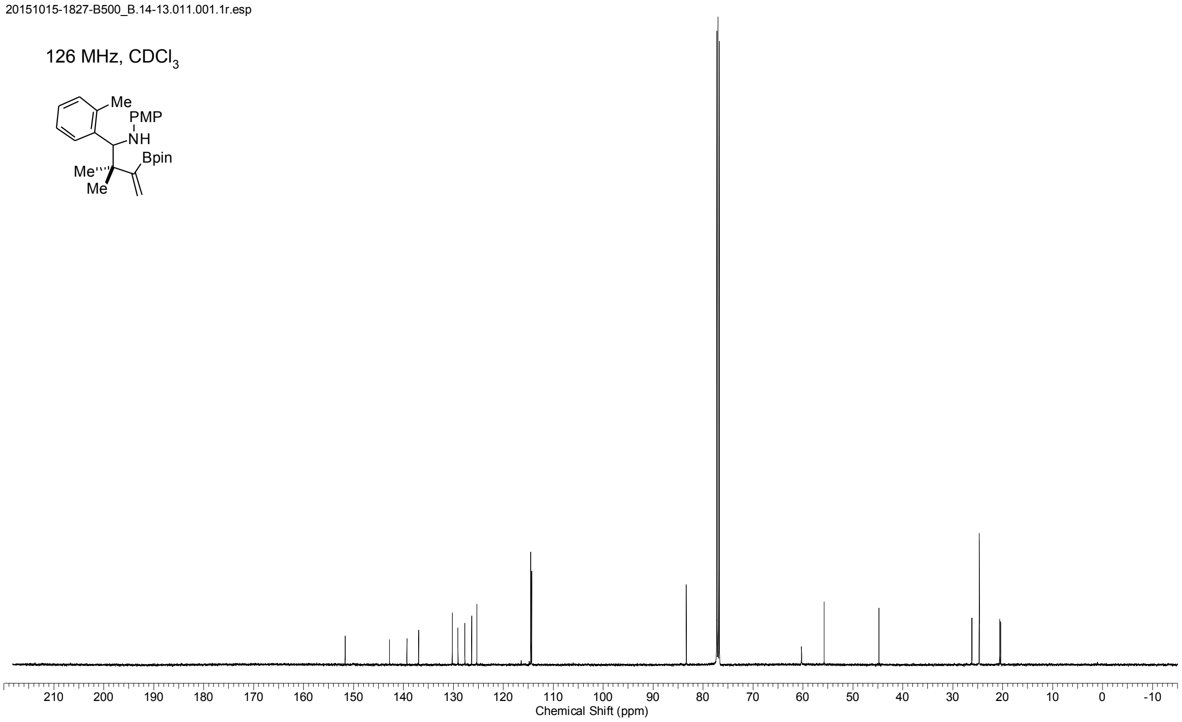

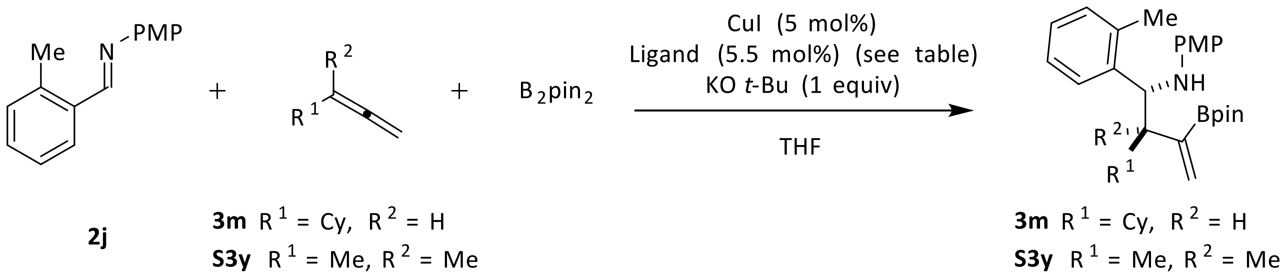

Entry	Product	Ligand	Temp (°C)	Reaction time (h)	Conv. (%)[a]	Yield (%)	% ee
1	**3m**	**L1**	-30	5	31[b]	26	34
2	**3m**	**L2**	-46 to 0	18	30[b]	19	10
3	**3m**	**L3**	-46 to 0	18	10[b]	7	3
4	**3m**	**L4**	-46 to 0	18	10[b]	8	1
5	**S3y**	**L1**	-30	5	0	-	-
6	**S3y**	**L2**	-46 to 0	18	31	28	15
7	**S3y**	**L3**	-46 to 0	18	19	15	1
8	**S3y**	**L4**	-46 to 0	18	0	-	-
9	**S3y**	**L5**	-46 to 0	18	0	-	-

Table S2 Ligand screening for asymmetric copper-catalysed borylative cross-coupling of allenes and imines. Imine (1 equiv), allene (1.5 equiv), B_2pin_2 (1.1 equiv). [a] Determined by ^{1}H NMR analysis of the crude product mixture using an internal standard. [b] >98:2 dr.
Enantiomer ratios were determined by high-performance liquid chromatography (HPLC) (Chiral Technologies Chiralpak AD–H (4.6 x 250 mm) and Chiralcel OD–H (4.6 x 250 mm)) in comparison with authentic racemic materials.

Ligands:

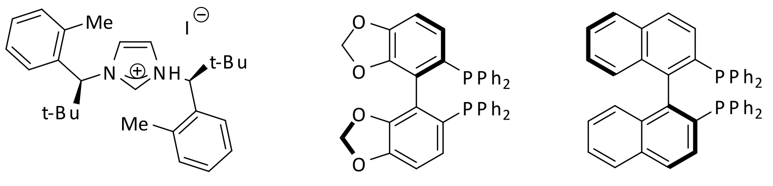

L1 L2 L3

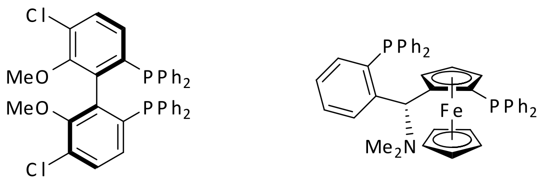

L4 L5

General procedure for asymmetric couplings

***N*-(2-Cyclohexyl-3-(4,4,5,5-tetramethyl-1,3,2-dioxaborolan-2-yl)-1-(*o*-tolyl)but-3-en-1-yl)-4-methoxyaniline (3m)**

To a solution of ligand (0.0142 mmol, 5.5 mol %) and CuI (2.5 mg, 0.013 mmol, 5.0 mol%) in THF (0.8 mL), was added KO*t*-Bu (0.26 mL of a 1 M THF solution, 0.258 mmol, 1 equiv) and the reaction was stirred for 1 hour at room temperature. B_2Pin_2 (72.1 mg, 0.284 mmol, 1.1 equiv) in THF (0.75 mL) was then added and the resulting mixture was stirred for 15 min. A solution of propa-1,2-dien-1-ylcyclohexane (47.3 mg, 0.387 mmol, 1.5 equiv) and (*E*)-*N*-(4-methoxyphenyl)-1-(*o*-tolyl)methanimine (58.1 mg, 0.258 mmol, 1 equiv) in THF (1 mL) was then added dropwise at –30 °C, and the reaction stirred for 5 hours. The mixture was then filtered through a silica plug, concentrated *in vacuo* and the crude product mixture was purified by chromatography (1 - 3% EtOAc in hexanes) to afford the title compound as a yellow gum.

HPLC data

***Rac*-*N*-((1*R*,2*R*)-2-Cyclohexyl-3-(4,4,5,5-tetramethyl-1,3,2-dioxaborolan-2-yl)-1-(*o*-tolyl)but-3-en-1-yl)-4-methoxyaniline (3m)**

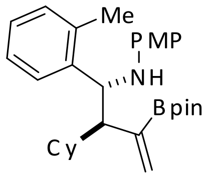

ENTRY 1

Enantiomeric purity of **3m** was determined by HPLC analysis in comparison with authentic racemic material (34% ee shown; Chiralcel AD–H column, 99:1 hexanes:*i*-PrOH, 0.3 mL/min, 20 °C, 220 nm).

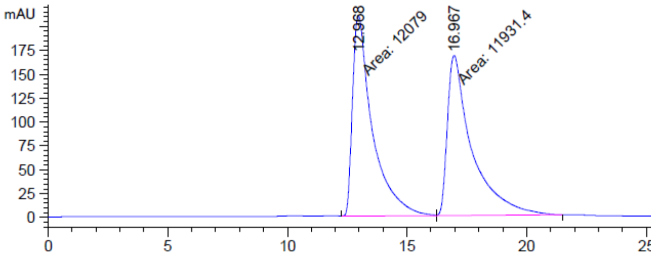

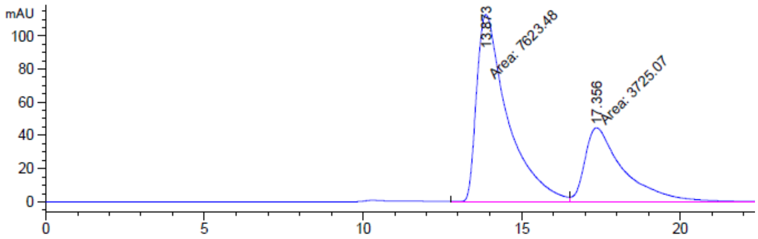

Peak #	RetTime [min]	Type	Width [min]	Area [mAU*s]	Height [mAU]	Area %
1	12.968	MF	0.9587	1.20790e4	209.98842	50.3074
2	16.967	FM	1.1881	1.19314e4	167.37422	49.6926

Peak #	RetTime [min]	Type	Width [min]	Area [mAU*s]	Height [mAU]	Area %
1	13.873	MF	1.1277	7623.47705	112.67218	67.1758
2	17.356	FM	1.3976	3725.07373	44.42375	32.8242

Peak #	Time (min)	Area (%)
1	12.968	50.3074
2	16.967	49.6926

Peak #	Time (min)	Area (%)
1	13.873	67.1758
2	17.356	32.8242

ENTRY 2

Enantiomeric purity of **3m** was determined by HPLC analysis in comparison with authentic racemic material (10% ee shown; Chiralcel AD–H column, 99:1 hexanes:*i*-PrOH, 0.3 mL/min, 20 °C, 220 nm).

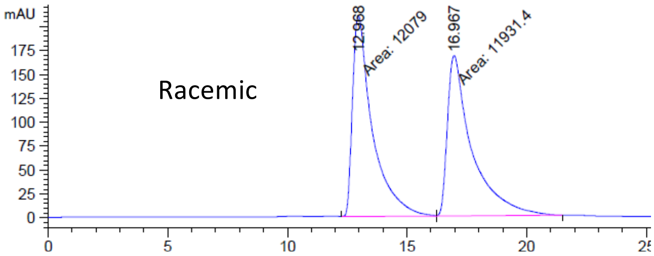

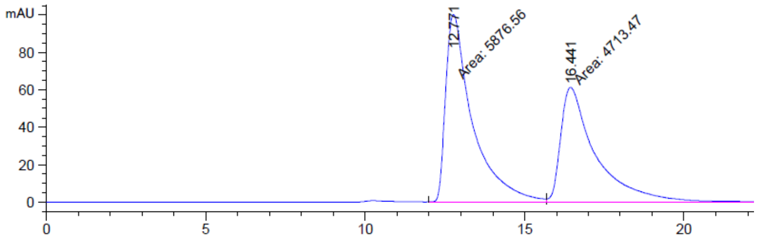

Peak #	RetTime [min]	Type	Width [min]	Area [mAU*s]	Height [mAU]	Area %
1	12.968	MF	0.9587	1.20790e4	209.98842	50.3074
2	16.967	FM	1.1881	1.19314e4	167.37422	49.6926

Peak #	RetTime [min]	Type	Width [min]	Area [mAU*s]	Height [mAU]	Area %
1	12.771	MF	0.9768	5876.55518	100.26849	55.4914
2	16.441	FM	1.2806	4713.46680	61.34469	44.5086

Peak #	Time (min)	Area (%)
1	12.968	50.3074
2	16.967	49.6926

Peak #	Time (min)	Area (%)
1	12.771	55.4914
2	16.441	44.5086

ENTRY 3

Enantiomeric purity of **3m** was determined by HPLC analysis in comparison with authentic racemic material (3% ee shown; Chiralcel AD–H column, 99:1 hexanes:*i*-PrOH, 0.3 mL/min, 20 °C, 220 nm).

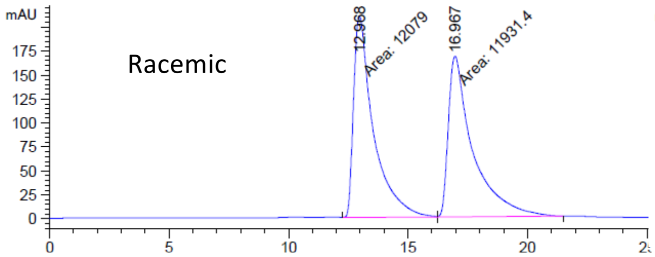

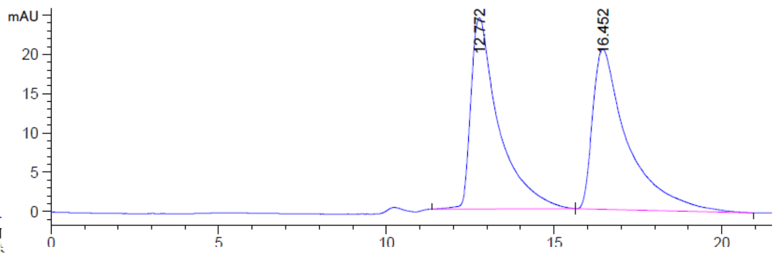

Peak #	RetTime [min]	Type	Width [min]	Area [mAU*s]	Height [mAU]	Area %
1	12.968	MF	0.9587	1.20790e4	209.98842	50.3074
2	16.967	FM	1.1881	1.19314e4	167.37422	49.6926

Peak #	RetTime [min]	Type	Width [min]	Area [mAU*s]	Height [mAU]	Area %
1	12.772	BB	0.8299	1415.10315	24.35452	48.7206
2	16.452	BB	1.0254	1489.42456	20.40893	51.2794

Peak #	Time (min)	Area (%)
1	12.968	50.3074
2	16.967	49.6926

Peak #	Time (min)	Area (%)
1	12.772	48.7206
2	16.452	51.2794

ENTRY 4

Enantiomeric purity of **3m** was determined by HPLC analysis in comparison with authentic racemic material (1% ee shown; Chiralcel AD–H column, 99:1 hexanes:*i*-PrOH, 0.3 mL/min, 20 °C, 220 nm).

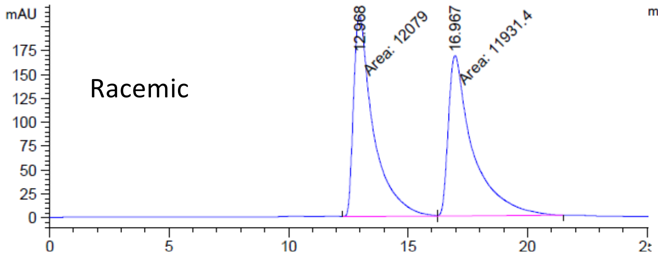

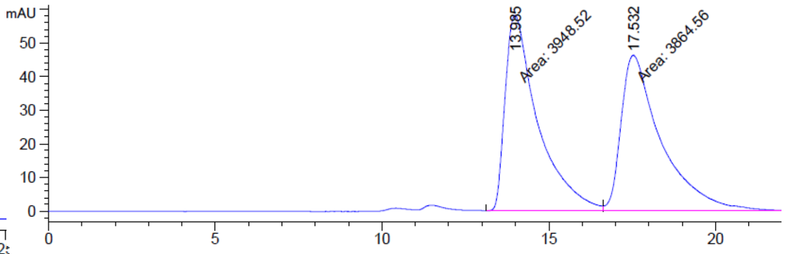

Peak #	RetTime [min]	Type	Width [min]	Area [mAU*s]	Height [mAU]	Area %
1	12.968	MF	0.9587	1.20790e4	209.98842	50.3074
2	16.967	FM	1.1881	1.19314e4	167.37422	49.6926

Peak #	RetTime [min]	Type	Width [min]	Area [mAU*s]	Height [mAU]	Area %
1	13.985	MF	1.1350	3948.52002	57.98089	50.5373
2	17.532	FM	1.3959	3864.56470	46.14167	49.4627

Peak #	Time (min)	Area (%)
1	12.968	50.3074
2	16.967	49.6926

Peak #	Time (min)	Area (%)
1	13.985	50.5373
2	17.532	49.4627

***N*-(2,2-Dimethyl-3-(4,4,5,5-tetramethyl-1,3,2-dioxaborolan-2-yl)-1-(*o*-tolyl)but-3-en-1-yl)-4-methoxyaniline (S3y)**

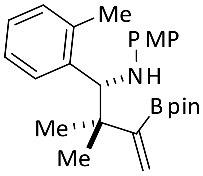

ENTRY 6

Enantiomeric purity of **S3y** was determined by HPLC analysis in comparison with authentic racemic material (15% ee shown; Chiralcel OD–H column, 99.5:0.5 hexanes:*i*-PrOH, 0.5 mL/min, 20 °C, 220 nm).

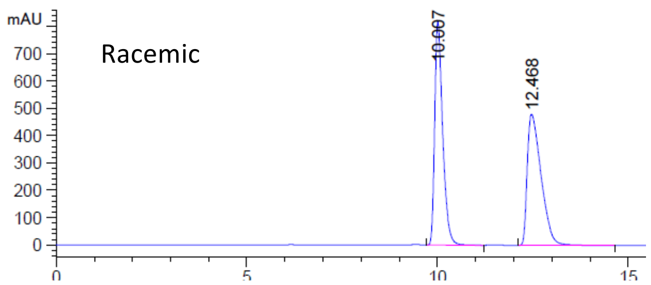

Peak #	RetTime [min]	Type	Width [min]	Area [mAU*s]	Height [mAU]	Area %
1	10.007	VB	0.2221	1.19561e4	819.93860	49.7503
2	12.468	BB	0.3900	1.20761e4	479.21451	50.2497

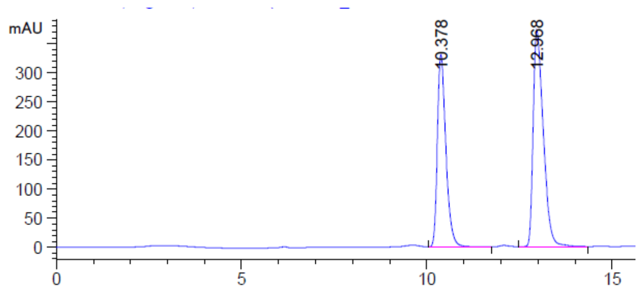

Peak #	RetTime [min]	Type	Width [min]	Area [mAU*s]	Height [mAU]	Area %
1	10.378	VB	0.2444	5273.61084	330.04318	42.6403
2	12.968	BB	0.2922	7094.06396	372.49170	57.3597

Peak #	Time (min)	Area (%)
1	10.007	49.7503
2	12.468	50.2497

Peak #	Time (min)	Area (%)
1	10.378	42.6403
2	12.968	57.3597

ENTRY 7

Enantiomeric purity of **S3y** was determined by HPLC analysis in comparison with authentic racemic material (1% ee shown; Chiralcel OD–H column, 99.5:0.5 hexanes:*i*-PrOH, 0.5 mL/min, 20 °C, 220 nm).

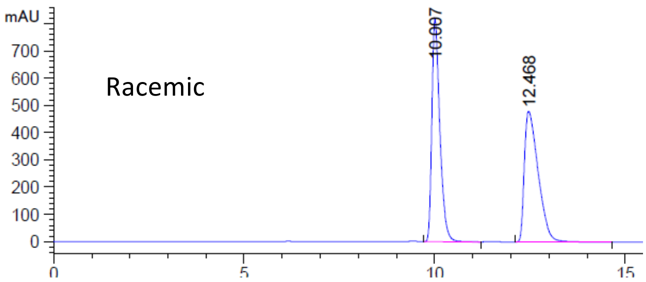

Peak #	RetTime [min]	Type	Width [min]	Area [mAU*s]	Height [mAU]	Area %
1	10.007	VB	0.2221	1.19561e4	819.93860	49.7503
2	12.468	BB	0.3900	1.20761e4	479.21451	50.2497

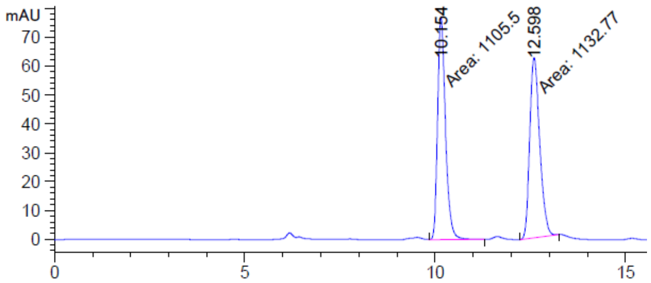

Peak #	RetTime [min]	Type	Width [min]	Area [mAU*s]	Height [mAU]	Area %
1	10.154	MM	0.2392	1105.50012	77.03753	49.3909
2	12.598	MM	0.3028	1132.76770	62.33998	50.6091

Peak #	Time (min)	Area (%)
1	10.007	49.7503
2	12.468	50.2497

Peak #	Time (min)	Area (%)
1	10.154	49.3909
2	12.598	50.6091

References:

[1] K. Semba, M. Shinomiya, T. Fujihara, J. Terao, Y. Tsuji, *Chem. – Eur. J.* **2013**, *19*, 7125–7132.

[2] A. Claesson, A. Quader, C. Sahlberg, *Tetrahedron Lett.* **1983**, *24*, 1297–1300.

[3] J. Kuang, S. Ma, *J. Org. Chem.* **2009**, *74*, 1763–1765.

[4] A. M. Seayad, B. Ramalingam, K. Yoshinaga, T. Nagata, C. L. L. Chai, *Org. Lett.* **2010**, *12*, 264–267.

[5] M. B. Shaghafi, R. E. Grote, E. R. Jarvo, *Org. Lett.* **2011**, *13*, 5188–5191.

[6] I. Ojima, I. Habus, M. Zhao, M. Zucco, Y. H. Park, C. M. Sun, T. Brigaud, *Tetrahedron* **1992**, *48*, 6985–7012.

[7] M. Patel, M. Chhasatia, P. Parmar, *Eur. J. Med. Chem.* **2010**, *45*, 439–446.

[8] S. E. Denmark, N. Nakajima, C. M. Stiff, O. J.-C. Nicaise, M. Kranz, *Adv. Synth. Catal.* **2008**, *350*, 1023–1045.

[9] A. S. Al-Tai, D. M. Hall, A. R. Mears, *J. Chem. Soc. Perkin Trans. 2* **1976**, 133–136.

[10] K. Yamada, T. Konishi, M. Nakano, S. Fujii, R. Cadou, Y. Yamamoto, K. Tomioka, *J. Org. Chem.* **2012**, *77*, 1547–1553.

[11] B. Capon, Z. P. Wu, *J. Org. Chem.* **1990**, *55*, 2317–2324.

[12] Y. Vara, T. Bello, E. Aldaba, A. Arrieta, J. L. Pizarro, M. I. Arriortua, X. Lopez, F. P. Cossío, *Org. Lett.* **2008**, *10*, 4759–4762.

[13] B. Ramalingam, A. M. Seayad, L. Chuanzhao, M. Garland, K. Yoshinaga, M. Wadamoto, T. Nagata, C. L. L. Chai, *Adv. Synth. Catal.* **2010**, *352*, 2153–2158.

[14] S. Morales, F. G. Guijarro, J. L. García Ruano, M. B. Cid, *J. Am. Chem. Soc.* **2014**, *136*, 1082–1089.
